# Supplementary material for: A Comparative Multi-System Approach to Characterizing Bioactivity of Commonly Occurring Chemicals
Source: Int J Environ Res Public Health. 2022 Mar 23;19(7):3829. doi: 10.3390/ijerph19073829 (PMC8998123; doi:10.3390/ijerph19073829)
Supplement: Supplementary file 1 [file ijerph-19-03829-s001.zip › ijerph-1630064-supplementary.pdf]

Supplementary Material: A comparative multi-system approach to characterizing bioactivity of commonly occurring chemicals

Brianna N. Rivera, Lindsay B. Wilson, Doo Nam Kim, Paritosh Pande, Kim A. Anderson, Susan C. Tilton, Robyn L. Tanguay

Contents:

Figures

S1: Correlation matrix investigating co-occurrence of real-world chemical concentrations
S2: Concentration response curves for cell viability in NHBE
S3: Concentration response curves for cytotoxicity in NHBE
S4: Concentration response bar plots of bioactive chemicals for cell viability in NHBE
S5: Concentration response bar plots NHBE Equi-Mix and components in NHBE
S6: Concentration response bar plots of bioactive chemicals for cytoxicity in NHBE
S7: Concentration response curves for Any Effect in zebrafish
S8: Concentration response bar plots for each morphological endpoint in zebrafish
S9: Zebrafish embryonic photomotor response movement versus time plots
S10: Zebrafish larval photomotor response movement versus time plots

Tables

S1: Description of morphological endpoints screened in zebrafish
S2: Percent incidence of each morphological endpoint in zebrafish
S3: Percent cell viability and cytotoxicity in NHBE
S4: Lowest effect levels for each chemical across NHBE and zebrafish screening
S5: Exposure concentrations of BMC mix components in each model system
S6: Summarized zebrafish embryonic photomotor response movement data
S7: Summarized zebrafish larval photomotor response movement data

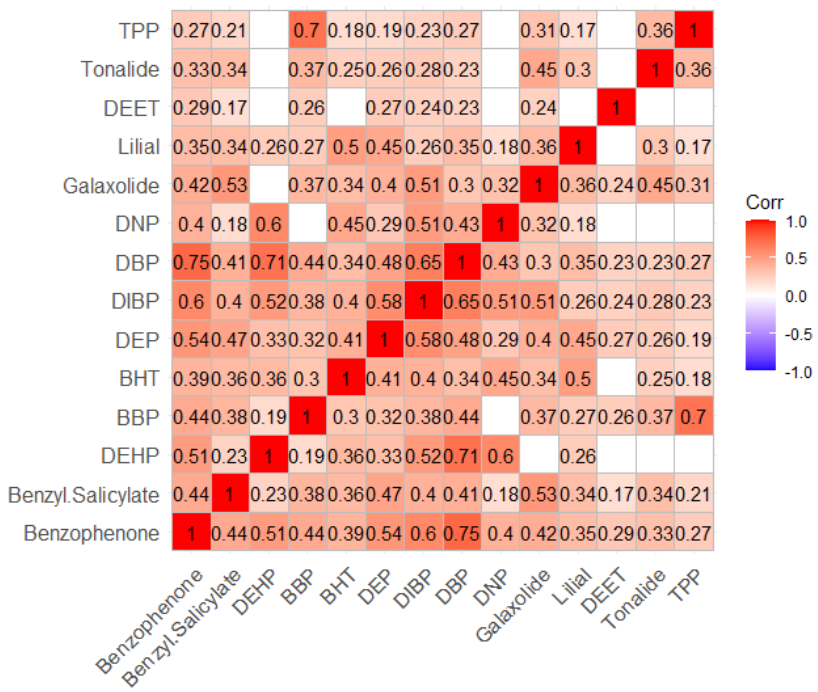

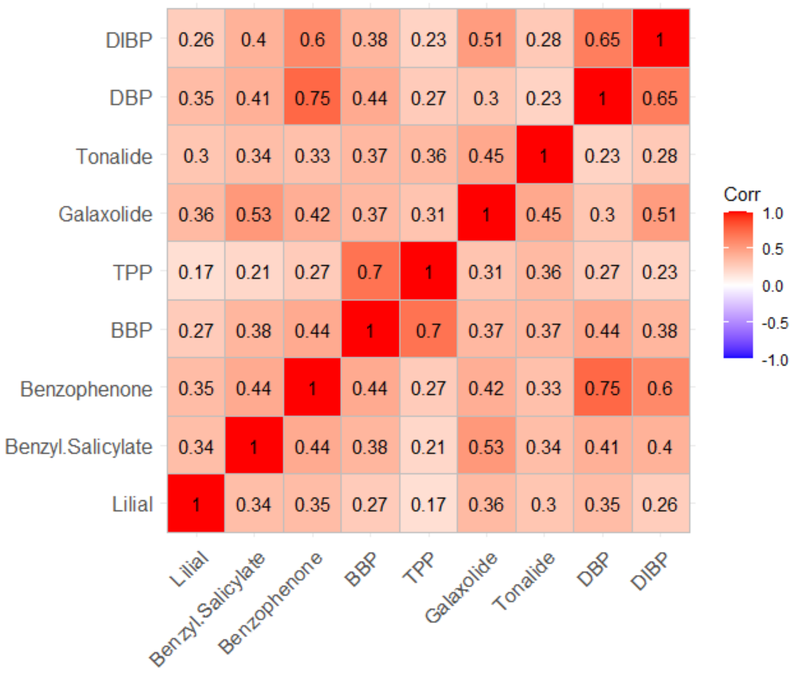

Figure S1.
Correlation matrix investigating co-occurrence of real-world chemical concentrations for A.) all G14 chemicals B.) all bioactive chemicals in zebrafish, using exposure data from Dixon et al 2019, labeled with correlation coefficients. Only chemicals with significant correlations ($p < 0.01$) are shown.

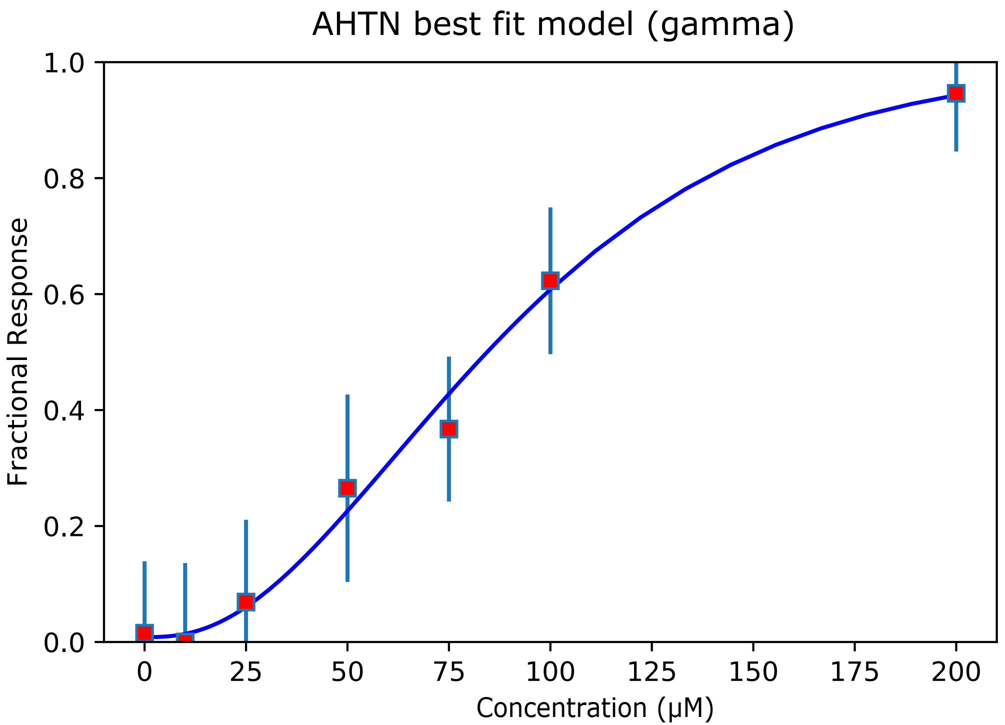
AHTN best fit model (gamma)
Fractional Response
Concentration (μM)
0.0
0.2
0.4
0.6
0.8
1.0
0
25
50
75
100
125
150
175
200

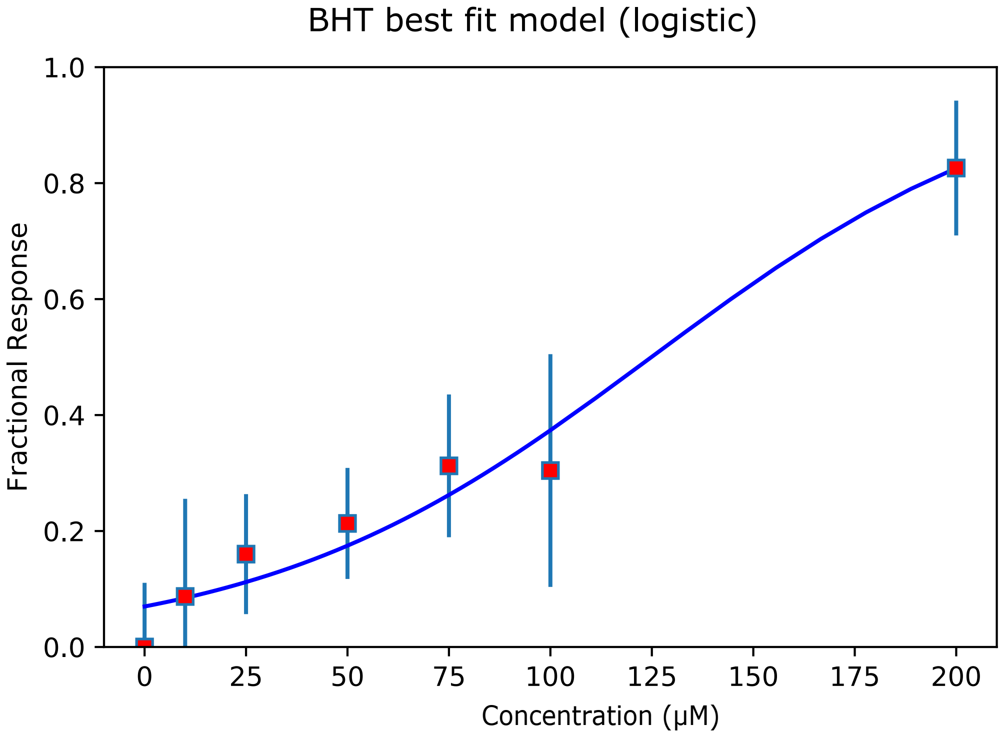

BHT best fit model (logistic)
Fractional Response
0.0
0.2
0.4
0.6
0.8
1.0
0
25
50
75
100
125
150
175
200
Concentration (μM)

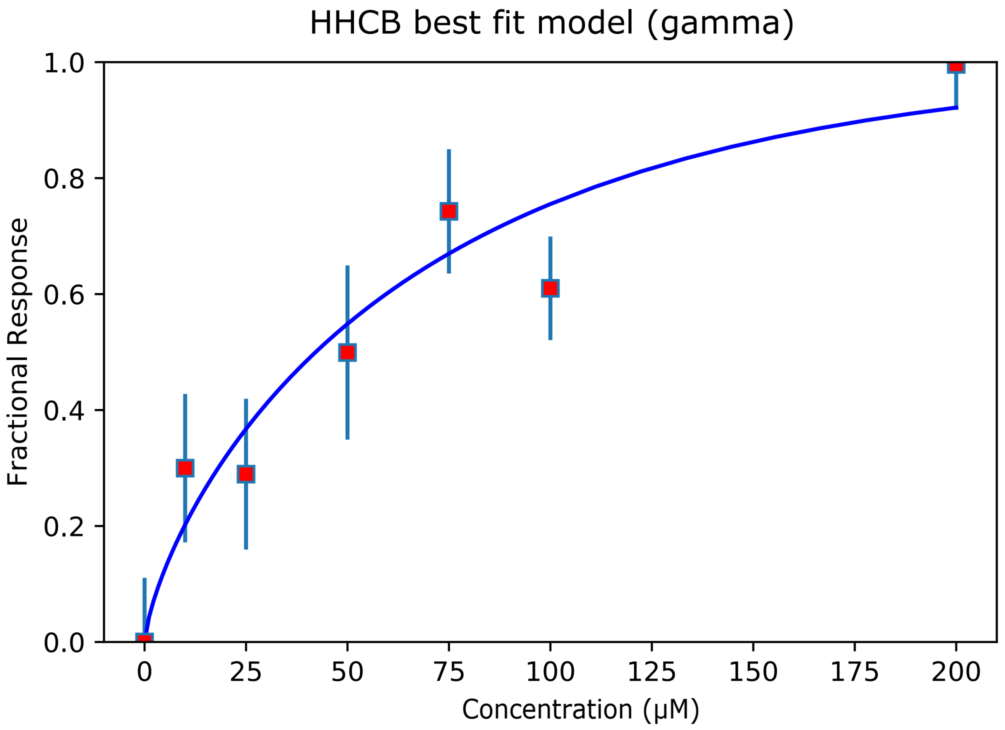
HHCB best fit model (gamma)
Fractional Response
Concentration (μM)
0.0
0.2
0.4
0.6
0.8
1.0
0
25
50
75
100
125
150
175
200

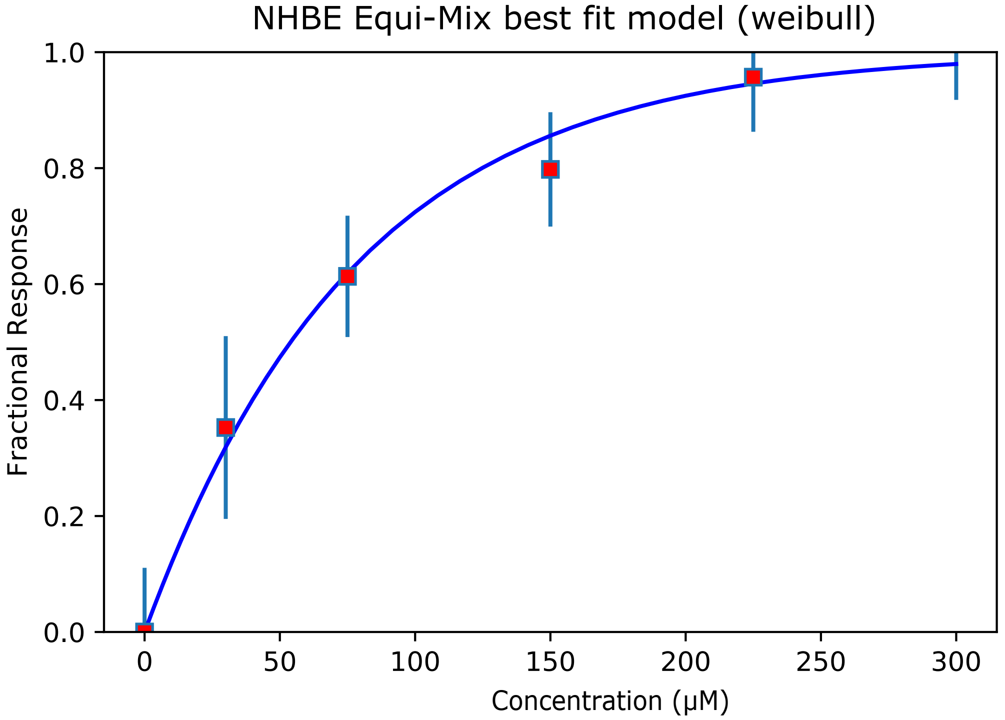
NHBE Equi-Mix best fit model (weibull)
Fractional Response
Concentration (μM)
0.0
0.2
0.4
0.6
0.8
1.0
0
50
100
150
200
250
300

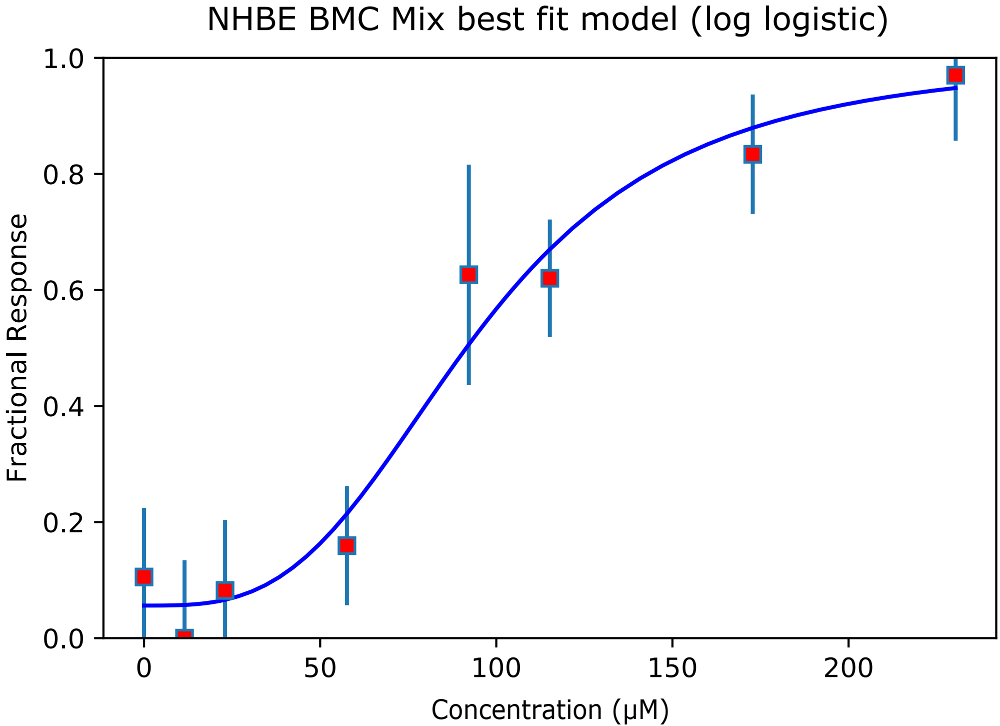
NHBE BMC Mix best fit model (log logistic)
Fractional Response
Concentration (μM)
0.0
0.2
0.4
0.6
0.8
1.0
0
50
100
150
200

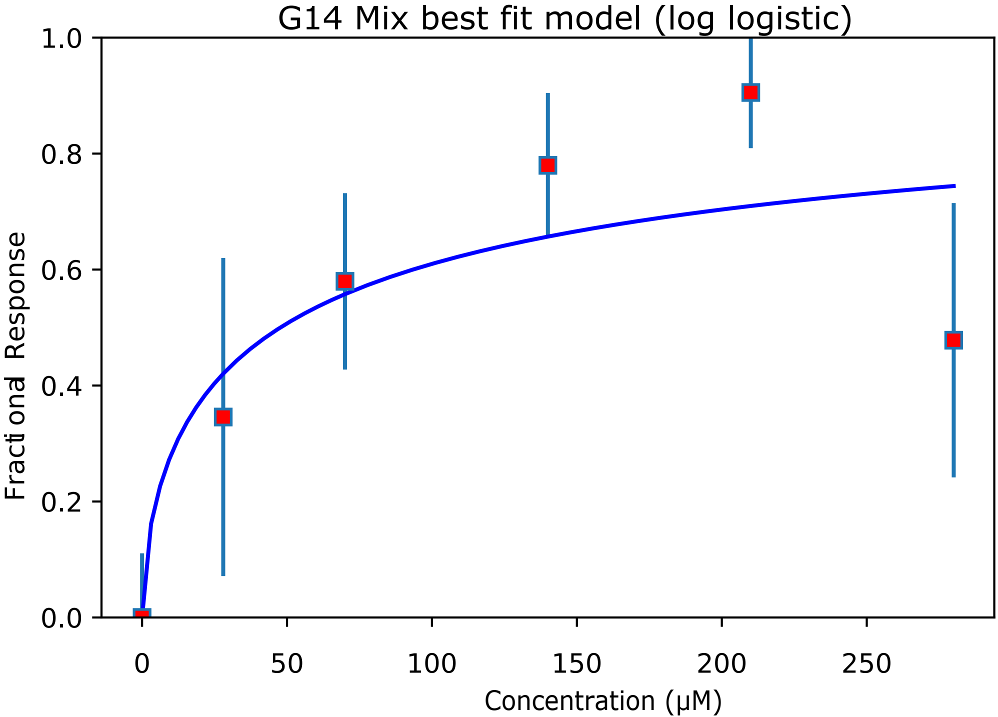

Figure S2: Concentration-response curves for each active chemical and mixture for cell viability in NHBE.

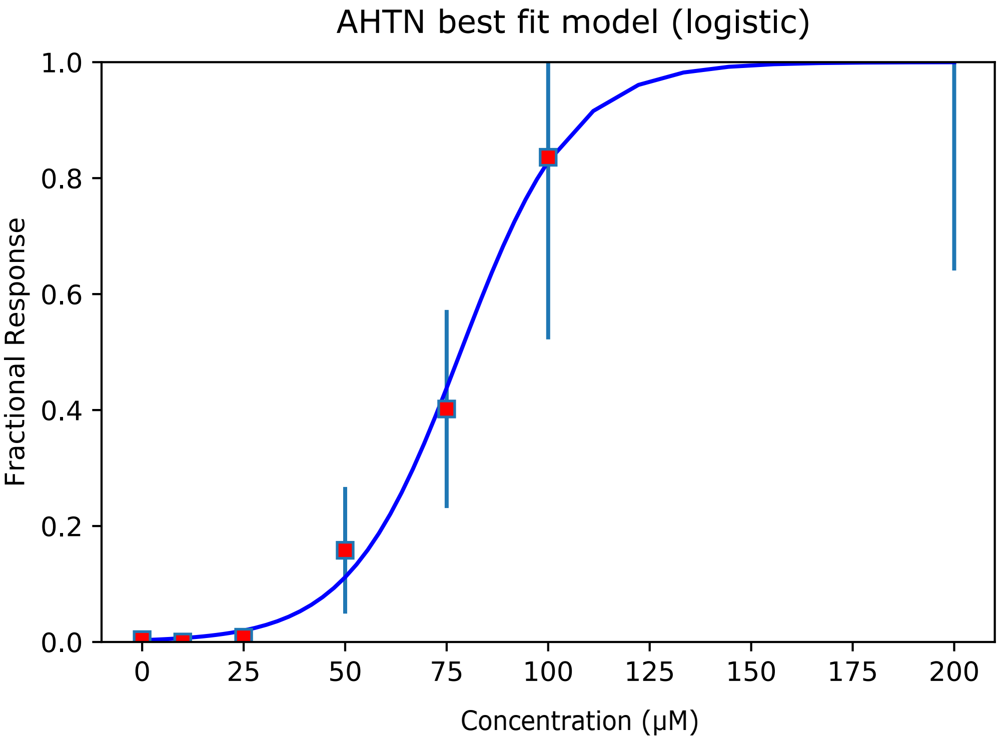
AHTN best fit model (logistic)
Fractional Response
Concentration (μM)
0.0
0.2
0.4
0.6
0.8
1.0
0
25
50
75
100
125
150
175
200

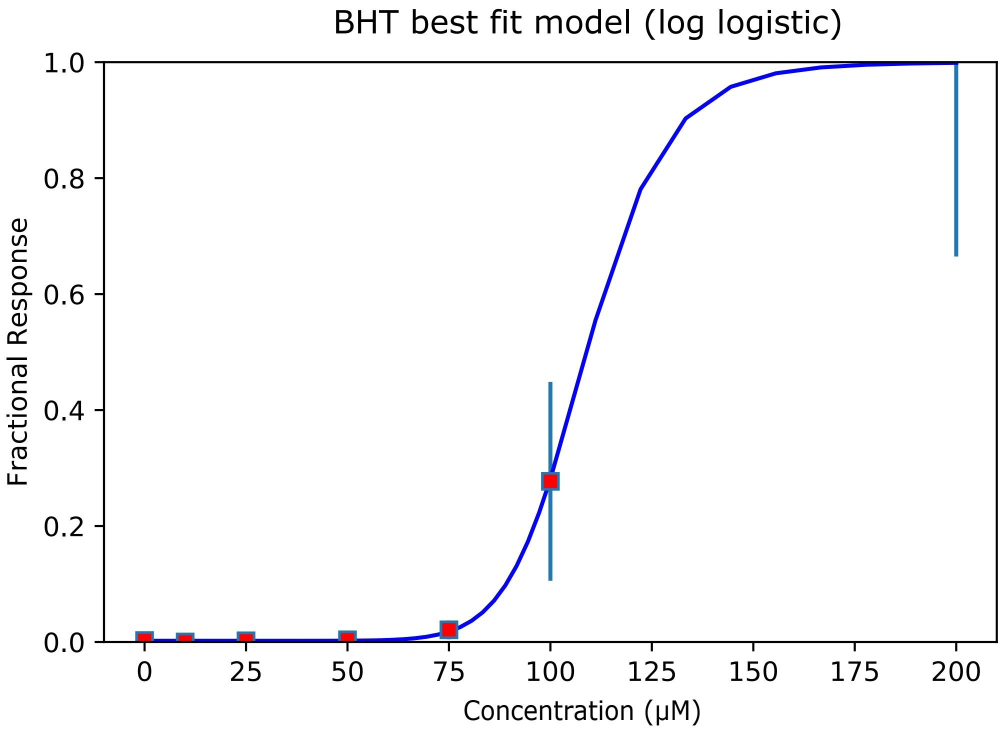

BHT best fit model (log logistic)
Fractional Response
1.0
0.8
0.6
0.4
0.2
0.0
0
25
50
75
100
125
150
175
200
Concentration (μM)

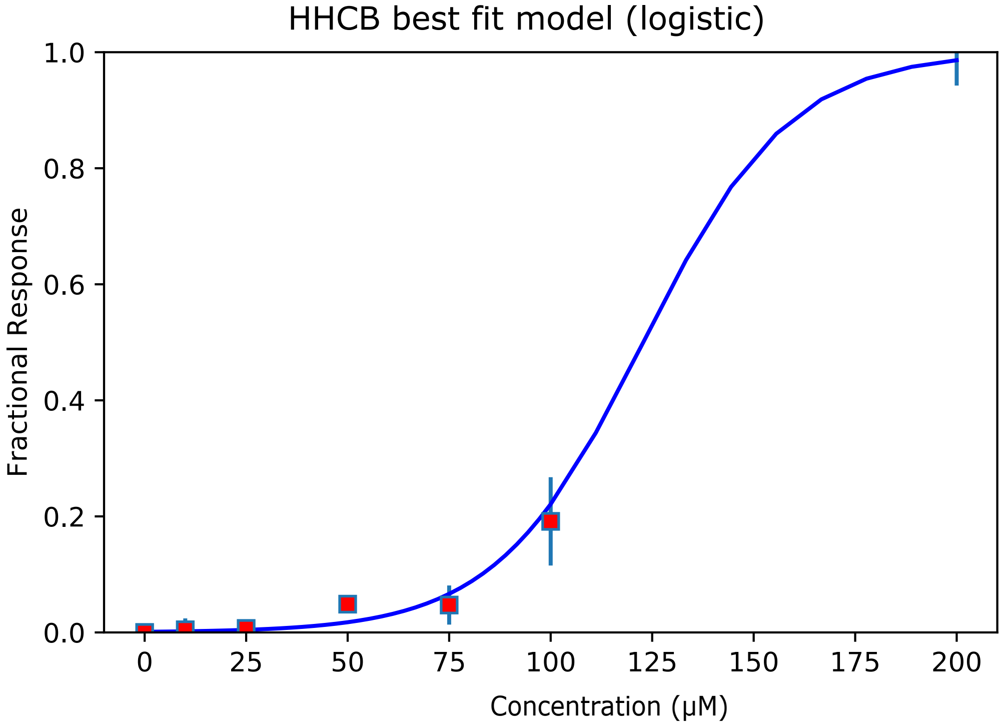
HHCB best fit model (logistic)
Fractional Response
1.0
0.8
0.6
0.4
0.2
0.0
0
25
50
75
100
125
150
175
200
Concentration (μM)

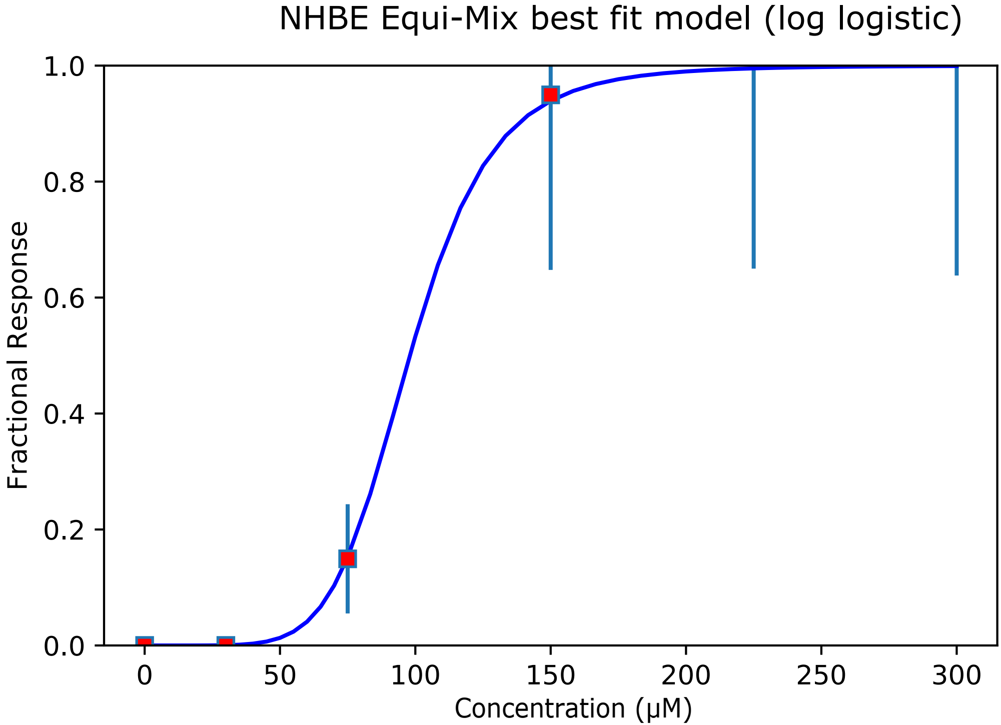

Figure S3: Concentration-response curves for each active chemical and mixture for cytotoxicity in NHBE.

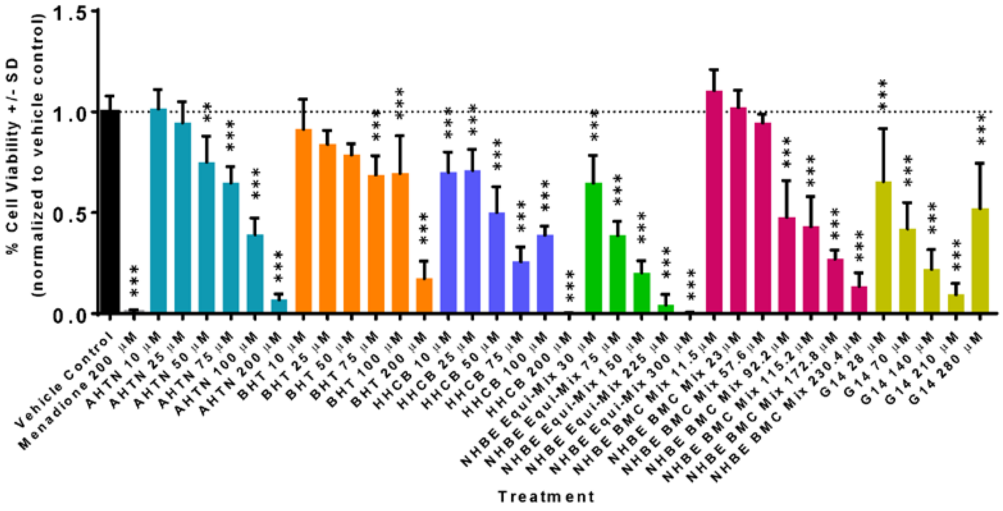

Figure S4.

Concentration response bar plots for cell viability in NHBE. Significantly different responses from control were analyzed using one-way ANOVA with Dunnett's mutliple comparison correction. * p < 0.05; ** p < 0.001; *** p < 0.0001

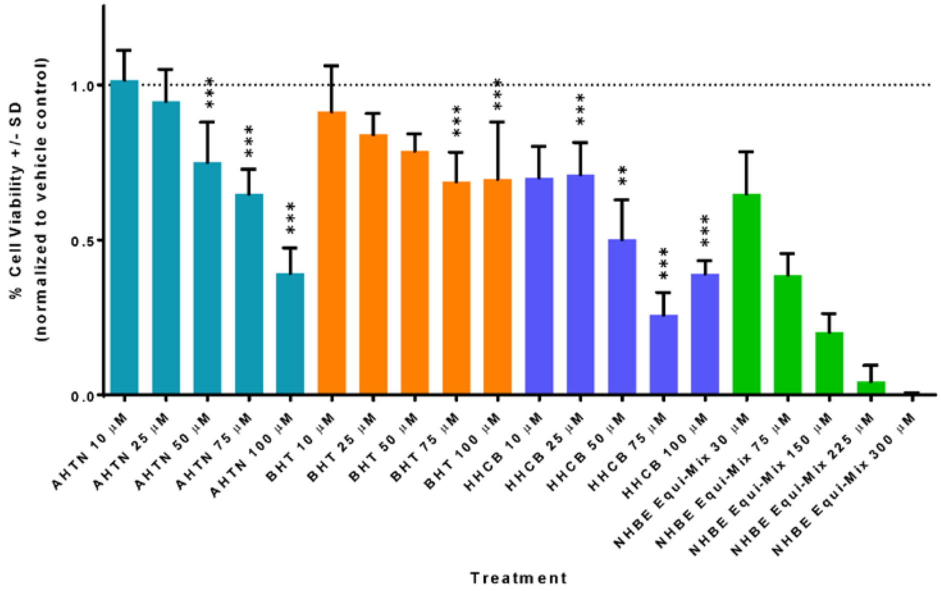

Figure S5.

Pair-wise comparison between NHBE Equi-Mix and individual components for cell viability. Significantly different responses from control were analyzed using Tukey's honest significant difference test. Chemicals significant different from control (Figure S4) were compared to their respective concentration in NHBE Equi-Mix. * p < 0.05; ** p < 0.001; *** p < 0.0001

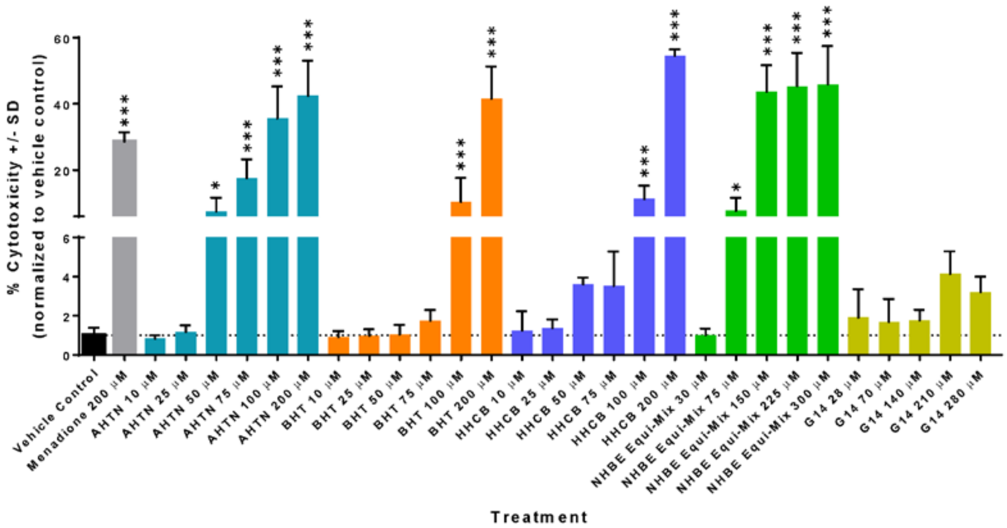

Figure S6.

Concentration response bar plots for cytotoxicity in NHBE. Significantly different responses from control were analyzed using one-way ANOVA with Dunnett's mutliple comparison correction. * $p < 0.05$; ** $p < 0.001$; *** $p < 0.0001$

Table S1. Description of each morphological endpoint screened in zebrafish

Endpoint ID	Endpoints in phenotype atlas	Description
MO24	---	Mortality at 24 hpf
DP24	---	Delayed developmental progression at 24 hpf
SM24	---	Spontaneous movement at 24 hpf
MORT	---	Mortality at 120 hpf
CRAN	eyes, jaw, snout	Malformed or missing eye, jaw, and/or snout at 120 hpf
AXIS	axis	Curved or bent axis at 120 hpf
EDEM	pericardial edema, yolk sad edema	Pericardial and/or yolk sac edema, heart and/or yolk sac malformation at 120 hpf
MUSC	circulatory system, somites, swim bladder	Lack of circulation, malformed or missing somites and/or swim bladder at 120 hpf
LTRK	trunk, caudal fin	Malformed lower trunk and/or caudal fin at 120 hpf
BRN_	brain, otic vesicle, pectoral fins	Malformed or necrotic brain, otic vesicle, or pectoral fin at 120 hpf
SKIN	pigment	Abnormal pigmentation at 120 hpf
NC__	notochord	Malformed notochord at 120 hpf
TCHR	---	Lack of response to touch at 120 hpf
any.effect	---	Incidence of any of the above morphological effects

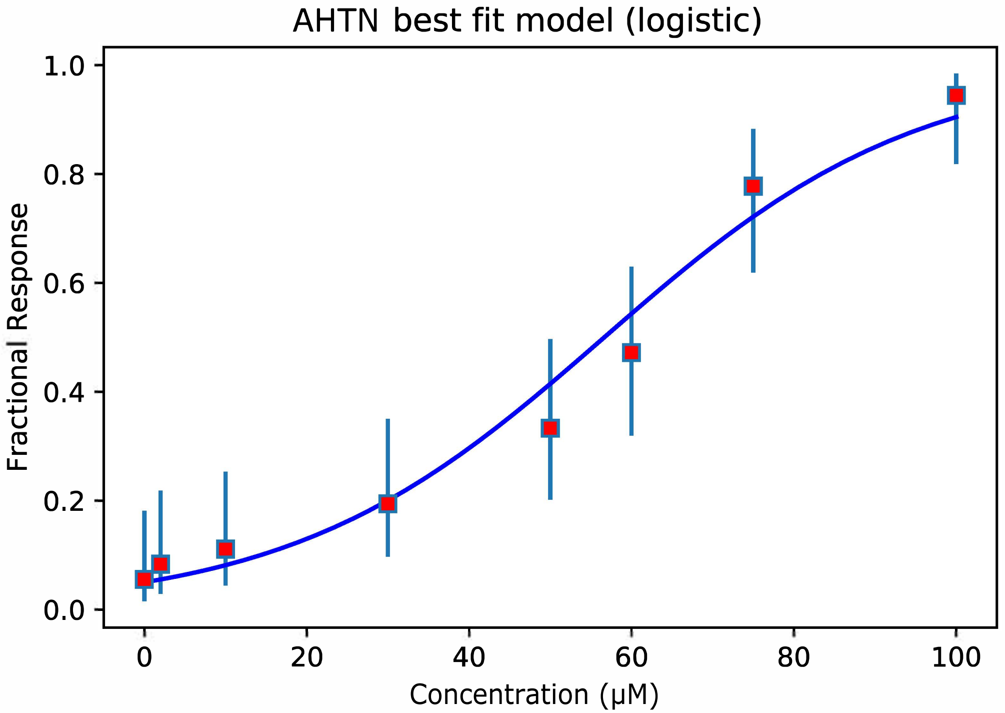
AHTN best fit model (logistic)
Fractional Response
Concentration (μM)
1.0
0.8
0.6
0.4
0.2
0.0
0
20
40
60
80
100

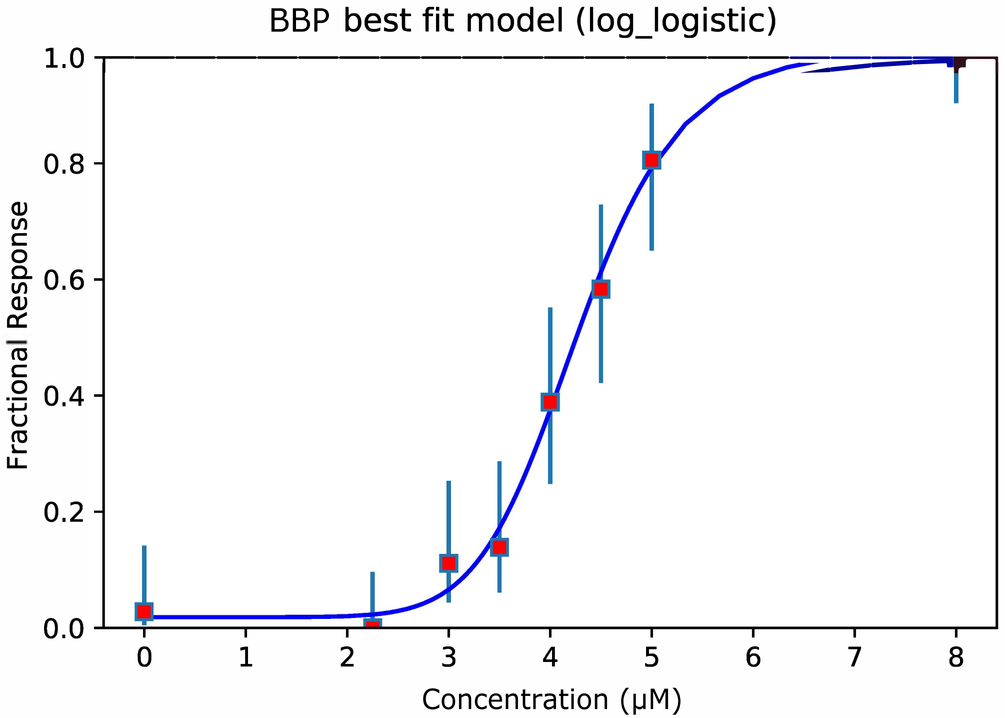
BBP best fit model (log_logistic)
Fractional Response
Concentration (μM)
0.0
0.2
0.4
0.6
0.8
1.0
0
1
2
3
4
5
6
7
8

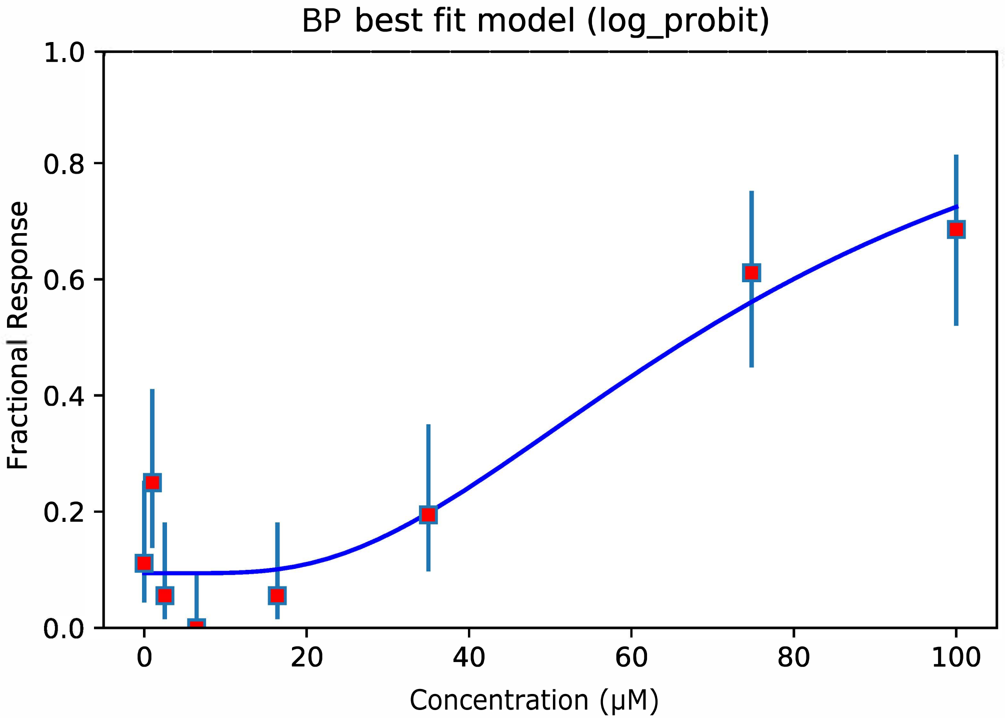
BP best fit model (log_probit)
1.0
0.8
0.6
0.4
0.2
0.0
Fractional Response
0
20
40
60
80
100
Concentration (μM)

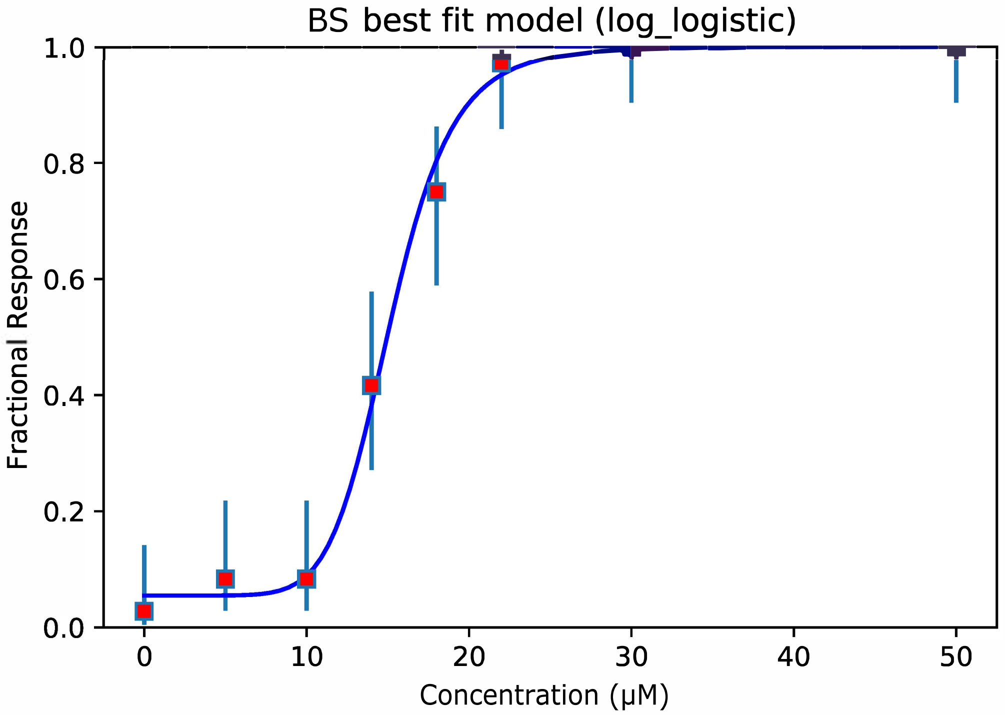

BS best fit model (log_logistic)
Fractional Response
Concentration (μM)
1.0
0.8
0.6
0.4
0.2
0.0
0
10
20
30
40
50

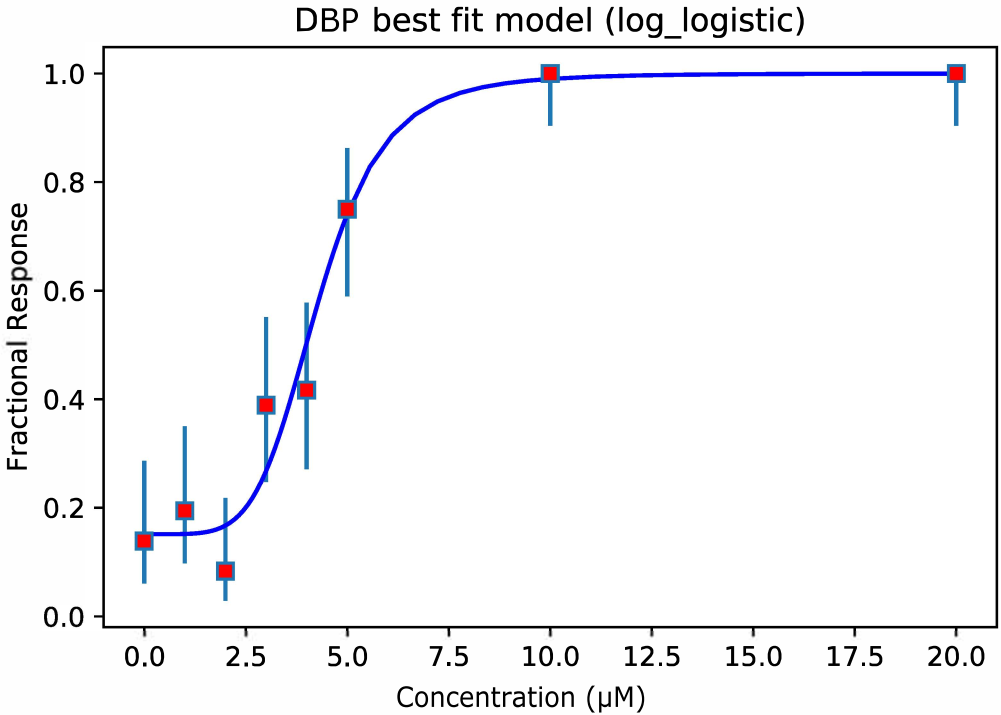

DBP best fit model (log_logistic)
Fractional Response
Concentration (μM)
0.0
0.2
0.4
0.6
0.8
1.0
0.0
2.5
5.0
7.5
10.0
12.5
15.0
17.5
20.0

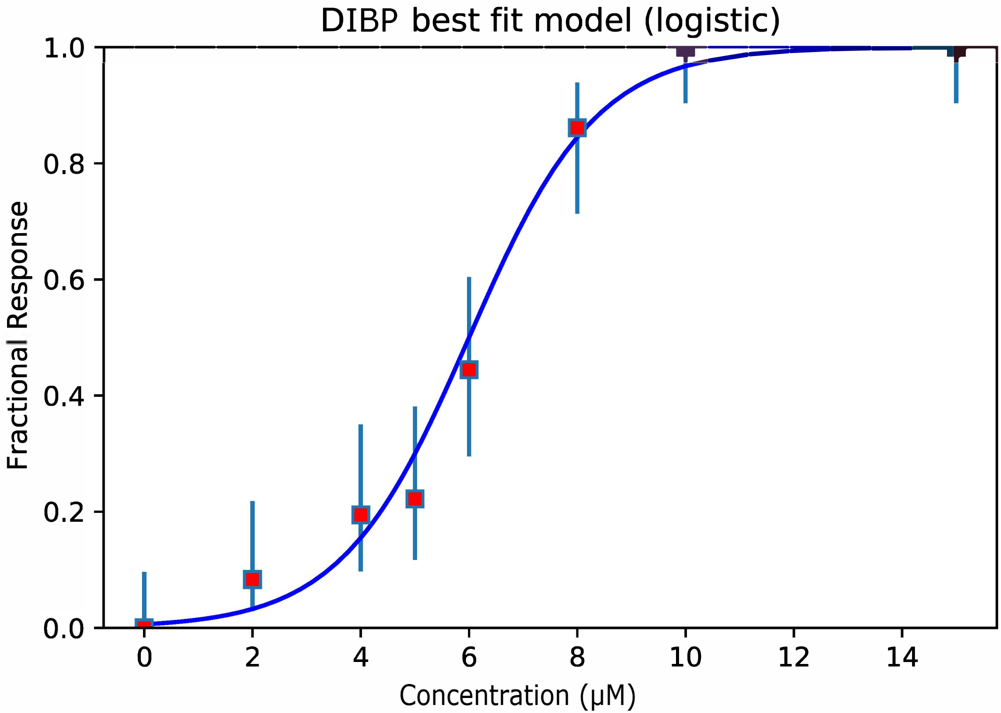

DIBP best fit model (logistic)
Fractional Response
Concentration (μM)
0.0
0.2
0.4
0.6
0.8
1.0
0
2
4
6
8
10
12
14

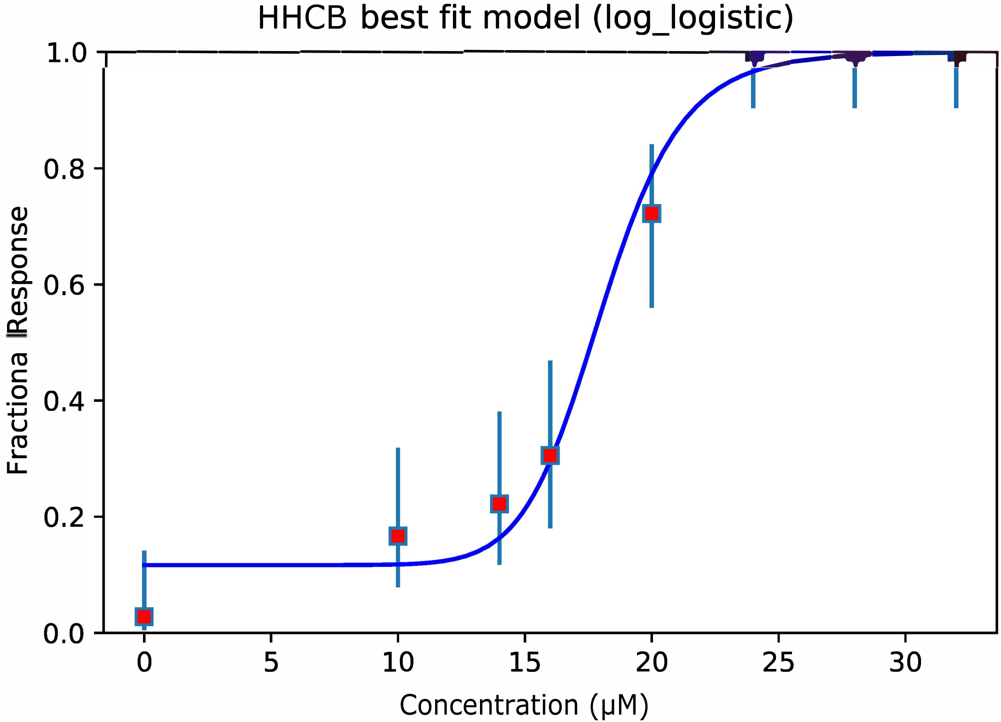

HHCB best fit model (log_logistic)
Fractional Response
Concentration (μM)
1.0
0.8
0.6
0.4
0.2
0.0
0
5
10
15
20
25
30

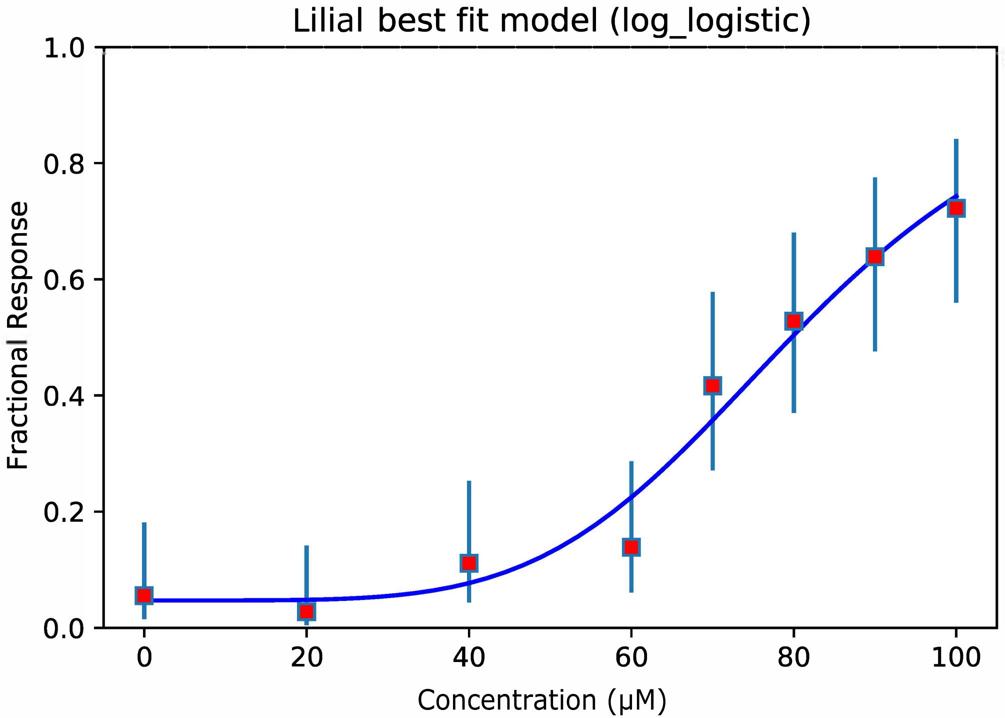

Lilial best fit model (log_logistic)
1.0
0.8
0.6
0.4
0.2
0.0
Fractional Response
0
20
40
60
80
100
Concentration (μM)

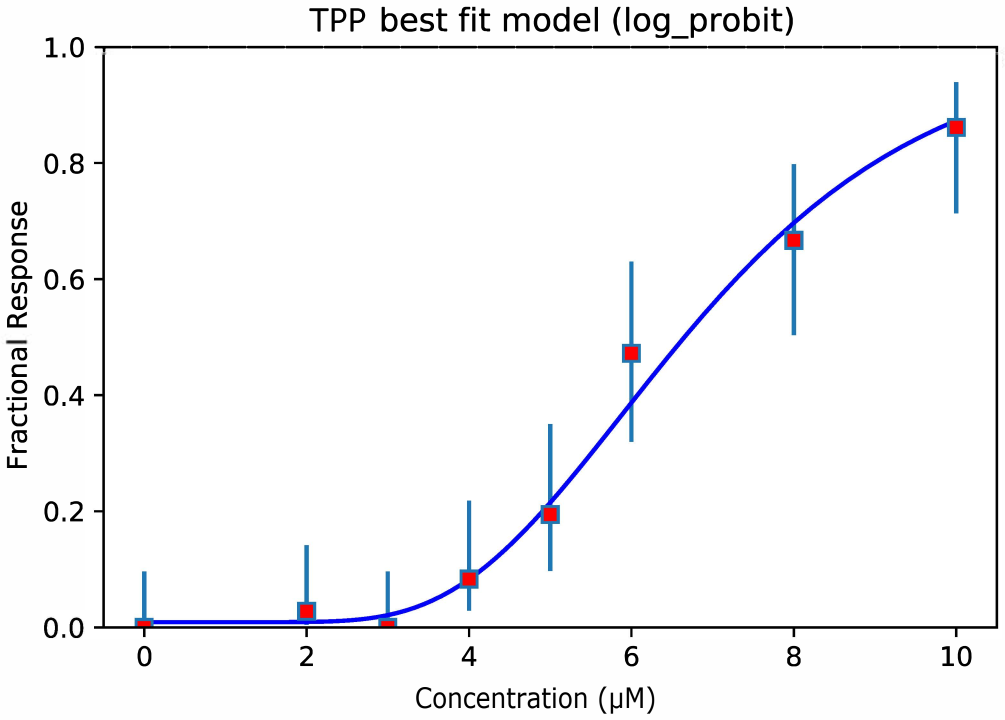

TPP best fit model (log_probit)
Fractional Response
0.0
0.2
0.4
0.6
0.8
1.0
0
2
4
6
8
10
Concentration (μM)

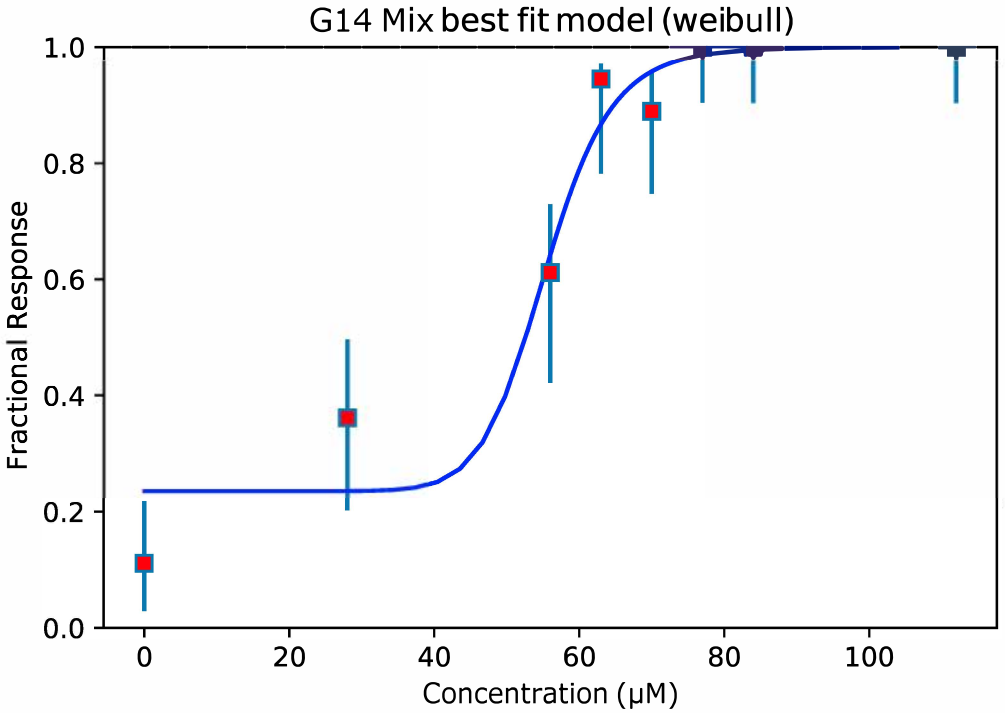
G14 Mix best fit model (weibull)
Fractional Response
Concentration (μM)
0.0
0.2
0.4
0.6
0.8
1.0
0
20
40
60
80
100

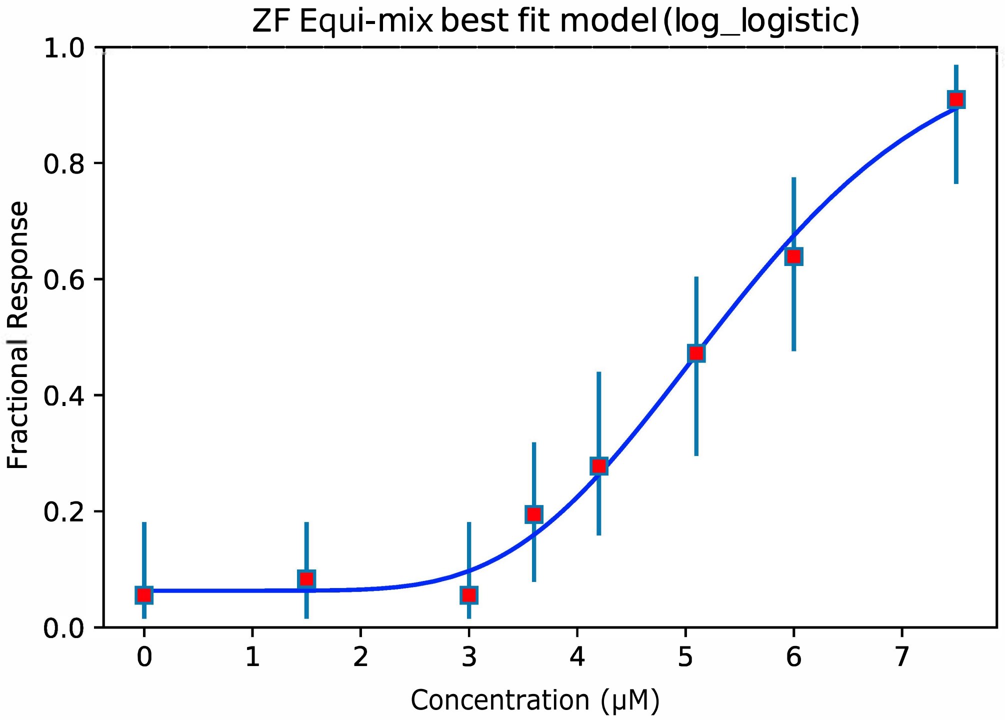

ZF Equi-mix best fit model (log_logistic)
Fractional Response
Concentration (μM)
0.0
0.2
0.4
0.6
0.8
1.0
0
1
2
3
4
5
6
7

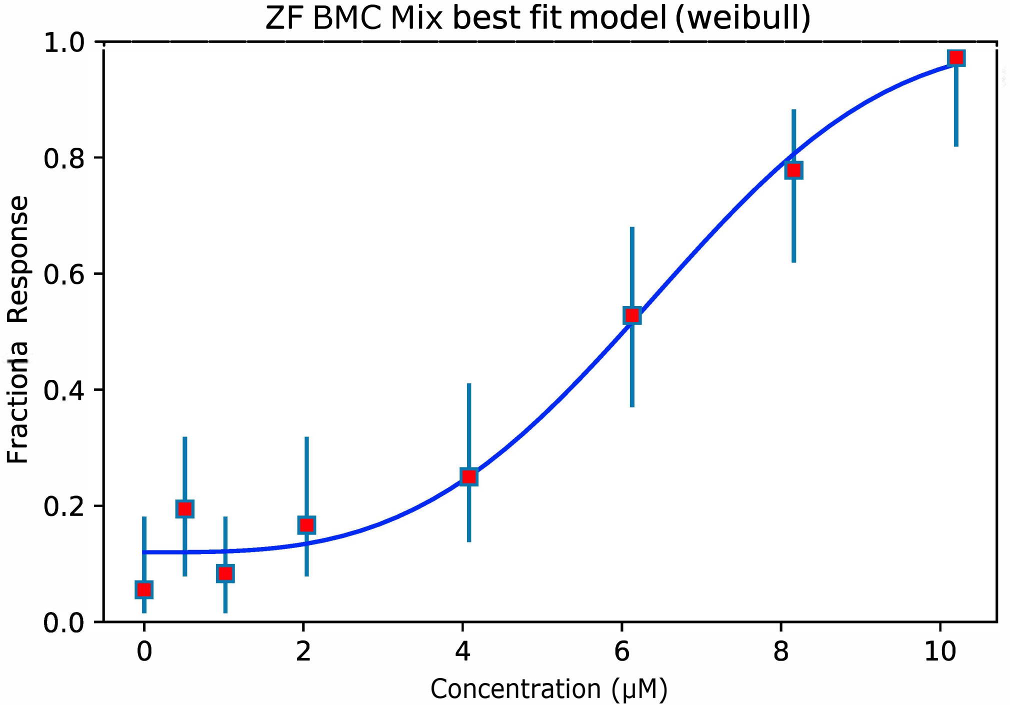

Figure S7: Concentration-response curves for each active chemical and mixture. Curve represents any effect in zebrafish morphology screening.

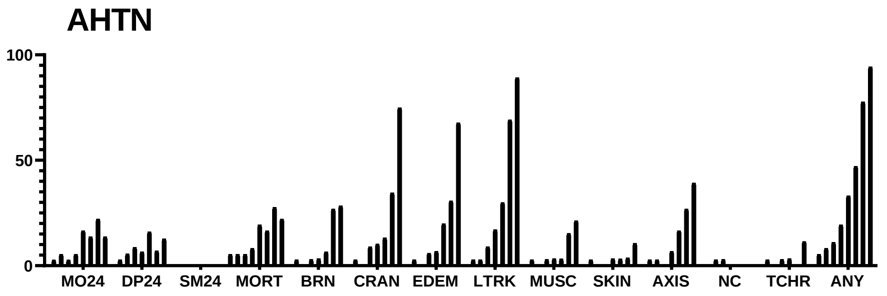
AHTN
100
50
0
MO24
DP24
SM24
MORT
BRN
CRAN
EDEM
LTRK
MUSC
SKIN
AXIS
NC
TCHR
ANY

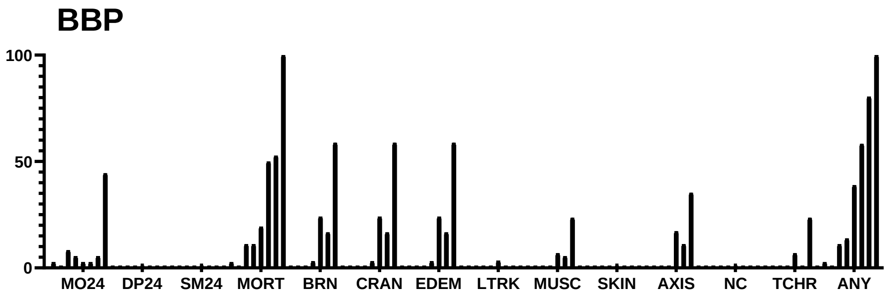
BBP
100
50
0
MO24
DP24
SM24
MORT
BRN
CRAN
EDEM
LTRK
MUSC
SKIN
AXIS
NC
TCHR
ANY

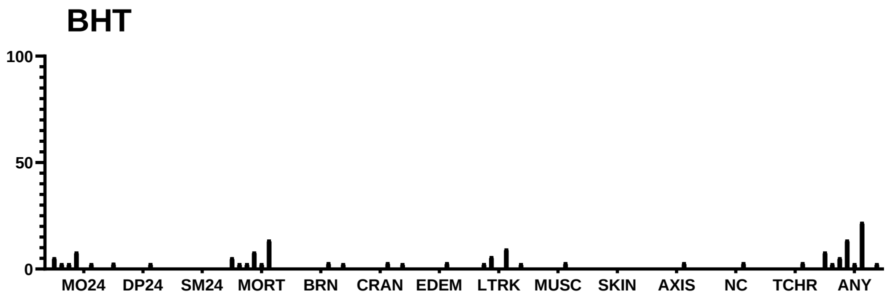
BHT
100
50
0
MO24
DP24
SM24
MORT
BRN
CRAN
EDEM
LTRK
MUSC
SKIN
AXIS
NC
TCHR
ANY

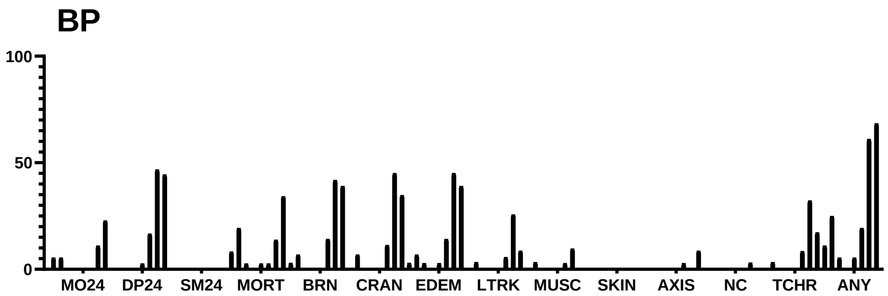
BP
100
50
0
MO24
DP24
SM24
MORT
BRN
CRAN
EDEM
LTRK
MUSC
SKIN
AXIS
NC
TCHR
ANY

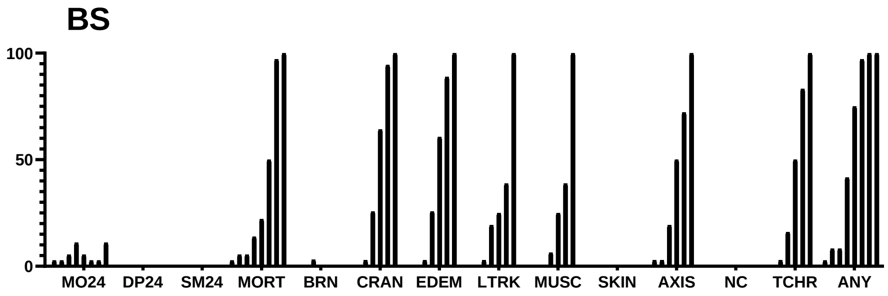
BS
100
50
0
MO24
DP24
SM24
MORT
BRN
CRAN
EDEM
LTRK
MUSC
SKIN
AXIS
NC
TCHR
ANY

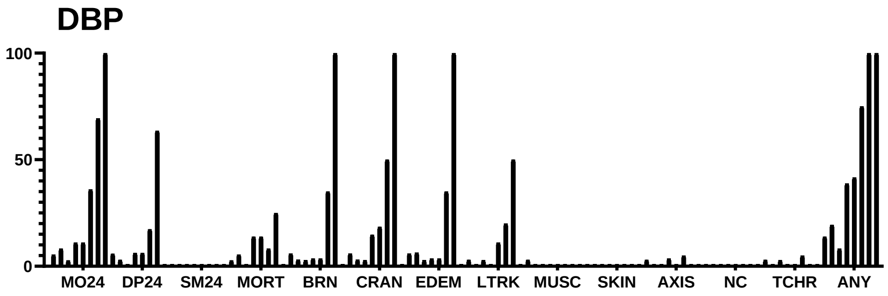
DBP
100
50
0
MO24
DP24
SM24
MORT
BRN
CRAN
EDEM
LTRK
MUSC
SKIN
AXIS
NC
TCHR
ANY

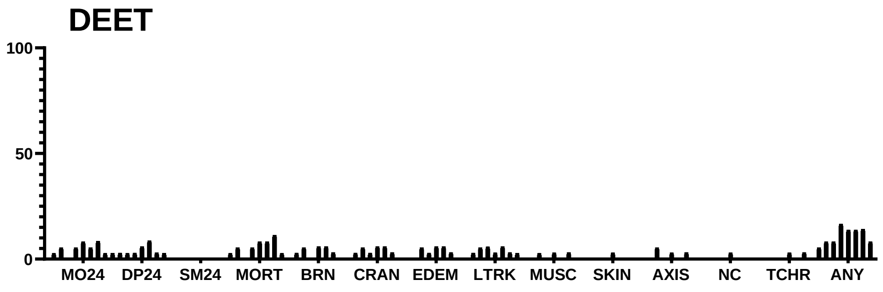
DEET
100
50
0
MO24
DP24
SM24
MORT
BRN
CRAN
EDEM
LTRK
MUSC
SKIN
AXIS
NC
TCHR
ANY

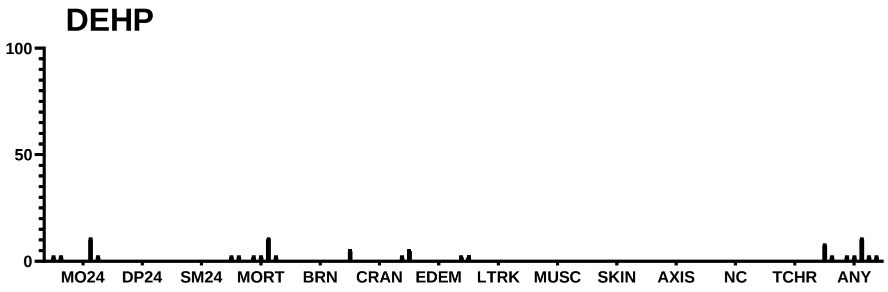
DEHP
100
50
0
MO24
DP24
SM24
MORT
BRN
CRAN
EDEM
LTRK
MUSC
SKIN
AXIS
NC
TCHR
ANY

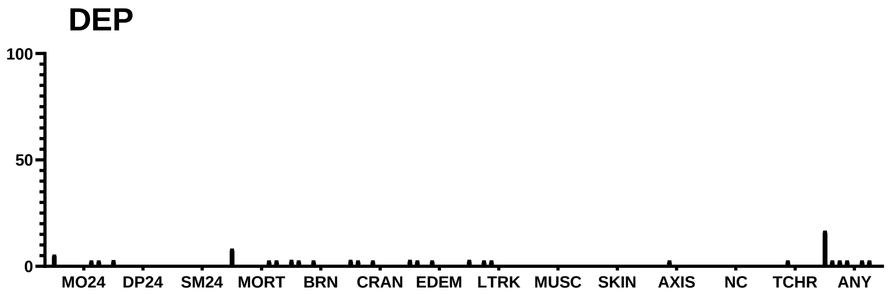
DEP
100
50
0
MO24
DP24
SM24
MORT
BRN
CRAN
EDEM
LTRK
MUSC
SKIN
AXIS
NC
TCHR
ANY

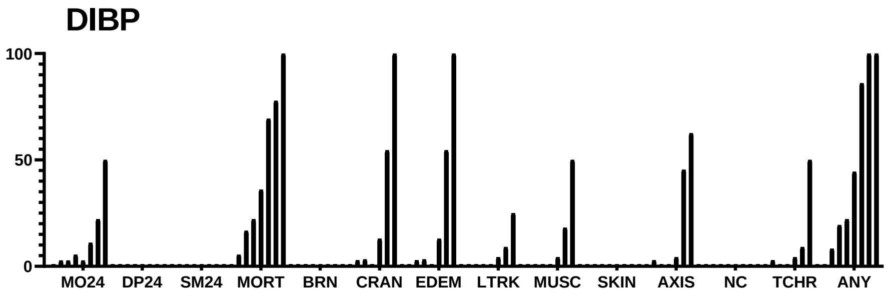
DIBP
100
50
0
MO24
DP24
SM24
MORT
BRN
CRAN
EDEM
LTRK
MUSC
SKIN
AXIS
NC
TCHR
ANY

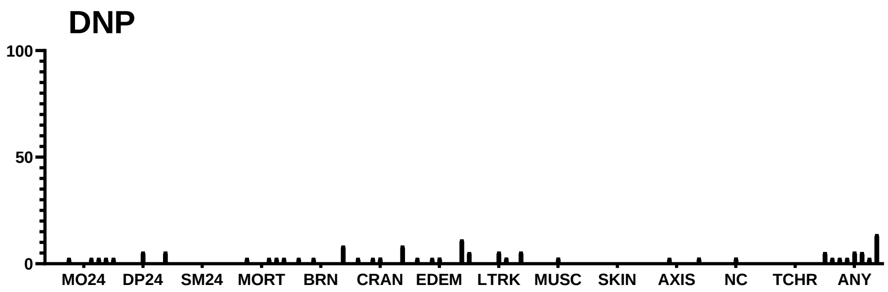
DNP
100
50
0
MO24
DP24
SM24
MORT
BRN
CRAN
EDEM
LTRK
MUSC
SKIN
AXIS
NC
TCHR
ANY

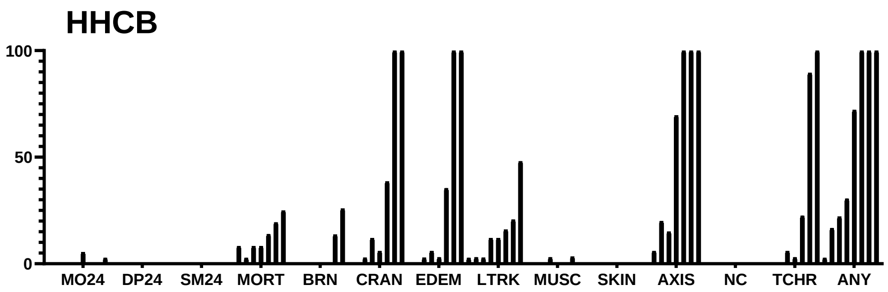
HHCB
100
50
0
MO24
DP24
SM24
MORT
BRN
CRAN
EDEM
LTRK
MUSC
SKIN
AXIS
NC
TCHR
ANY

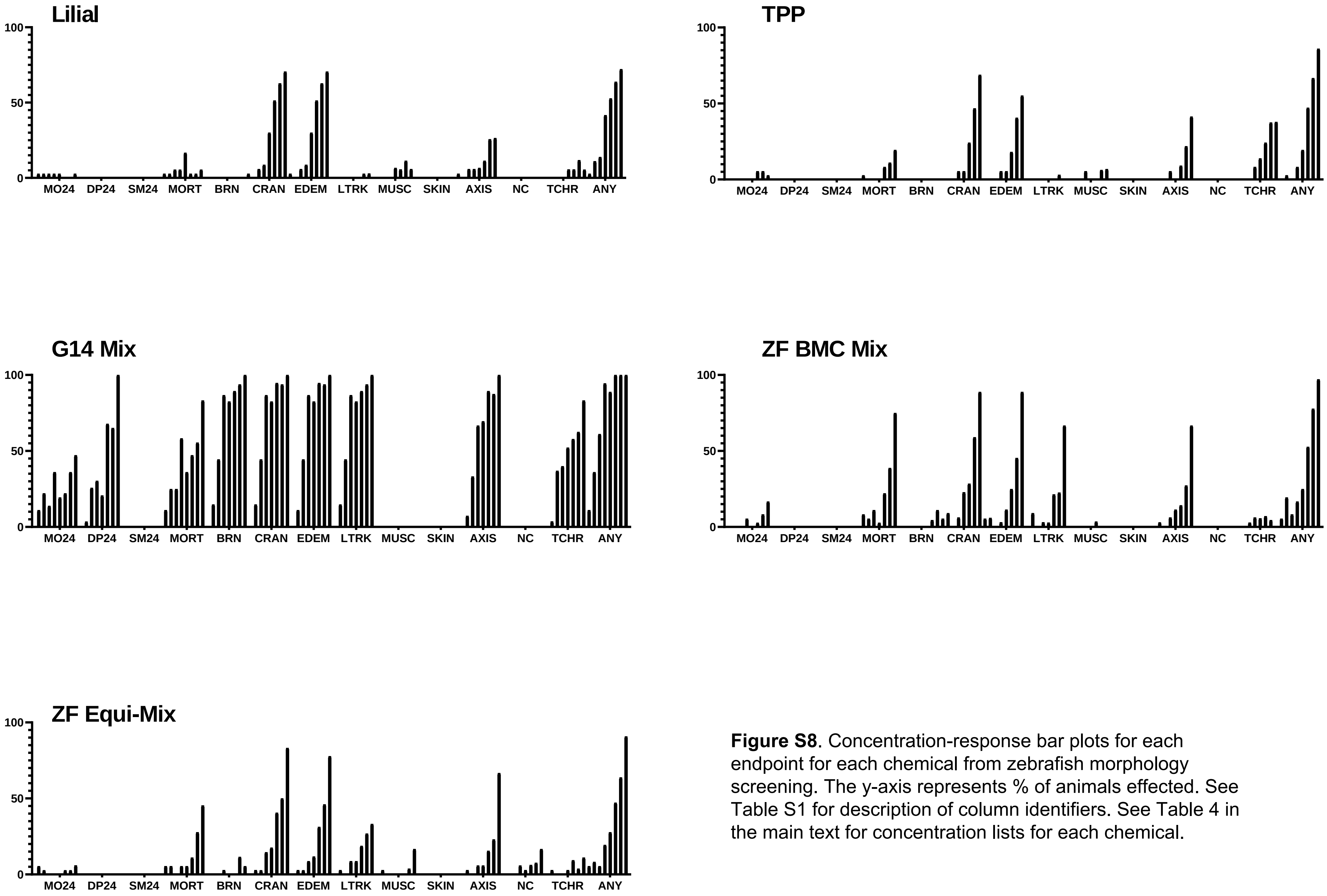

Figure S8. Concentration-response bar plots for each endpoint for each chemical from zebrafish morphology screening. The y-axis represents % of animals effected. See Table S1 for description of column identifiers. See Table 4 in the main text for concentration lists for each chemical.

Table S2: Percent incidence of each endpoint from morphology screening. Table shows data from three replicate plates (n=12 each) for each chemical. See Table S1 for description of column identifiers.

chemical.name	concentration (µM)	MO24	DP24	SM24	MORT	CRAN	AXIS	EDEM	MUSC	LTRK	BRN_	SKIN	NC__	TCHR	any.effect
AHTN	0	2.777778	0	0	5.5555556	0	0	0	0	0	0	0	0	0	5.56
AHTN	2	5.555556	2.941176	0	5.5555556	2.941176	2.941176	2.941176	2.941176	2.941176	2.941176	2.941176	0	2.941176	8.33
AHTN	10	2.777778	5.714286	0	5.5555556	0	2.941176	0	0	2.941176	0	0	2.941176	0	11.11
AHTN	30	5.555556	8.823529	0	8.3333333	9.090909	0	6.060606	3.030303	9.090909	3.030303	0	3.030303	3.030303	19.44
AHTN	50	16.66667	6.666667	0	19.444444	10.34483	6.896552	6.896552	3.448276	17.24138	3.448276	3.448276	0	3.448276	33.33
AHTN	60	13.88889	16.12903	0	16.666667	13.33333	16.66667	20	3.333333	30	6.666667	3.333333	0	0	47.22
AHTN	75	22.22222	7.142857	0	27.777778	34.61538	26.92308	30.76923	15.38462	69.23077	26.92308	3.846154	0	11.53846	77.78
AHTN	100	13.88889	12.90323	0	22.222222	75	39.28571	67.85714	21.42857	89.28571	28.57143	10.71429	0	0	94.44
BBP	0	2.78	0	0	2.78	0	0	0	0	0	0	0	0	0	2.78
BBP	2.25	0	0	0	0	0	0	0	0	0	0	0	0	0	0
BBP	3	8.33	0	0	11.11	0	0	0	0	0	0	0	0	0	11.11
BBP	3.5	5.56	0	0	11.11	3.12	0	3.12	0	0	0	0	0	0	13.89
BBP	4	2.78	0	0	19.44	24.14	17.24	24.14	6.9	3.45	0	0	0	6.9	38.89
BBP	4.5	2.78	0	0	50	16.67	11.11	16.67	5.56	0	0	0	0	0	58.33
BBP	5	5.56	0	0	52.78	58.82	35.29	58.82	23.53	0	0	0	0	23.53	80.56
BBP	8	44.44	0	0	100	0	0	0	0	0	0	0	0	0	100
BHT	0	5.555556	2.941176	0	5.5555556	0	0	0	0	0	0	0	0	0	8.33
BHT	1	2.777778	0	0	2.7777778	0	0	0	0	0	0	0	0	0	2.78
BHT	2.54	2.777778	0	0	2.7777778	0	0	0	0	2.857143	0	0	0	0	5.56
BHT	6.45	8.333333	0	0	8.3333333	0	0	0	0	6.060606	0	0	0	0	13.89
BHT	16.4	0	0	0	2.7777778	0	0	0	0	0	0	0	0	0	2.78
BHT	35	2.777778	2.857143	0	13.888889	3.225806	3.225806	3.225806	3.225806	9.677419	3.225806	0	3.225806	3.225806	22.22
BHT	74.8	0	0	0	0	0	0	0	0	0	0	0	0	0	0
BHT	100	0	0	0	0	2.777778	0	0	0	2.777778	2.777778	0	0	0	2.78
BP	0	5.555556	0	0	8.3333333	0	0	3.030303	0	0	3.030303	0	0	0	11.11
BP	1	5.555556	0	0	19.444444	6.896552	0	6.896552	3.448276	3.448276	6.896552	0	0	3.448276	25
BP	2.54	0	0	0	2.7777778	0	0	2.857143	0	0	0	0	0	0	5.56
BP	6.45	0	0	0	0	0	0	0	0	0	0	0	0	0	0
BP	16.4	0	2.777778	0	2.7777778	0	0	2.857143	0	0	0	0	0	0	5.56
BP	35	0	16.66667	0	2.7777778	11.42857	2.857143	14.28571	2.857143	5.714286	14.28571	0	0	8.571429	19.44
BP	74.8	11.11111	46.875	0	13.888889	45.16129	0	45.16129	9.677419	25.80645	41.93548	0	3.225806	32.25806	61.11
BP	100	22.85714	44.44444	0	34.285714	34.78261	8.695652	39.13043	0	8.695652	39.13043	0	0	17.3913	68.57
BS	0	2.78	0	0	2.78	0	0	0	0	0	0	0	0	0	2.78
BS	5	2.78	0	0	5.56	0	2.94	0	0	0	0	0	0	0	8.33
BS	10	5.56	0	0	5.56	2.94	2.94	2.94	0	2.94	0	0	0	2.94	8.33
BS	14	11.11	0	0	13.89	25.81	19.35	25.81	6.45	19.35	3.23	0	0	16.13	41.67
BS	18	5.56	0	0	22.22	64.29	50	60.71	25	25	0	0	0	50	75
BS	22	2.78	0	0	50	94.44	72.22	88.89	38.89	38.89	0	0	0	83.33	97.22
BS	30	2.78	0	0	97.22	100	100	100	100	100	0	0	0	100	100
BS	50	11.11	0	0	100	NA	NA	NA	NA	NA	NA	NA	NA	NA	100
DBP	0	5.555556	5.882353	0	2.7777778	6.060606	3.030303	6.060606	3.030303	3.030303	6.060606	0	0	3.030303	13.89
DBP	1	8.333333	3.030303	0	5.5555556	3.225806	0	6.451613	0	0	3.225806	0	0	0	19.44
DBP	2	2.777778	0	0	0	2.857143	0	2.857143	0	2.857143	2.857143	0	0	2.857143	8.33

DBP	3	11.11111	6.25	0	13.888889	14.81481	3.703704	3.703704	0	0	3.703704	0	0	0	38.89
DBP	4	11.11111	6.25	0	13.888889	18.51852	0	3.703704	0	11.11111	3.703704	0	0	0	41.67
DBP	5	36.11111	17.3913	0	8.3333333	50	5	35	0	20	35	0	0	5	75
DBP	10	69.44444	63.63636	0	25	100	0	100	0	50	100	0	0	0	100
DBP	20	100	0	0	0	0	0	0	0	0	0	0	0	0	100
DEET	0	2.777778	2.857143	0	2.7777778	0	0	0	0	0	0	0	0	0	5.56
DEET	1	5.555556	2.941176	0	5.5555556	2.941176	0	0	0	2.941176	2.941176	0	0	0	8.33
DEET	2.54	0	2.777778	0	0	5.555556	5.555556	5.555556	2.777778	5.555556	5.555556	0	0	0	8.33
DEET	6.45	5.555556	2.941176	0	5.5555556	2.941176	0	2.941176	0	5.882353	0	0	0	0	16.67
DEET	16.4	8.333333	6.060606	0	8.3333333	6.060606	3.030303	6.060606	3.030303	3.030303	6.060606	3.030303	3.030303	3.030303	13.89
DEET	35	5.555556	8.823529	0	8.3333333	6.060606	0	6.060606	0	6.060606	6.060606	0	0	0	13.89
DEET	74.8	8.571429	3.125	0	11.428571	3.225806	3.225806	3.225806	3.225806	3.225806	3.225806	0	0	3.225806	14.29
DEET	100	2.777778	2.857143	0	2.7777778	0	0	0	0	2.857143	0	0	0	0	8.33
DEHP	0	2.78	0	0	2.78	5.71	0	5.71	0	2.86	0	0	0	0	8.33
DEHP	1	2.78	0	0	2.78	0	0	0	0	0	0	0	0	0	2.78
DEHP	3	0	0	0	0	0	0	0	0	0	0	0	0	0	0
DEHP	5	0	0	0	2.78	0	0	0	0	0	0	0	0	0	2.78
DEHP	10	0	0	0	2.78	0	0	0	0	0	0	0	0	0	2.78
DEHP	20	11.11	0	0	11.11	0	0	0	0	0	0	0	0	0	11.11
DEHP	40	2.78	0	0	2.78	0	0	0	0	0	0	0	0	0	2.78
DEHP	80	0	0	0	0	2.78	0	2.78	0	0	0	0	0	0	2.78
DEP	0	5.555556	2.941176	0	8.3333333	3.030303	0	3.030303	0	3.030303	3.030303	0	0	0	16.67
DEP	1	0	0	0	0	2.777778	0	2.777778	0	0	2.777778	0	0	0	2.78
DEP	2.54	0	0	0	0	0	0	0	0	2.777778	0	0	0	0	2.78
DEP	6.45	0	0	0	0	2.777778	2.777778	2.777778	0	2.777778	2.777778	0	0	2.777778	2.78
DEP	16.4	0	0	0	0	0	0	0	0	0	0	0	0	0	0
DEP	35	2.777778	0	0	2.7777778	0	0	0	0	0	0	0	0	0	2.78
DEP	74.8	2.777778	0	0	2.7777778	0	0	0	0	0	0	0	0	0	2.78
DEP	100	0	0	0	0	0	0	0	0	0	0	0	0	0	0
DIBP	0	0	0	0	0	0	0	0	0	0	0	0	0	0	0
DIBP	2	2.78	0	0	5.56	2.94	2.94	2.94	0	0	0	0	0	2.94	8.33
DIBP	4	2.78	0	0	16.67	3.33	0	3.33	0	0	0	0	0	0	19.44
DIBP	5	5.56	0	0	22.22	0	0	0	0	0	0	0	0	0	22.22
DIBP	6	2.78	0	0	36.11	13.04	4.35	13.04	4.35	4.35	0	0	0	4.35	44.44
DIBP	8	11.11	0	0	69.44	54.55	45.45	54.55	18.18	9.09	0	0	0	9.09	86.11
DIBP	10	22.22	0	0	77.78	100	62.5	100	50	25	0	0	0	50	100
DIBP	15	50	0	0	100	NA	NA	NA	NA	NA	NA	NA	NA	NA	100
DNP	0	0	2.777778	0	0	0	0	0	0	5.555556	0	0	0	0	5.56
DNP	2	0	0	0	0	2.777778	0	2.777778	0	0	2.777778	0	0	0	2.78
DNP	10	2.777778	0	0	2.7777778	0	0	0	0	0	0	0	0	0	2.78
DNP	30	0	0	0	0	2.777778	2.777778	2.777778	0	0	2.777778	0	0	0	2.78
DNP	50	0	5.714286	0	0	2.857143	0	2.857143	2.857143	5.714286	0	0	2.857143	0	5.71
DNP	70	2.777778	0	0	2.7777778	0	0	0	0	2.857143	0	0	0	0	5.56
DNP	80	2.777778	0	0	2.7777778	0	0	0	0	0	0	0	0	0	2.78

DNP	100	2.777778	5.714286	0	2.7777778	8.571429	2.857143	11.42857	0	5.714286	8.571429	0	0	0	13.89
G14 Mix	0	11.11111	0	0	11.111111	0	0	0	0	0	0	0	0	0	11.11
G14 Mix	28	22.22222	3.571429	0	25	14.81481	7.407407	11.11111	0	14.81481	14.81481	0	0	3.703704	36.11
G14 Mix	56	13.88889	25.80645	0	25	44.44444	33.33333	44.44444	0	44.44444	44.44444	0	0	37.03704	61.11
G14 Mix	63	36.11111	30.43478	0	58.333333	86.66667	66.66667	86.66667	0	86.66667	86.66667	0	0	40	94.44
G14 Mix	70	19.44444	20.68966	0	36.111111	82.6087	69.56522	82.6087	0	82.6087	82.6087	0	0	52.17391	88.89
G14 Mix	77	22.22222	67.85714	0	47.222222	94.73684	89.47368	94.73684	0	89.47368	89.47368	0	0	57.89474	100
G14 Mix	84	36.11111	65.21739	0	55.555556	93.75	87.5	93.75	0	93.75	93.75	0	0	62.5	100
G14 Mix	112	47.22222	100	0	83.333333	100	100	100	0	100	100	0	0	83.33333	100
HHCB	0	0	0	0	0	0	0	0	0	2.78	0	0	0	0	2.78
HHCB	10	0	0	0	8.33	0	6.06	0	0	3.03	0	0	0	0	16.67
HHCB	14	0	0	0	2.78	2.86	20	2.86	0	2.86	0	0	0	0	22.22
HHCB	16	0	0	0	8.33	12.12	15.15	6.06	3.03	12.12	0	0	0	6.06	30.56
HHCB	20	5.56	0	0	8.33	6.06	69.7	3.03	0	12.12	0	0	0	3.03	72.22
HHCB	24	0	0	0	13.89	38.71	100	35.48	0	16.13	0	0	0	22.58	100
HHCB	28	0	0	0	19.44	100	100	100	3.45	20.69	13.79	0	0	89.66	100
HHCB	32	2.78	0	0	25	100	100	100	0	48.15	25.93	0	0	100	100
Lilial	0	2.78	0	0	2.78	2.86	2.86	2.86	0	0	0	0	0	0	5.56
Lilial	20	2.78	0	0	2.78	0	0	0	0	0	0	0	0	0	2.78
Lilial	40	2.78	0	0	5.56	5.88	5.88	5.88	0	0	0	0	0	0	11.11
Lilial	60	2.78	0	0	5.56	8.82	5.88	8.82	0	0	0	0	0	0	13.89
Lilial	70	2.78	0	0	16.67	30	6.67	30	6.67	0	0	0	0	0	41.67
Lilial	80	0	0	0	2.78	51.43	11.43	51.43	5.71	0	0	0	0	5.71	52.78
Lilial	90	0	0	0	2.78	62.86	25.71	62.86	11.43	2.86	0	0	0	5.71	63.89
Lilial	100	2.78	0	0	5.56	70.59	26.47	70.59	5.88	2.94	0	0	0	11.76	72.22
TPP	0	0	0	0	0	0	0	0	0	0	0	0	0	0	0
TPP	2	0	0	0	2.78	0	0	0	0	0	0	0	0	0	2.78
TPP	3	0	0	0	0	0	0	0	0	0	0	0	0	0	0
TPP	4	0	0	0	0	5.56	5.56	5.56	5.56	0	0	0	0	8.33	8.33
TPP	5	0	0	0	0	5.56	0	5.56	0	0	0	0	0	13.89	19.44
TPP	6	5.56	0	0	8.33	24.24	9.09	18.18	0	0	0	0	0	24.24	47.22
TPP	8	5.56	0	0	11.11	46.88	21.88	40.62	6.25	3.12	0	0	0	37.5	66.67
TPP	10	2.78	0	0	19.44	68.97	41.38	55.17	6.9	0	0	0	0	37.93	86.11
ZF BMC Mix	0	0	0	0	0	5.56	0	5.56	0	0	0	0	0	0	5.56
ZF BMC Mix	0.511	0	0	0	8.33	9.09	3.03	6.06	0	9.09	0	0	0	0	19.44
ZF BMC Mix	1.02	0	0	0	5.56	0	0	0	0	0	0	0	0	2.94	8.33
ZF BMC Mix	2.041	5.56	0	0	11.11	6.25	6.25	3.12	0	3.12	0	0	0	6.25	16.67
ZF BMC Mix	4.018	0	0	0	2.78	22.86	11.43	11.43	0	2.86	0	0	0	5.71	25
ZF BMC Mix	6.13	2.78	0	0	22.22	28.57	14.29	25	3.57	21.43	0	0	0	7.14	52.78
ZF BMC Mix	8.16	8.33	0	0	38.89	59.09	27.27	45.45	0	22.73	4.55	0	0	4.55	77.78
ZF BMC Mix	10.2	16.67	0	0	75	88.89	66.67	88.89	0	66.67	11.11	0	0	0	97.22
ZF Equi-mix	0	5.56	0	0	5.56	0	0	0	0	0	0	0	0	0	5.56
ZF Equi-mix	1.5	2.78	0	0	5.56	2.94	2.94	2.94	2.94	2.94	0	0	0	2.94	8.33
ZF Equi-mix	3	0	0	0	0	2.78	0	2.78	0	0	0	0	0	0	5.56

ZF Equi-mix	3.6	0	0	0	5.56	14.71	5.88	8.82	0	8.82	2.94	0	5.88	0	19.44
ZF Equi-mix	4.2	0	0	0	5.56	17.65	5.88	11.76	0	8.82	0	0	2.94	2.94	27.78
ZF Equi-mix	5.1	2.78	0	0	11.11	40.62	15.62	31.25	0	18.75	0	0	6.25	9.38	47.22
ZF Equi-mix	6	2.78	0	0	27.78	50	23.08	46.15	3.85	26.92	11.54	0	7.69	3.85	63.89
ZF Equi-mix	7.5	6.06	0	0	45.45	83.33	66.67	77.78	16.67	33.33	5.56	0	16.67	11.11	90.91

Table S3: Percent cell viability and cytotoxicity in NHBE. Table shows data from two replicate plates (n=6 each) for each chemical. X = not tested.

Treatment Group	Concentration (µM)	% Cytotoxicity (normalized to vehicle control)	SD	% Cell viability (normalized to vehicle control)	SD
BBP	10	84.93464052	13.47355	89.9803728	39.91846
BBP	25	93.03921569	45.98058	90.9794952	45.37218
BBP	50	81.37254902	43.93242	105.565117	14.63564
BBP	75	99.70588235	47.84519	106.855072	9.574055
BBP	100	82.48366013	38.86088	100.321745	9.444511
BEHP	10	119.0147059	92.27305	103.610893	8.047967
BEHP	25	65.5	39.05772	112.360227	9.016499
BEHP	50	77.26470588	73.61252	119.521371	11.88143
BEHP	75	50.29411765	23.05114	114.097602	8.348966
BEHP	100	65.20588235	34.57175	99.7306459	19.54669
benzophenone	10	76.66666667	33.83066	82.9328401	40.74113
benzophenone	25	88.9869281	14.01949	101.286086	6.305546
benzophenone	50	73.9869281	26.75897	113.526937	3.742786
benzophenone	75	108.8562092	84.89643	90.6760432	44.66076
benzophenone	100	76.07843137	6.820599	103.184381	13.61852
benzyl salicylate	10	62.61437908	5.859651	110.463106	6.054533
benzyl salicylate	25	70.65359477	26.33609	107.107725	9.469752
benzyl salicylate	50	63.23529412	10.65682	90.4030113	44.47676
benzyl salicylate	75	103.1699346	60.8678	72.2764167	56.80649
benzyl salicylate	100	77.18954248	12.79638	104.805201	10.28514
BHT	10	89.5021645	31.80139	91.2866605	14.91296
BHT	25	97.8219697	32.91195	83.9832077	6.767774
BHT	50	104.586039	49.01575	78.6898805	5.51769
BHT	75	175.1352814	54.24609	68.7577369	9.50328
BHT	100	1218.295455	644.672	69.5670553	18.4819
BHT	200	4164.109848	965.0683	17.3965904	8.595863
DEET	10	84.83660131	20.06091	105.285534	15.51583
DEET	25	69.01960784	9.460657	109.840339	16.25336
DEET	50	69.21568627	25.18978	103.464348	13.2821
DEET	75	77.61437908	21.63463	109.49146	9.077438
DEET	100	85.71895425	17.99373	100.866135	12.08429
DEP	10	72.05882353	16.87539	105.984607	9.101288
DEP	25	74	18.68778	108.833245	13.61769
DEP	50	57.19117647	19.30869	110.03181	16.33637
DEP	75	66.85294118	19.07888	110.063558	8.788537
DEP	100	94.30882353	45.72756	105.641017	21.66387
DiBP	10	36.34090909	36.89292	121.00094	10.53565
DiBP	25	52.0530303	58.01729	110.099155	9.889056
DiBP	50	51.94848485	63.59185	97.9606036	9.919028
DiBP	75	43.36212121	57.44561	104.918257	4.678393
DiBP	100	65.12121212	79.63776	90.9451539	17.78272
DiBP	200	184.1257576	192.7245	72.3858922	15.36599
DnBP	10	70.64705882	15.42253	109.610767	19.20243
DnBP	25	119.7058824	36.9975	111.557907	14.00782
DnBP	50	104.8235294	53.0042	104.391442	12.75152
DnBP	75	100.5441176	42.60428	111.471932	2.972936
DnBP	100	69.91176471	21.45818	106.104978	7.793492

DnNP	10	109.3409091	35.66491	118.546957	7.514615
DnNP	25	127.8636364	67.94964	110.913996	13.08799
DnNP	50	83.54545455	33.8554	116.973114	10.65444
DnNP	75	91.31818182	34.23272	111.826255	5.638591
DnNP	100	143.2045455	31.9265	106.554618	10.60331
DnNP	200	125.8409091	25.77733	107.436102	15.04415
G14	28	191.1661255	145.0558	65.4303339	26.29084
G14	70	168.655303	117.2286	42.0348925	13.04575
G14	140	177.0427489	53.32214	22.0275328	9.716909
G14	210	413.6498918	116.9145	9.46150464	5.556503
G14	280	321.4150433	77.95441	52.1853239	22.32214
galaxolide	10	124.6482684	97.64068	70.0204211	10.12609
galaxolide	25	136.8235931	45.16144	71.0542457	10.38532
galaxolide	50	360.8901515	34.58411	50.0791698	12.82163
galaxolide	75	352.732684	176.0609	25.7330948	7.329411
galaxolide	100	1124.512987	403.3889	39.0061233	4.307021
galaxolide	200	5452.083333	214.1784	0.39176959	0.175241
lilial	10	76.14379085	29.78396	112.473206	10.38678
lilial	25	85	24.11677	109.237487	13.04749
lilial	50	74.64052288	13.62352	106.39943	10.49225
lilial	75	81.2745098	35.21009	106.893491	6.131873
lilial	100	139.7058824	120.7183	106.971888	6.409457
NHBE BMC Mix	12	X	NA	110.536908	9.501224
NHBE BMC Mix	23	X	NA	102.313311	7.562011
NHBE BMC Mix	58	X	NA	94.598601	3.89933
NHBE BMC Mix	92	X	NA	47.906738	16.43097
NHBE BMC Mix	115	X	NA	48.5063998	3.482424
NHBE BMC Mix	173	X	NA	27.1470688	3.99436
NHBE BMC Mix	230	X	NA	13.5151029	6.120792
NHBE Equi-Mix	30	100.0405844	35.06959	64.7342761	13.68268
NHBE Equi-Mix	75	771.3338745	384.8537	38.6674364	6.946066
NHBE Equi-Mix	150	4366.734307	803.8349	20.219014	6.004913
NHBE Equi-Mix	225	4536.539502	1004.149	4.31157289	5.243164
NHBE Equi-Mix	300	4593.249459	1149.5	0.3952752	0.272452
tonalide	10	82.98160173	16.0918	101.519964	9.62639
tonalide	25	117.3160173	33.81512	94.626949	10.41703
tonalide	50	741.8425325	422.7325	75.0003136	12.97595
tonalide	75	1756.953463	570.3399	64.7954273	7.973607
tonalide	100	3565.354437	963.4538	39.224567	8.219308
tonalide	200	4248.660714	1058.274	6.89529211	2.82307
TPP	10	74.49545455	121.1037	101.622347	8.174006
TPP	25	80.34393939	143.1379	93.1422202	7.59722
TPP	50	49.24090909	49.71872	105.588877	16.42341
TPP	75	51.51515152	58.96488	101.378472	9.074086
TPP	100	106.8757576	111.0092	75.1920659	7.114982
TPP	200	92.32272727	96.04242	72.9138189	6.676885
Vehicle Control	0	100	11.63325	100	11.56468
Vehicle Control	0	100	30.45223	100	13.5849
Vehicle Control	0	100	19.87257	100	10.18003
Vehicle Control	0	100	38.48331	100	7.826078

Table S4: Lowest effect level (LEL) for each endpoint across zebrafish and NHBE screening platforms for each chemical and mixture. Concentration units are in micromolar. X = not tested. See Table S1 for description of column identifiers.

Chemical	MO24	DP24	SM24	MORT	CRAN	AXIS	EDEM	MUSC	LTRK	BRN_	SKIN	NC__	TCHR	any.effect	EPR	LPR	Viability	Cytotox	Ox Stress
BBP	8	NA	NA	4.5	4	5	4	NA	NA	NA	NA	NA	NA	4	2.25	NA	NA	NA	NA
DIBP	10	NA	NA	5	8	8	8	10	NA	NA	NA	NA	10	4	2	NA	200	200	NA
DBP	5	10	NA	5	5	NA	NA	NA	NA	NA	NA	NA	NA	5	2	NA	NA	NA	NA
TPP	NA	NA	NA	10	6	8	8	NA	NA	NA	NA	NA	6	5	2	NA	100	NA	NA
BS	NA	NA	NA	22	14	18	14	18	18	NA	NA	NA	18	14	10	NA	NA	NA	NA
HHCB	NA	NA	NA	28	24	14	24	NA	32	32	NA	NA	24	16	10	NA	10	100	NA
AHTN	NA	NA	NA	NA	75	75	75	NA	60	75	NA	NA	NA	50	30	NA	50	50	NA
Lilial	NA	NA	NA	NA	70	90	70	NA	NA	NA	NA	NA	NA	70	40	NA	NA	NA	NA
BP	NA	74.8	NA	NA	74.8	NA	74.8	NA	74.8	74.8	NA	NA	74.8	74.8	35	NA	NA	NA	NA
BHT	NA	NA	NA	NA	NA	NA	NA	NA	NA	NA	NA	NA	NA	NA	NA	NA	25	100	NA
DEET	NA	NA	NA	NA	NA	NA	NA	NA	NA	NA	NA	NA	NA	NA	100	NA	NA	NA	NA
DEHP	NA	NA	NA	NA	NA	NA	NA	NA	NA	NA	NA	NA	NA	NA	NA	NA	NA	NA	NA
DEP	NA	NA	NA	NA	NA	NA	NA	NA	NA	NA	NA	NA	NA	NA	2.54	NA	NA	NA	NA
DNP	NA	NA	NA	NA	NA	NA	NA	NA	NA	NA	NA	NA	NA	NA	NA	NA	NA	NA	NA
ZF Equi-Mix	NA	NA	NA	7.5	5.1	6	5.1	NA	6	NA	NA	NA	NA	5.1	5.1	NA	X	X	X
ZF BMC Mix	0	0	0	6.13	8.16	8.16	8.16	0	6.13	0	0	0	0	6.13	2.041	NA	X	X	X
G14	112	56	NA	63	56	56	56	NA	56	56	NA	NA	56	56	28	NA	28	NA	NA
NHBE Equi-Mix	X	X	X	X	X	X	X	X	X	X	X	X	X	X	X	X	30	75	NA
NHBE BMC Mix	X	X	X	X	X	X	X	X	X	X	X	X	X	X	X	X	92.2	X	X

Table S5. Nominal exposure concentrations of individual components in BMC mix for each model system (µM)

Chemical	Zebrafish				NHBE			
	BBP	DBP	DIBP	Zfish BMC Mix	AHTN	HHCB	BHT	NHBE BMC Mix
Exposure Concentration	0.165	0.172	0.174	0.511	4.2	2.1	5.2	11.5
	0.329	0.343	0.348	1.02	8.5	4.2	10.3	23.0
	0.659	0.686	0.696	2.041	21.2	10.6	25.8	57.5
	1.32	1.37	1.39	4.08	34.0	16.9	41.2	92.0
	1.98	2.06	2.09	6.13	42.5	21.1	51.5	115.1
	2.63	2.75	2.78	8.16	63.7	31.7	77.3	172.6
	3.29	3.43	3.48	10.20	84.9	42.2	103.0	230.1

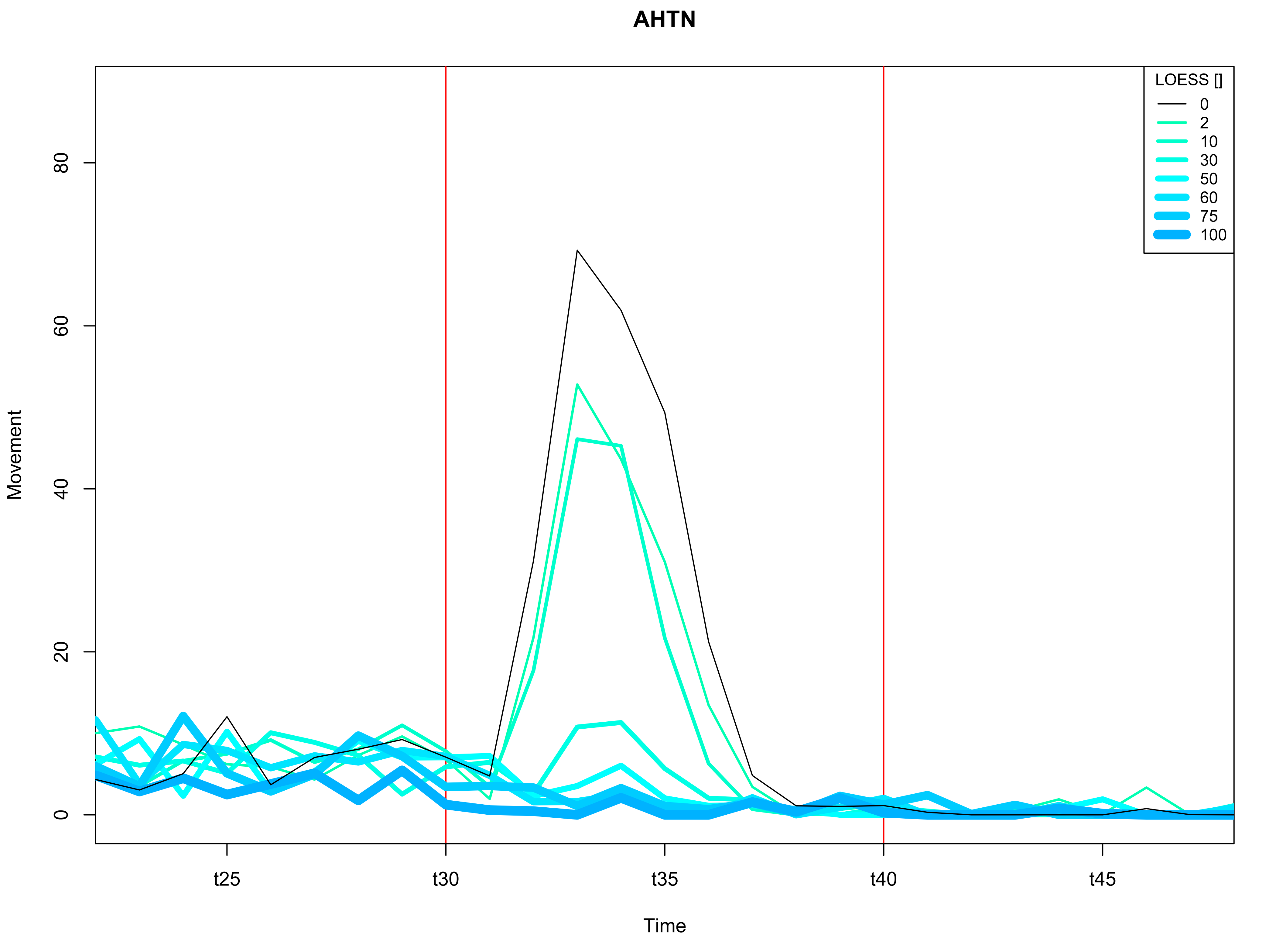

AHTN
LOESS []
0
2
10
30
50
60
75
100
Movement
0
20
40
60
80
Time
t25
t30
t35
t40
t45

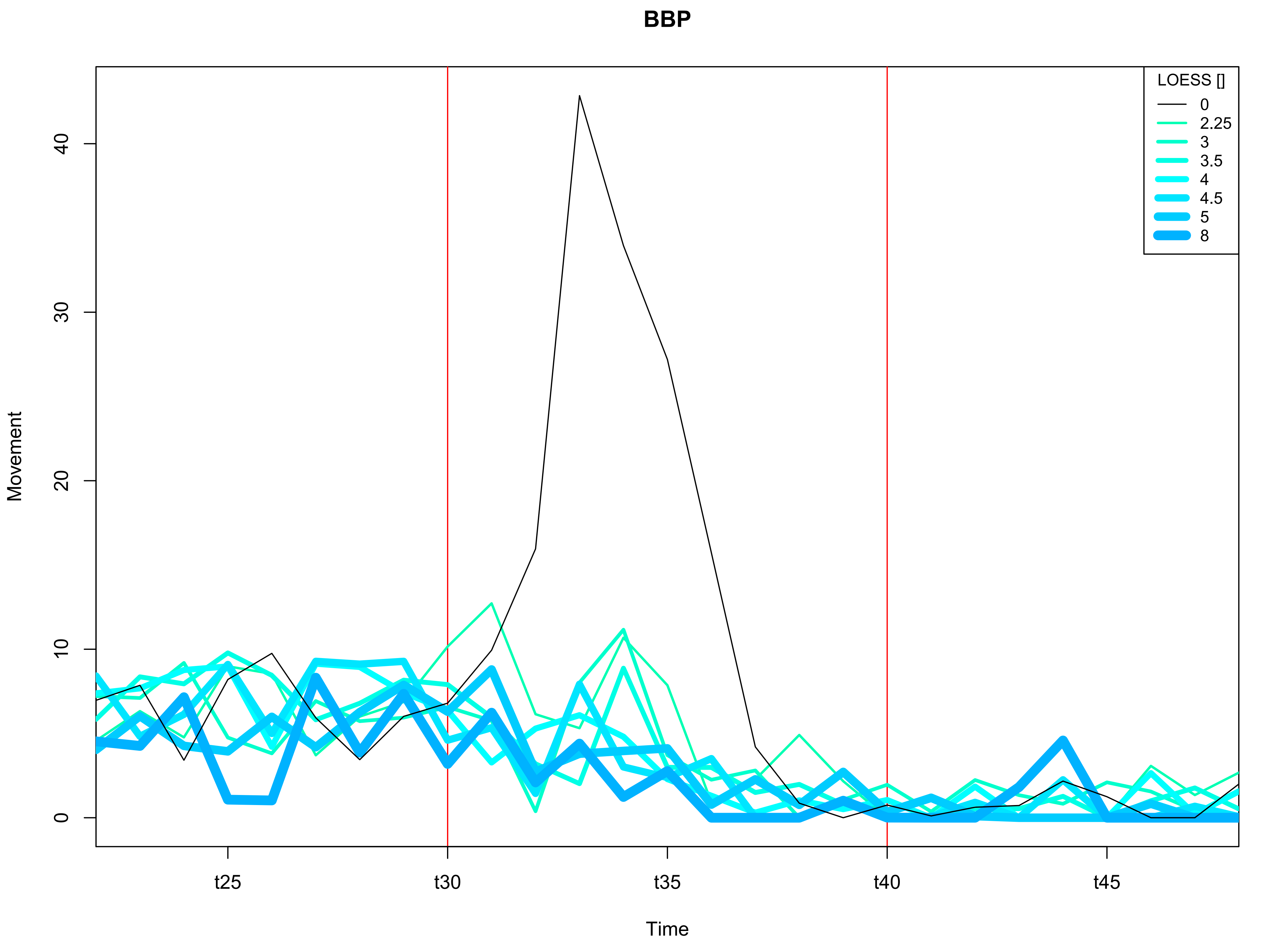
BBP
LOESS []
0
2.25
3
3.5
4
4.5
5
8
Movement
0
10
20
30
40
Time
t25
t30
t35
t40
t45

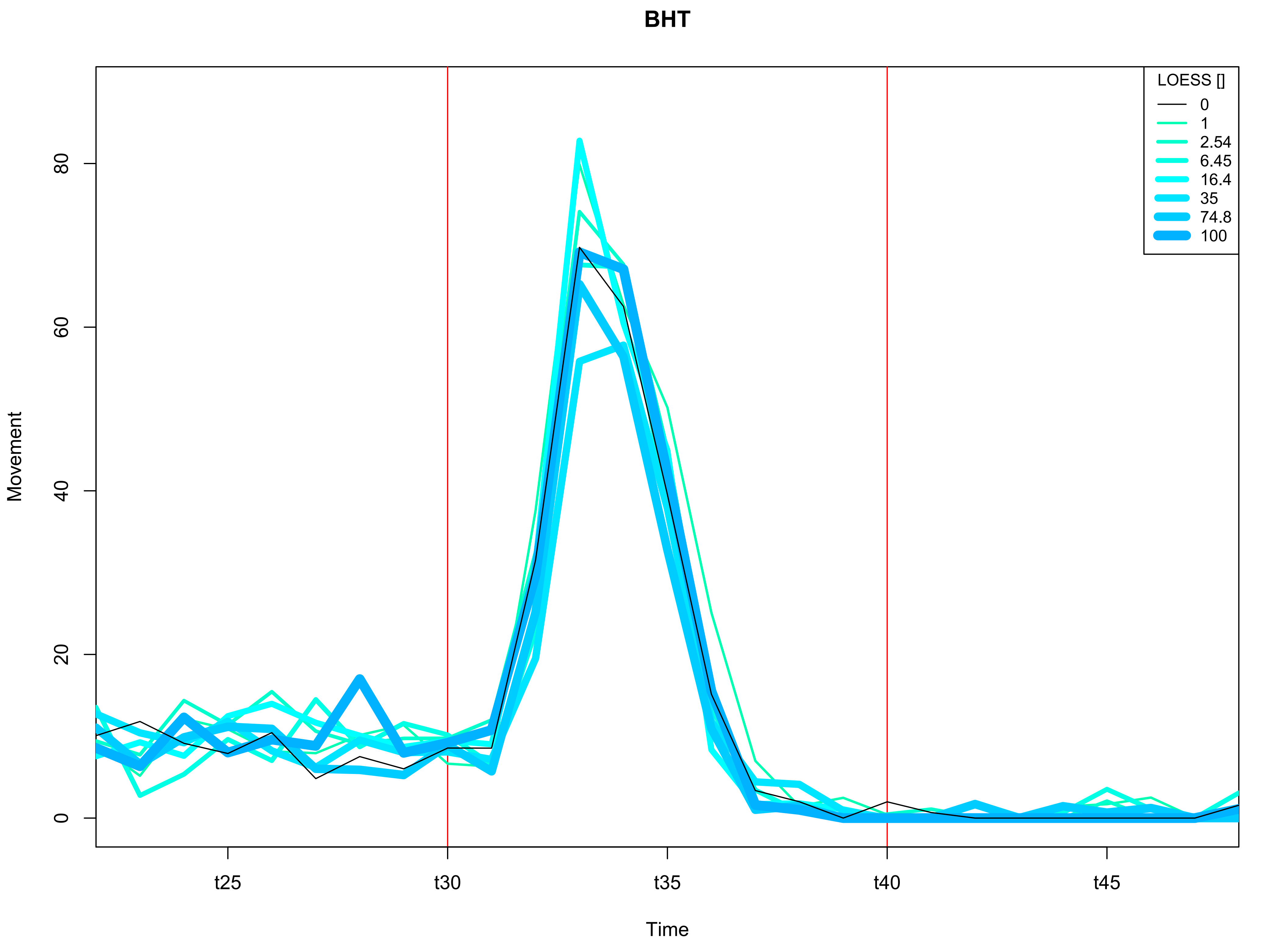

BHT
LOESS []
0
1
2.54
6.45
16.4
35
74.8
100
Movement
Time
0
20
40
60
80
t25
t30
t35
t40
t45

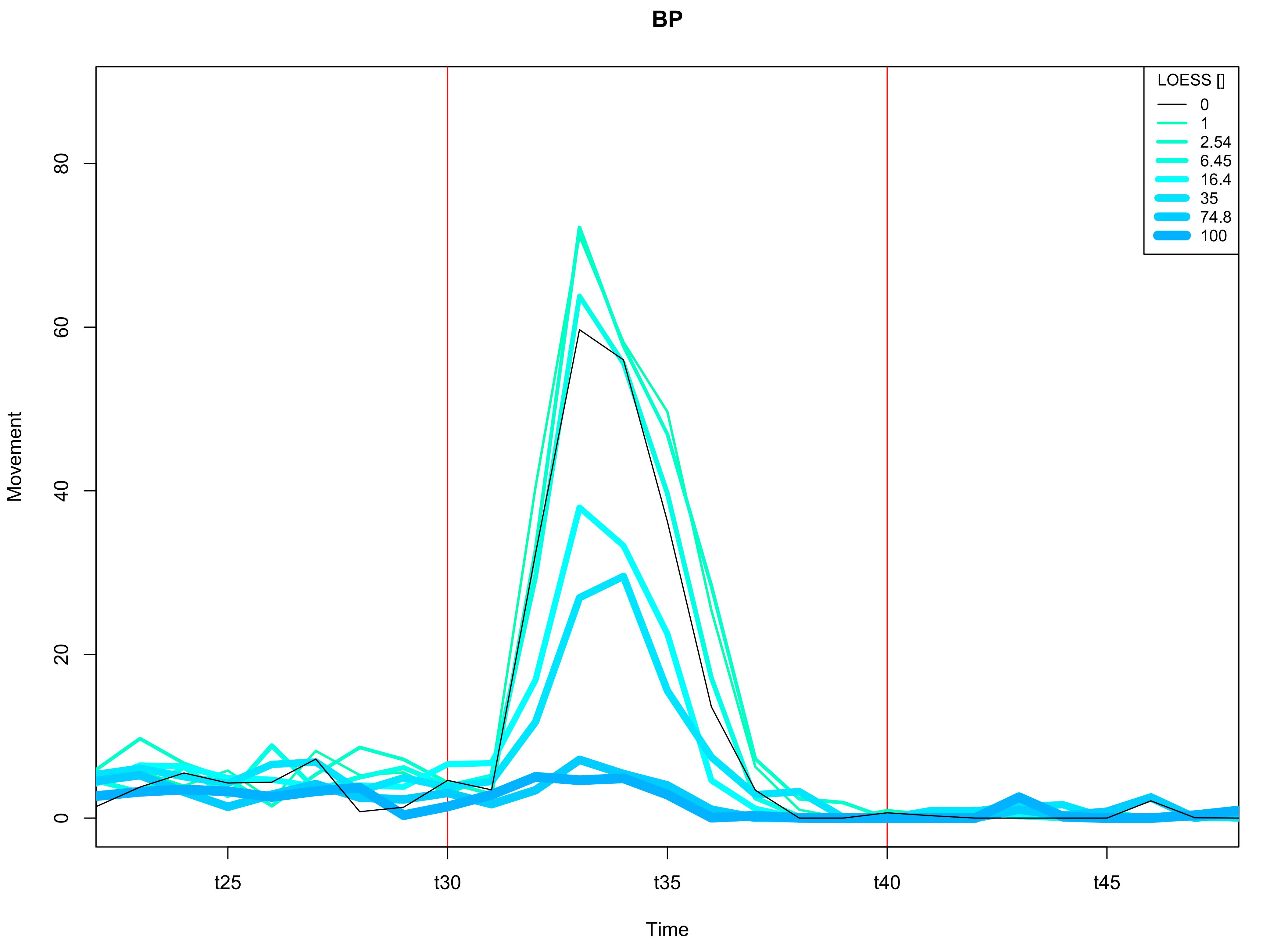
BP
LOESS []
0
1
2.54
6.45
16.4
35
74.8
100
Movement
0
20
40
60
80
t25
t30
t35
t40
t45
Time

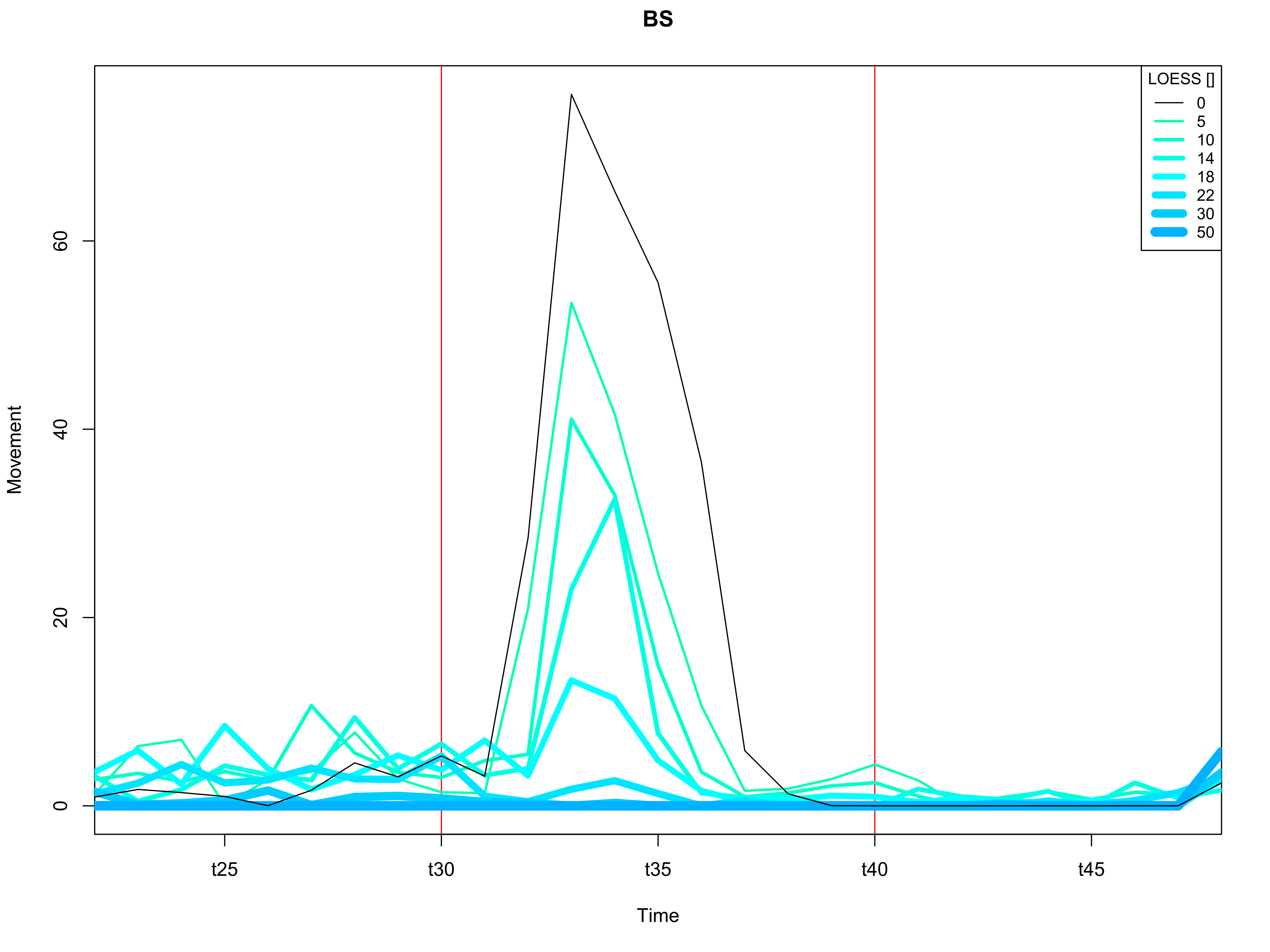

BS
LOESS []
0
5
10
14
18
22
30
50
Movement
0
20
40
60
Time
t25
t30
t35
t40
t45

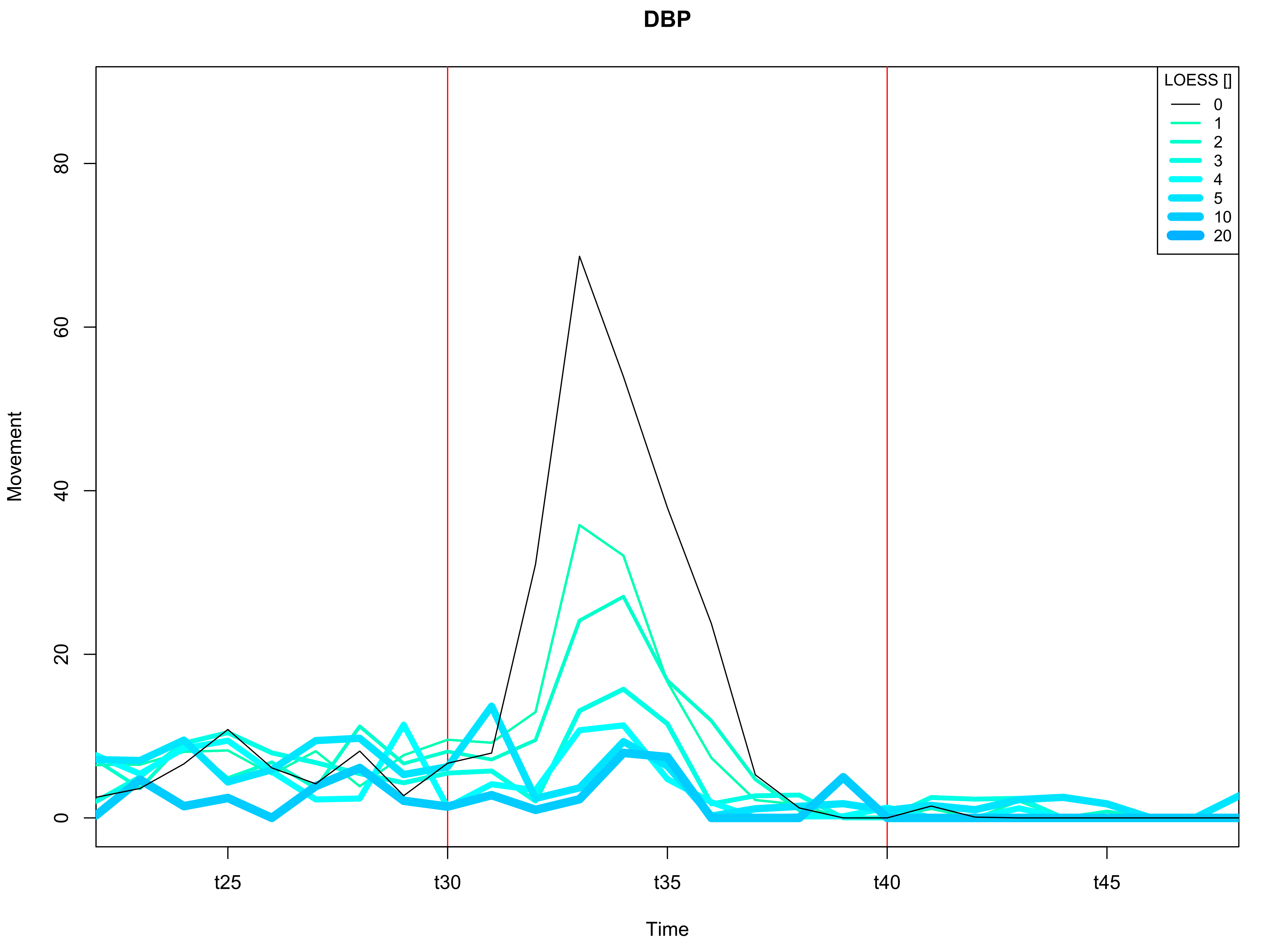

DBP
Movement
Time
0
20
40
60
80
t25
t30
t35
t40
t45
LOESS []
0
1
2
3
4
5
10
20

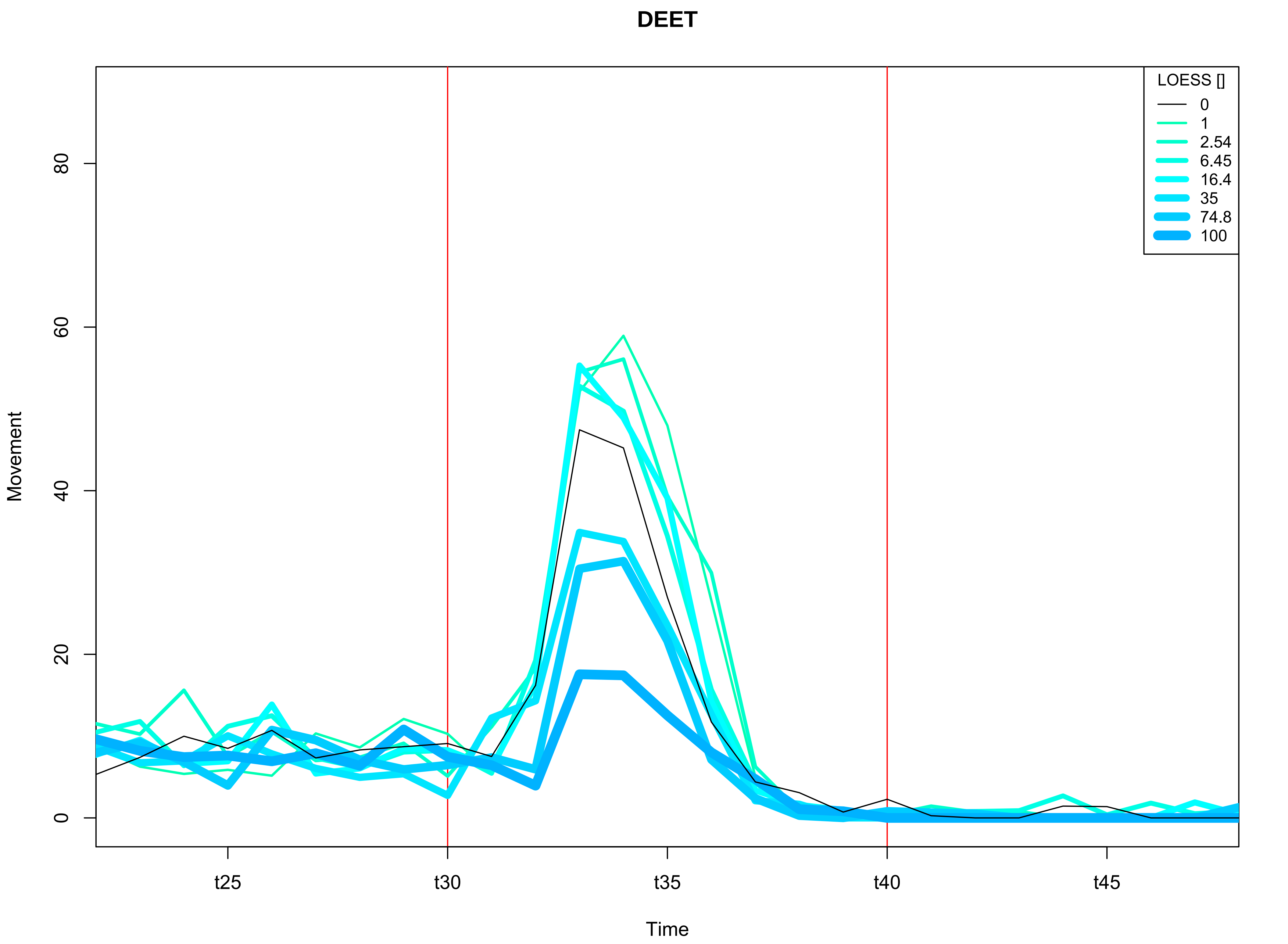
DEET
LOESS []
0
1
2.54
6.45
16.4
35
74.8
100
Movement
0
20
40
60
80
t25
t30
t35
t40
t45
Time

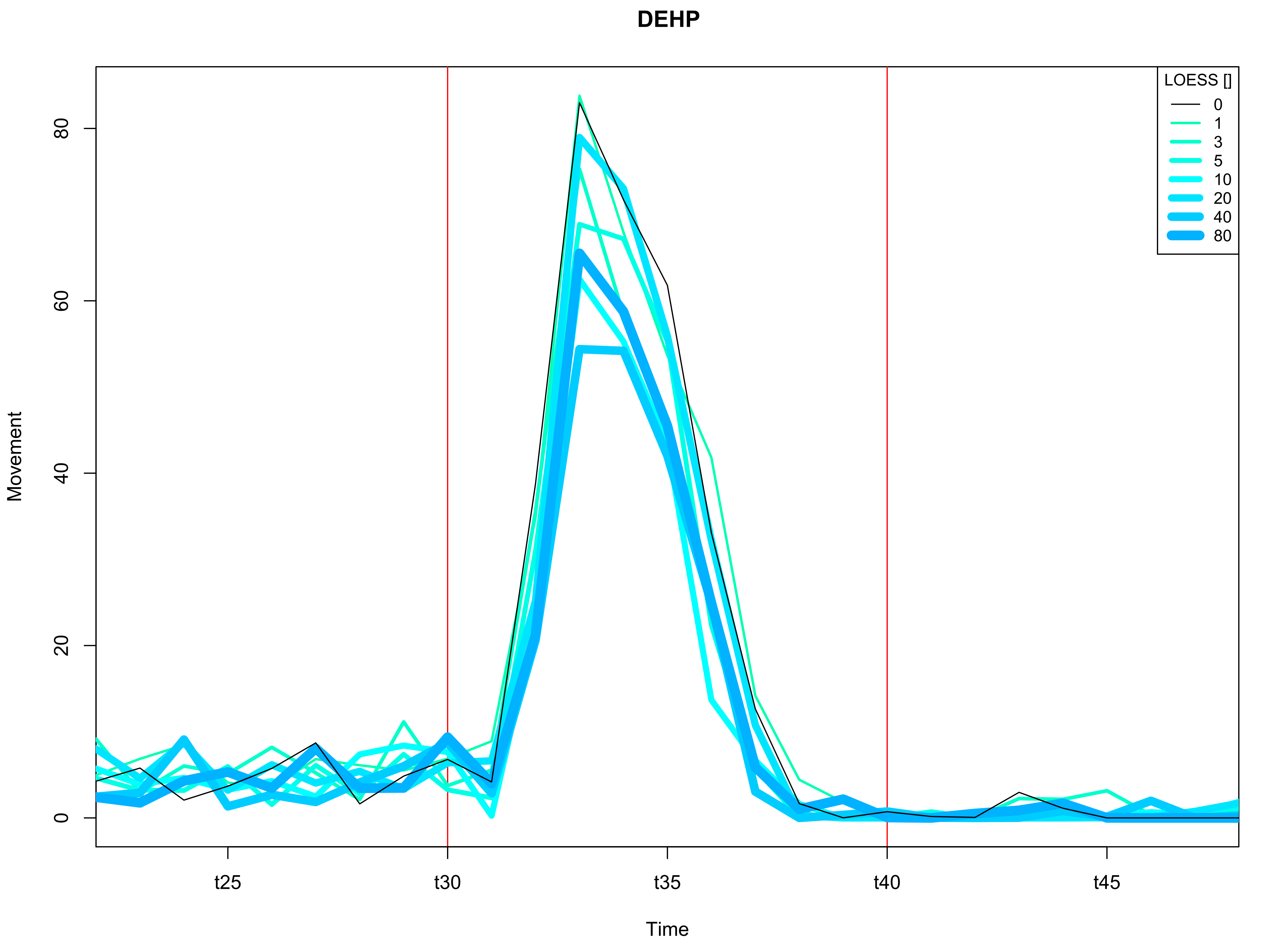
DEHP
Movement
Time
0
20
40
60
80
t25
t30
t35
t40
t45
LOESS []
0
1
3
5
10
20
40
80

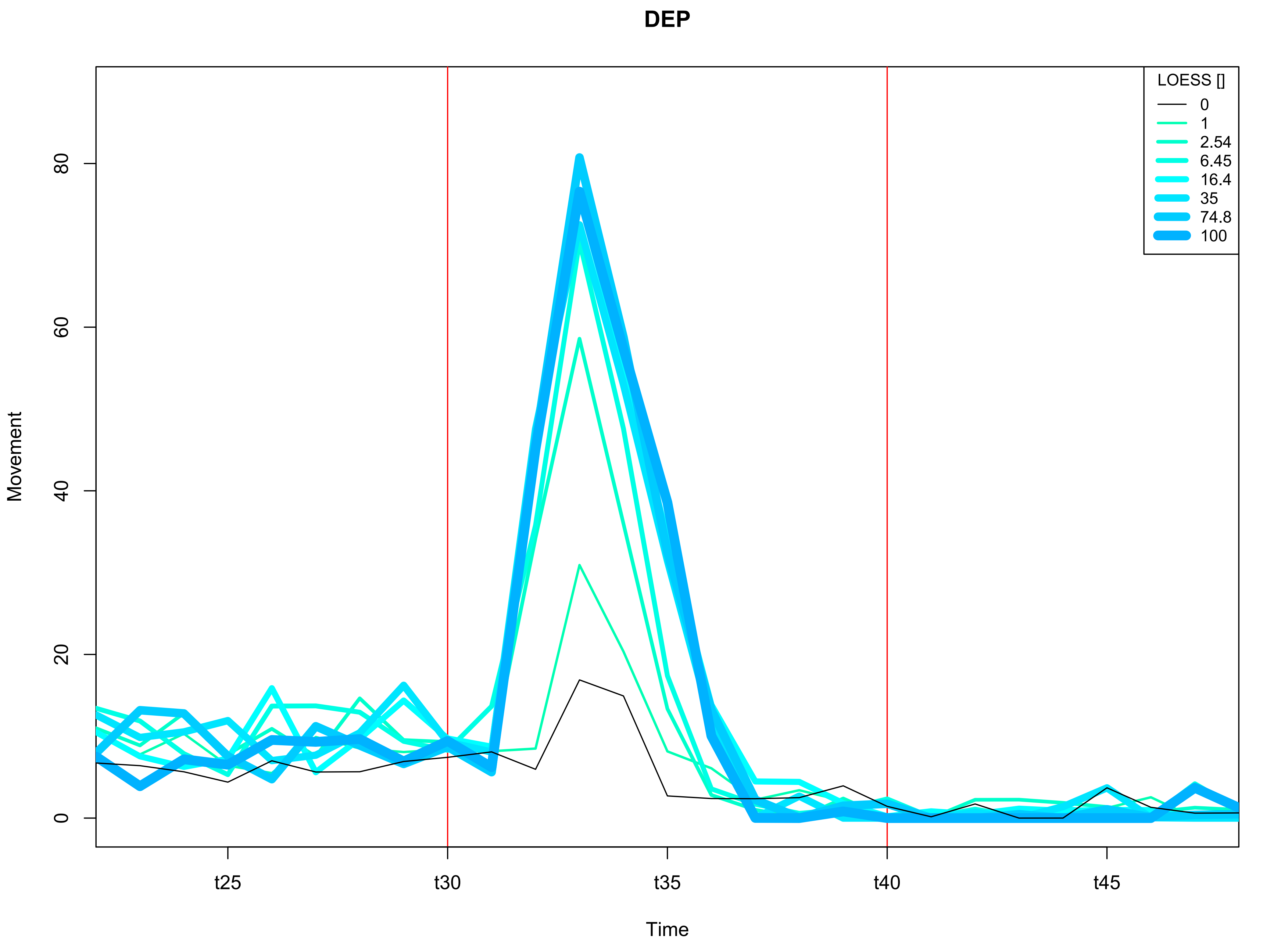
DEP
LOESS []
0
1
2.54
6.45
16.4
35
74.8
100
Movement
0
20
40
60
80
Time
t25
t30
t35
t40
t45

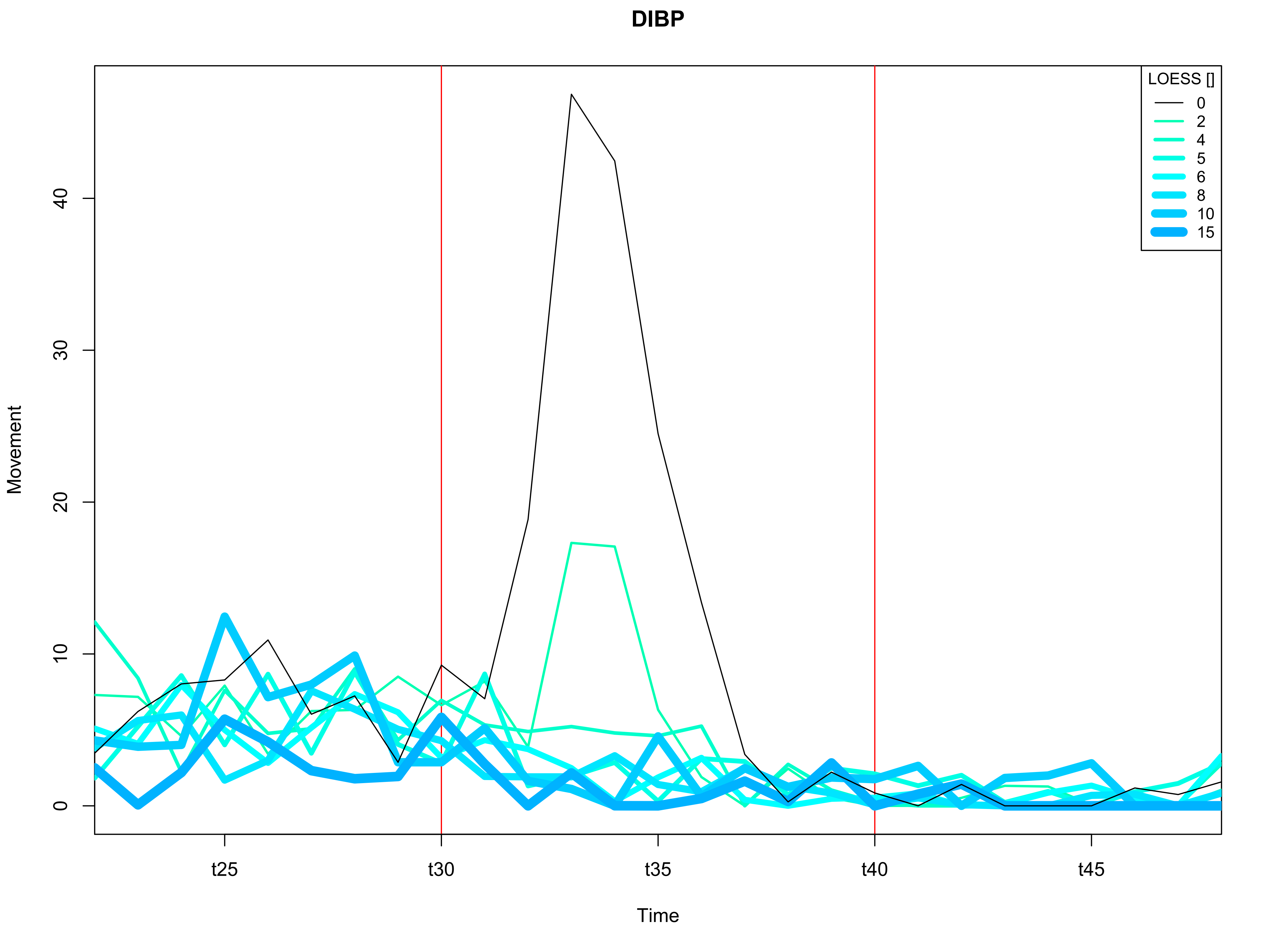

DIBP
LOESS []
0
2
4
5
6
8
10
15
Movement
0
10
20
30
40
Time
t25
t30
t35
t40
t45

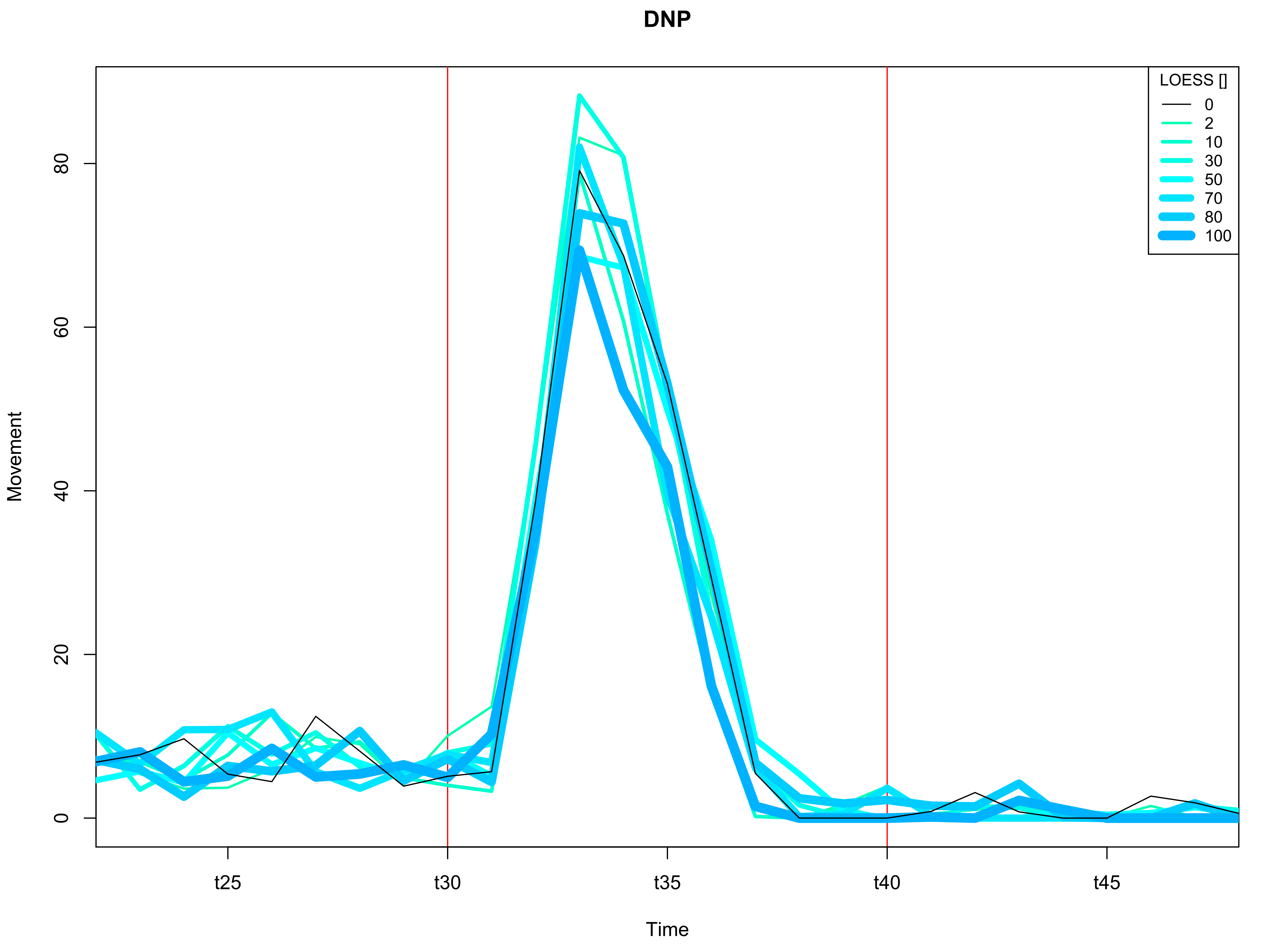

DNP
LOESS []
0
2
10
30
50
70
80
100
Movement
0
20
40
60
80
Time
t25
t30
t35
t40
t45

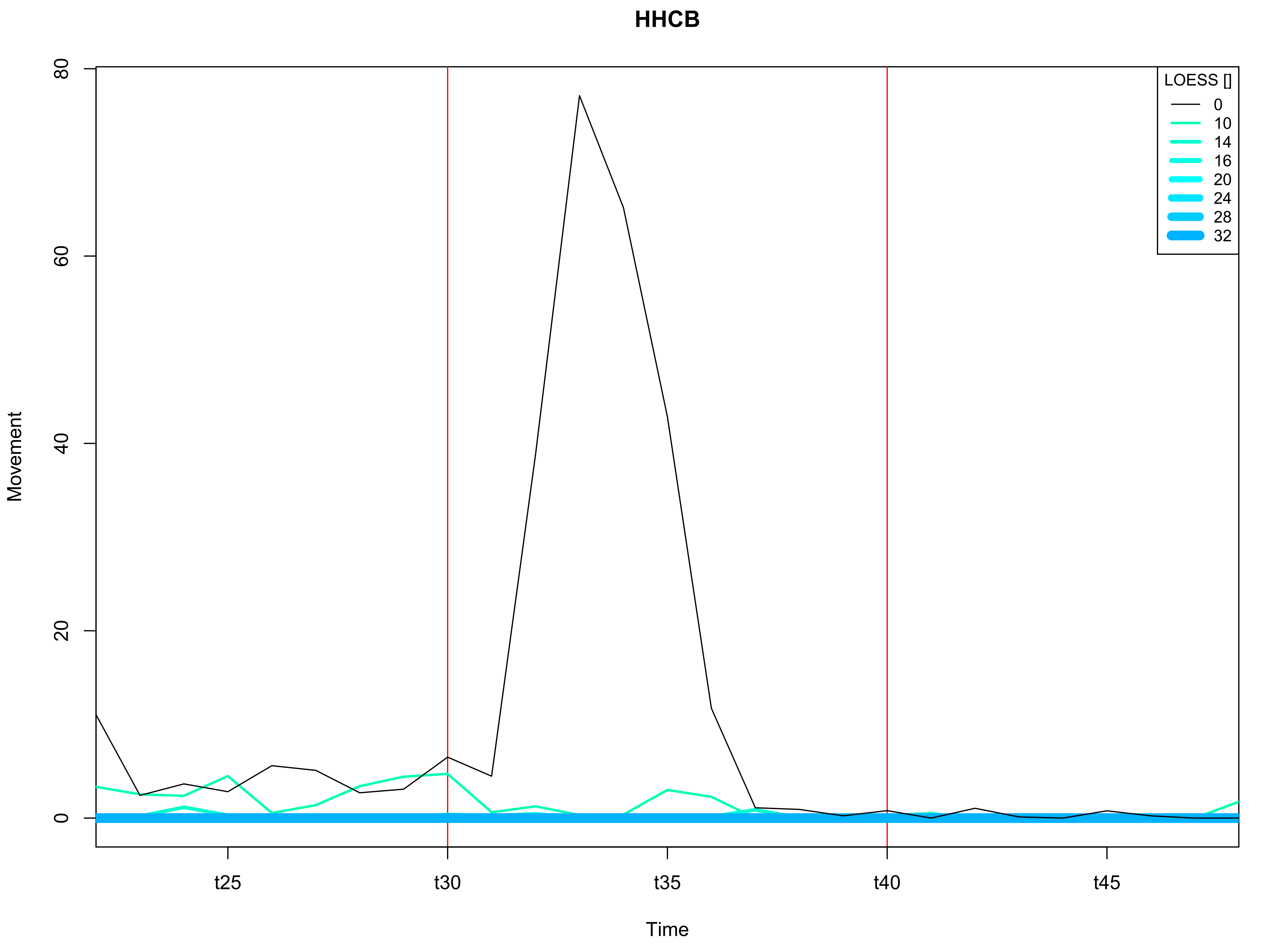

HHCB
LOESS []
0
10
14
16
20
24
28
32
Movement
0
20
40
60
80
t25
t30
t35
t40
t45
Time

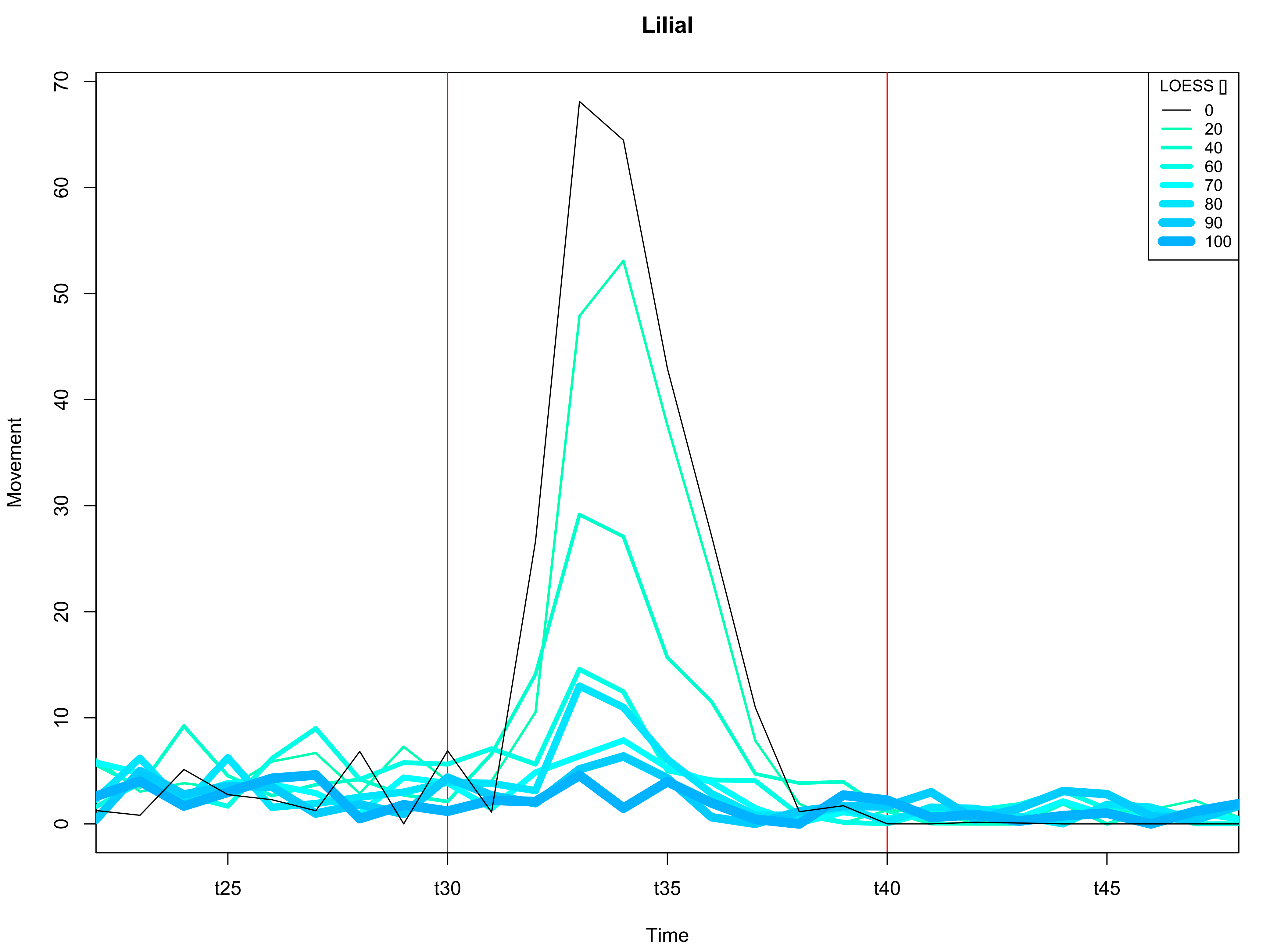

Lilial
Movement
Time
0
10
20
30
40
50
60
70
t25
t30
t35
t40
t45
LOESS []
0
20
40
60
70
80
90
100

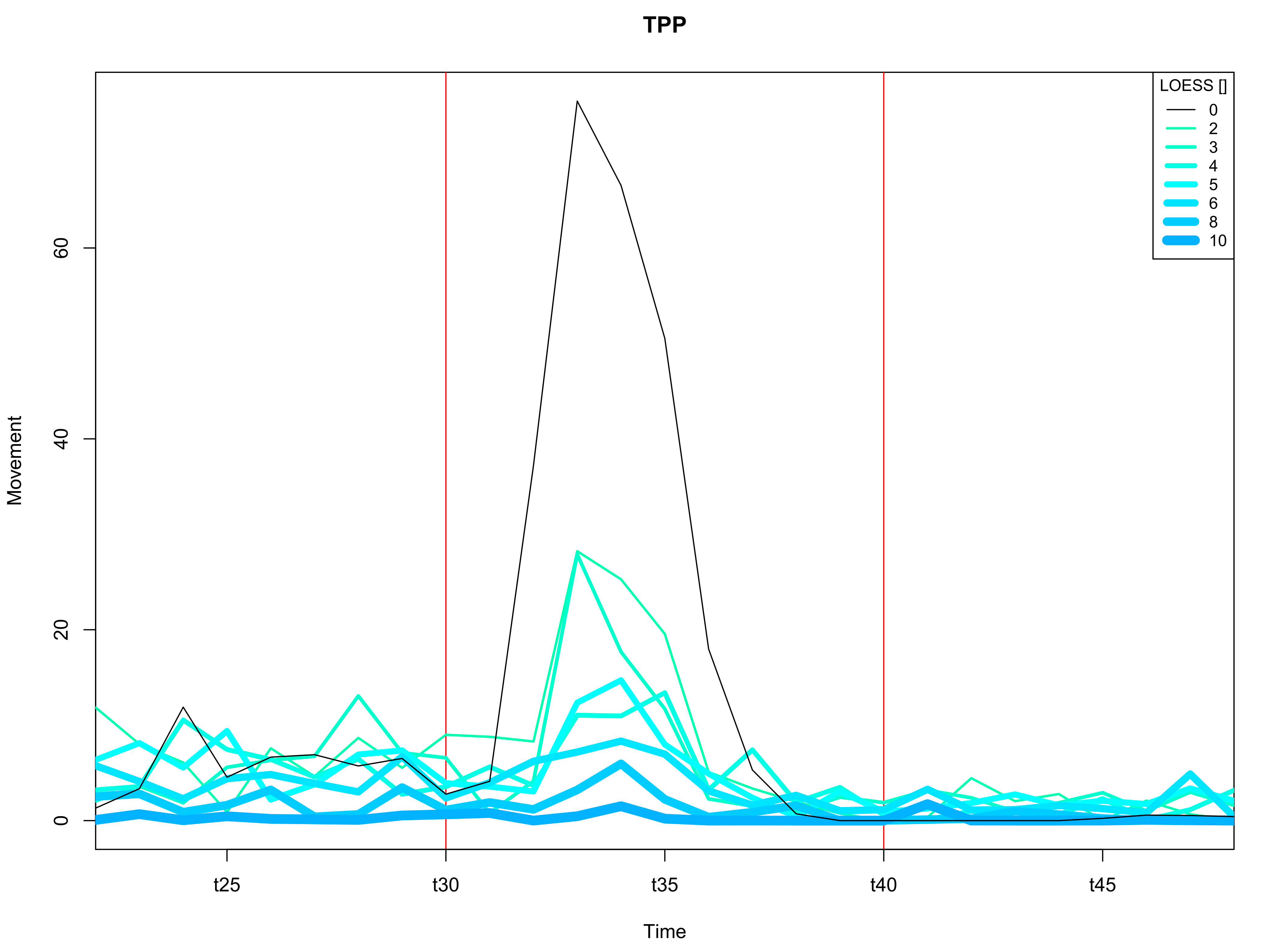

TPP
LOESS []
0
2
3
4
5
6
8
10
Movement
0
20
40
60
Time
t25
t30
t35
t40
t45

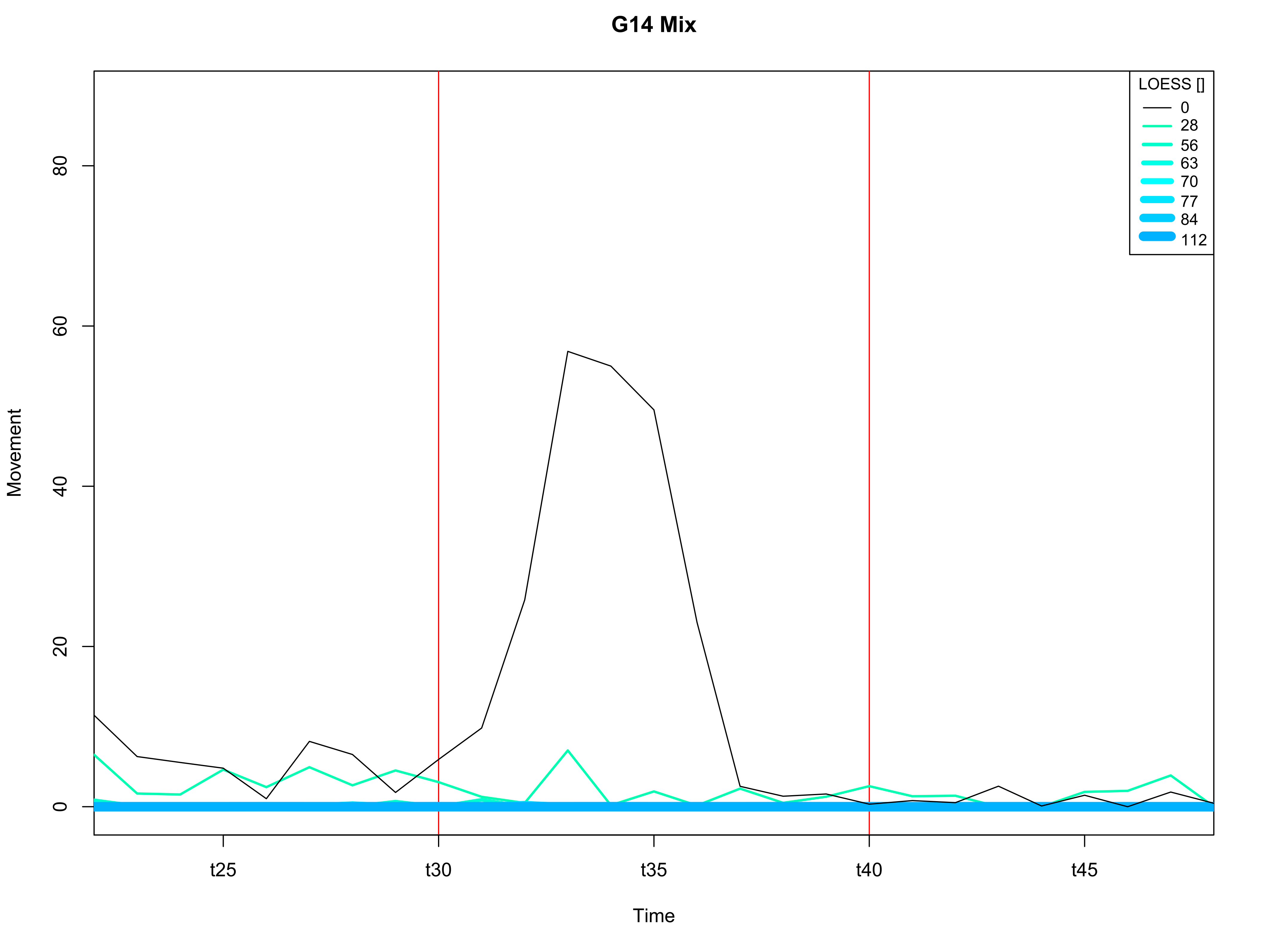
G14 Mix
LOESS []
0
28
56
63
70
77
84
112
Movement
0
20
40
60
80
t25
t30
t35
t40
t45
Time

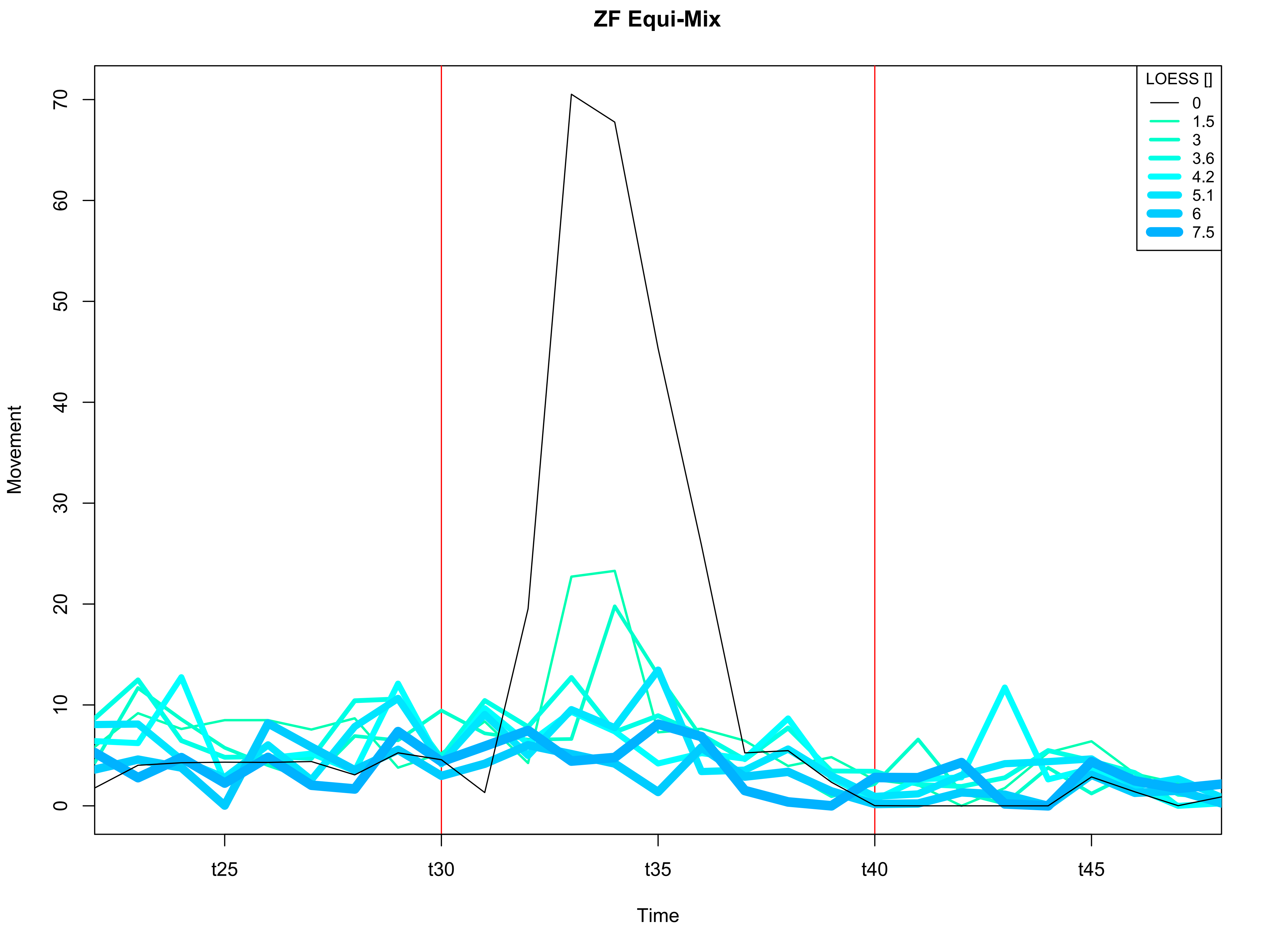

ZF Equi-Mix
LOESS []
0
1.5
3
3.6
4.2
5.1
6
7.5
Movement
0
10
20
30
40
50
60
70
Time
t25
t30
t35
t40
t45

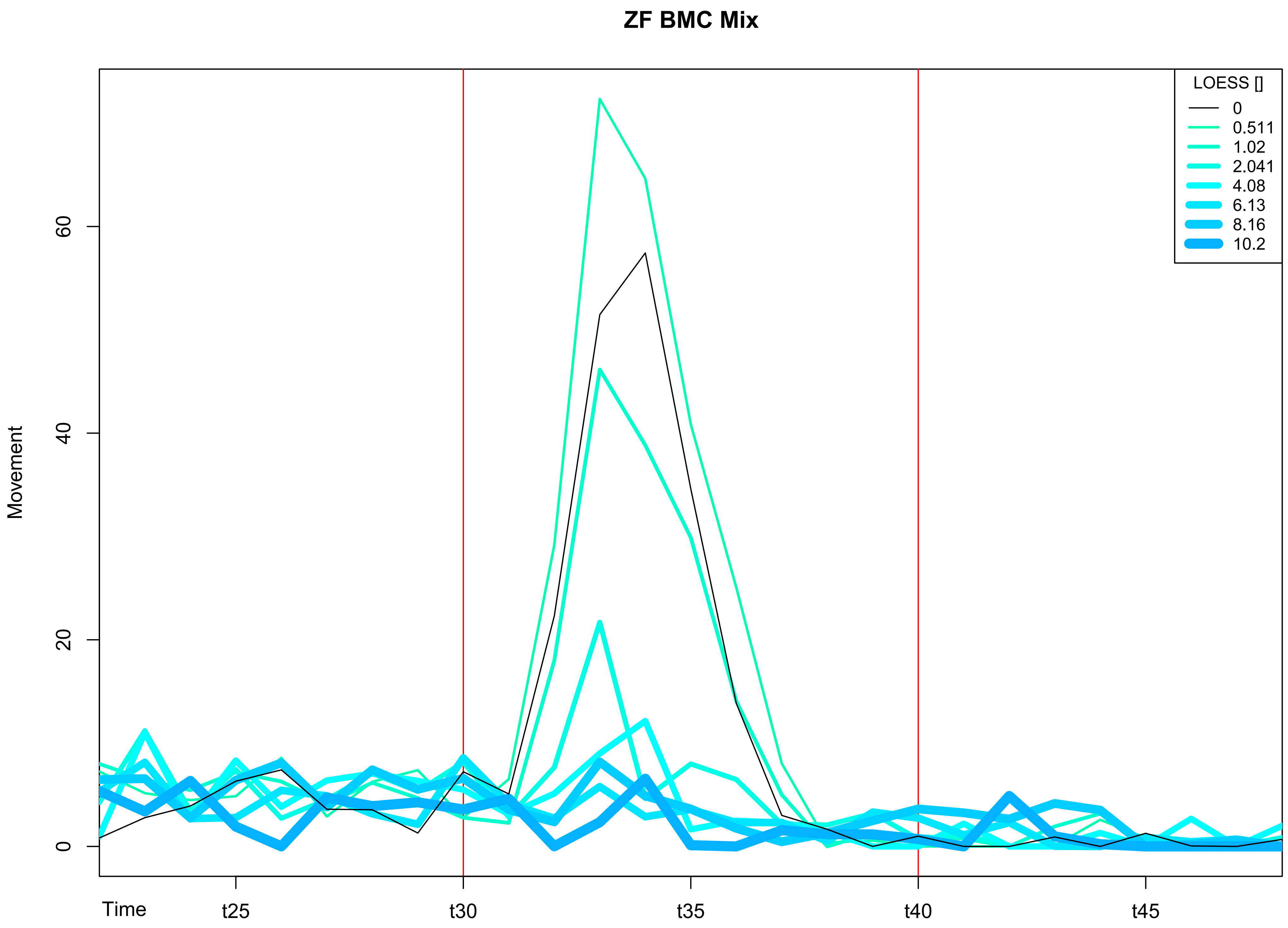

Figure S9. Movement versus time plots for zebrafish EPR for each chemical and mixture. Red lines delineate background, excitatory, and refractory periods.

Table S6. Embryonic photomotor response (EPR) compiled data for standard and chorion-on conditions. B = background; E = excitation; R = refractory. For significance column, 1 = significant, 0 = not significant.

chemical.name	concentration (μM)	Interval	Movement.peak	Movement.sd	Movement.n	ks.stat	ks.pval	diff	delta	sig
AHTN	0	B	3.592234239	11.08448112	315	0	1	0	0	0
AHTN	0	E	21.48596055	38.43747733	315	0	1	0	0	0
AHTN	0	R	0.228097092	3.314125031	280	0	1	0	0	0
AHTN	10	B	4.914847833	12.70615111	315	0.066666667	0.485805331	1.322613594	0.368186902	0
AHTN	10	E	21.45158123	39.54982964	315	0.034920635	0.990716312	-0.034379313	-0.001600083	0
AHTN	10	R	1.537233332	10.42573299	280	0.025	0.999993621	1.30913624	5.739381544	0
AHTN	30	B	4.605114462	12.23277016	324	0.042592593	0.93409286	1.012880223	0.281963858	0
AHTN	30	E	5.284020508	15.33081809	324	0.236684303	3.38E-08	-16.20194004	-0.754071013	1
AHTN	30	R	0.542308712	4.464145627	288	0.017162698	1	0.31421162	1.377534529	0
AHTN	50	B	4.738491264	11.19085184	306	0.092156863	0.143172114	1.146257025	0.319093063	0
AHTN	50	E	2.949544973	8.628531669	306	0.259290383	1.73E-09	-18.53641557	-0.86272222	1
AHTN	50	R	0.470409417	3.497790733	272	0.018592437	1	0.242312325	1.062320976	0
AHTN	60	B	5.244157468	11.13466755	315	0.120634921	0.020425302	1.651923229	0.459859552	0
AHTN	60	E	3.929949659	12.60836827	315	0.276190476	7.34E-11	-17.55601089	-0.817092205	1
AHTN	60	R	0.926493923	5.779300716	280	0.032142857	0.998697677	0.698396831	3.061840138	0
AHTN	70	B	4.855484375	10.29012908	297	0.116017316	0.032645129	1.263250136	0.351661404	0
AHTN	70	E	1.531883912	5.456803484	297	0.308898509	4.28E-13	-19.95407663	-0.928703029	1
AHTN	70	R	1.201879052	5.682355636	264	0.061038961	0.691977884	0.97378196	4.269155525	0
AHTN	80	B	4.121863799	9.352289281	279	0.110803891	0.052871235	0.52962956	0.147437368	0
AHTN	80	E	2.022741324	6.081573117	279	0.27014849	8.36E-10	-19.46321922	-0.905857533	1
AHTN	80	R	1.214927634	6.800147385	248	0.053341014	0.848400899	0.986830542	4.326361786	0
AHTN	100	B	3.971147412	8.672994407	315	0.133333333	0.007395727	0.378913173	0.105481199	0
AHTN	100	E	1.208113261	5.398625965	315	0.320634921	1.72E-14	-20.27784729	-0.943771969	1
AHTN	100	R	0.810856864	4.317290452	280	0.05	0.875195794	0.582759772	2.554875939	0
BBP	0	B	6.479689714	13.1465797	315	0	1	0	0	0
BBP	0	E	16.74320678	26.45710722	315	0	1	0	0	0
BBP	0	R	0.859452041	6.144433438	280	0	1	0	0	0
BBP	2.25	B	6.87538774	14.67559225	324	0.036419753	0.983894313	0.395698025	0.061067434	0
BBP	2.25	E	5.886904063	15.51490623	324	0.255467372	1.77E-09	-10.85630272	-0.648400444	1
BBP	2.25	R	0.887119682	7.518758416	288	0.011507937	1	0.027667642	0.032192188	0
BBP	3	B	6.383569057	12.29287782	297	0.035594036	0.99024792	-0.096120658	-0.014834145	0
BBP	3	E	3.907126886	12.1421408	297	0.291871092	9.77E-12	-12.83607989	-0.766644052	1
BBP	3	R	1.106770784	6.428979859	264	0.021320346	0.999999979	0.247318743	0.287763286	0
BBP	3.5	B	7.467476216	13.58283923	306	0.06302521	0.5683867	0.987786502	0.152443488	0
BBP	3.5	E	3.364637257	10.30871153	306	0.28898226	1.10E-11	-13.37856952	-0.799044633	1
BBP	3.5	R	0.704018233	4.463320774	272	0.010714286	1	-0.155433807	-0.180852218	0
BBP	4	B	7.484844275	13.46549097	315	0.088888889	0.165909354	1.005154561	0.155123872	0
BBP	4	E	2.766548276	9.456435976	315	0.314285714	6.14E-14	-13.9766585	-0.834765925	1
BBP	4	R	0.782412492	5.870696029	280	0.014285714	1	-0.077039548	-0.089637984	0

BBP	4.5	B	7.468309072	14.65310041	315	0.06031746	0.615432501	0.988619358	0.152572021	0
BBP	4.5	E	2.730378905	9.957339941	315	0.333333333	1.22E-15	-14.01282787	-0.836926167	1
BBP	4.5	R	0.497891751	5.193156246	280	0.017857143	1	-0.36156029	-0.420686987	0
BBP	5	B	5.557470203	11.89603334	306	0.048646125	0.856152922	-0.922219511	-0.142324641	0
BBP	5	E	3.327830064	11.74376257	306	0.307936508	3.29E-13	-13.41537671	-0.801242969	1
BBP	5	R	0.264086896	2.979613567	272	0.021218487	0.999999976	-0.595365145	-0.692726431	0
BBP	8	B	4.592014065	11.23174708	180	0.073809524	0.560572153	-1.88767565	-0.29132192	0
BBP	8	E	1.971330325	7.42940629	180	0.328571429	3.63E-11	-14.77187645	-0.882260886	1
BBP	8	R	0.800645423	7.782513657	160	0.026785714	0.999999571	-0.058806618	-0.068423385	0
BHT	0	B	3.878804243	11.84619925	306	0	1	0	0	0
BHT	0	E	24.56909242	41.37163012	306	0	1	0	0	0
BHT	0	R	0.008457158	0.139479017	272	0	1	0	0	0
BHT	1	B	4.530554356	13.11685096	306	0.032679739	0.996738369	0.651750113	0.168028617	0
BHT	1	E	28.20974774	39.26771406	306	0.104575163	0.070418328	3.640655315	0.148180293	0
BHT	1	R	0.292170789	3.303903665	272	0.011029412	1	0.283713631	33.54716128	0
BHT	2.54	B	4.365864496	12.34948754	324	0.033587509	0.99429185	0.487060253	0.125569692	0
BHT	2.54	E	23.51646861	37.77271043	324	0.035221496	0.989781516	-1.052623809	-0.042843414	0
BHT	2.54	R	0.098861919	1.676459663	288	0.003472222	1	0.090404761	10.6897335	0
BHT	6.45	B	4.9922567	13.74263098	324	0.033587509	0.99429185	1.113452457	0.287060751	0
BHT	6.45	E	17.99353211	34.20201143	324	0.085330428	0.201973668	-6.575560315	-0.267635458	0
BHT	6.45	R	0.354003995	3.546859726	288	0.017156863	1	0.345546837	40.85850735	0
BHT	16.4	B	4.680334871	13.11945341	315	0.033146592	0.99562075	0.801530628	0.206643743	0
BHT	16.4	E	23.99863618	37.37922381	315	0.053034547	0.775196727	-0.57045624	-0.02321845	0
BHT	16.4	R	0.638950796	6.171225631	280	0.010714286	1	0.630493639	74.55148246	0
BHT	35	B	4.953141228	12.0401046	324	0.056281772	0.701154408	1.074336985	0.276976335	0
BHT	35	E	22.15999759	36.34081261	324	0.035947712	0.987108925	-2.409094834	-0.09805388	0
BHT	35	R	0.233742157	2.830847153	288	0.006944444	1	0.225284999	26.63838247	0
BHT	74.8	B	4.737860564	13.12246137	315	0.03557423	0.989410592	0.859056321	0.221474523	0
BHT	74.8	E	21.39949759	35.35216418	315	0.051820728	0.798770587	-3.16959483	-0.129007404	0
BHT	74.8	R	0.560050855	4.834550891	280	0.017857143	1	0.551593698	65.22211385	0
BHT	100	B	4.002838303	11.37288873	315	0.029505135	0.999261105	0.12403406	0.031977396	0
BHT	100	E	15.5780836	29.41271636	315	0.11372549	0.036085543	-8.991008822	-0.365947943	0
BHT	100	R	1.087472048	7.75908644	280	0.035609244	0.994810796	1.07901489	127.5859973	0
BP	0	B	3.655194573	10.75814831	306	0	1	0	0	0
BP	0	E	27.69515756	44.41349847	306	0	1	0	0	0
BP	0	R	0.165871955	2.116876849	272	0	1	0	0	0
BP	20	B	5.398555754	12.64267663	324	0.078068264	0.292821493	1.743361181	0.476954413	0
BP	20	E	16.7473773	29.57155197	324	0.111837328	0.039025727	-10.94778026	-0.395295829	0
BP	20	R	0.792100824	6.370107259	288	0.017156863	1	0.626228869	3.77537521	0
BP	40	B	3.778658785	11.22992355	315	0.040149393	0.963813131	0.123464212	0.03377774	0

BP	40	E	9.9393053	24.17197678	315	0.20140056	6.80E-06	-17.75585226	-0.641117575	1
BP	40	R	0.790085577	4.583586963	280	0.028361345	0.999888151	0.624213621	3.763225795	0
BP	50	B	4.919785348	12.20165758	324	0.081154684	0.251133918	1.264590776	0.345970851	0
BP	50	E	8.491940571	18.46887788	324	0.197167756	9.71E-06	-19.20321699	-0.693378146	1
BP	50	R	0.996229192	5.993321448	288	0.034313725	0.996550718	0.830357236	5.006013429	0
BP	60	B	4.246604377	11.15084118	297	0.041592395	0.956746136	0.591409805	0.161799815	0
BP	60	E	7.243879555	16.82514594	297	0.222519311	6.59E-07	-20.451278	-0.738442378	1
BP	60	R	2.177619461	8.180751609	264	0.098707665	0.146923012	2.011747506	12.12831609	0
BP	70	B	4.464883512	11.0769418	306	0.052287582	0.797010175	0.80968894	0.221517329	0
BP	70	E	6.369997028	14.93944507	306	0.22875817	2.22E-07	-21.32516053	-0.769996	1
BP	70	R	1.790205288	7.023721431	272	0.073529412	0.454008497	1.624333332	9.792694179	0
BP	80	B	3.626615872	9.788269006	297	0.022974847	0.999998364	-0.0285787	-0.007818654	0
BP	80	E	4.572196589	11.92012596	297	0.25103981	1.12E-08	-23.12296097	-0.834909891	1
BP	80	R	0.702417829	4.475380374	264	0.026737968	0.999979376	0.536545873	3.234699151	0
BP	100	B	2.415308148	8.294408012	306	0.04248366	0.945264713	-1.239886425	-0.339212154	0
BP	100	E	2.175245092	8.296539279	306	0.343137255	4.44E-16	-25.51991247	-0.921457566	1
BP	100	R	0.941058195	5.646253868	272	0.033088235	0.998362206	0.77518624	4.673401466	0
BS	0	B	2.084986428	8.774449899	315	0	1	0	0	0
BS	0	E	30.18573687	42.07117213	315	0	1	0	0	0
BS	0	R	0.305601944	1.68571079	280	0	1	0	0	0
BS	5	B	3.760126997	11.13496634	315	0.073015873	0.370570972	1.675140569	0.803429963	0
BS	5	E	17.65466496	30.67437898	315	0.174603175	0.000135028	-12.5310719	-0.415132218	0
BS	5	R	0.926106585	5.659940211	280	0.028571429	0.999847967	0.620504641	2.030434204	0
BS	10	B	4.300847139	11.73743075	306	0.10681606	0.057911707	2.215860711	1.062769849	0
BS	10	E	11.91583498	24.2450803	306	0.283940243	2.70E-11	-18.26990189	-0.605249492	1
BS	10	R	1.232830377	6.401546017	272	0.038130252	0.988060956	0.927228433	3.034105148	0
BS	14	B	3.621870465	10.77052221	288	0.118253968	0.029760317	1.536884037	0.737119444	0
BS	14	E	8.198979929	21.48808771	288	0.350694444	2.22E-16	-21.98675694	-0.728382316	1
BS	14	R	1.235453328	6.63290876	256	0.0359375	0.995232851	0.929851384	3.042688049	0
BS	18	B	4.112554249	10.79809309	306	0.152567694	0.001454908	2.02756782	0.97246092	1
BS	18	E	4.822175489	12.90683727	306	0.375443511	0	-25.36356138	-0.840249867	1
BS	18	R	0.607240101	3.574439833	272	0.019852941	0.999999998	0.301638156	0.987029572	0
BS	22	B	2.861345091	7.314234103	315	0.165079365	0.000374103	0.776358663	0.372356699	0
BS	22	E	0.862199581	5.228490655	315	0.511111111	0	-29.32353728	-0.971436855	1
BS	22	R	0.699714503	4.420357305	280	0.028571429	0.999847967	0.394112559	1.289627131	0
BS	30	B	0.688726168	2.748941856	315	0.073015873	0.370570972	-1.39626026	-0.669673548	0
BS	30	E	0.115534063	1.000749571	315	0.533333333	0	-30.0702028	-0.996172561	1
BS	30	R	0.477352919	4.01328821	280	0.025	0.999993621	0.171750975	0.562008776	0
BS	50	B	0.000678168	0.011508899	288	0.076190476	0.346858738	-2.08430826	-0.999674737	0
BS	50	E	0	0	288	0.568253968	0	-30.18573687	-1	1

BS	50	R	0.718943437	6.011746215	256	0.016183036	1	0.413341493	1.352548634	0
DBP	0	B	5.827035352	13.53683716	306	0	1	0	0	0
DBP	0	E	25.52281807	37.50485769	306	0	1	0	0	0
DBP	0	R	0.192998801	2.991431961	272	0	1	0	0	0
DBP	1	B	6.800206482	13.32723138	297	0.057239057	0.706728303	0.97317113	0.167009649	0
DBP	1	E	13.0835267	25.45440845	297	0.188057041	4.69E-05	-12.43929137	-0.487379228	0
DBP	1	R	0.391693397	3.703192965	264	0.007687166	1	0.198694596	1.029512076	0
DBP	2	B	6.736455759	13.75442484	315	0.04659197	0.889035857	0.909420407	0.156069142	0
DBP	2	E	11.34734666	23.45599672	315	0.213912232	1.35E-06	-14.17547141	-0.55540385	1
DBP	2	R	0.098818899	1.649653305	280	0.006932773	1	-0.094179902	-0.4879818	0
DBP	3	B	6.479749088	13.77029665	288	0.03125	0.998679984	0.652713736	0.11201472	0
DBP	3	E	6.150610775	16.37909855	288	0.304534314	2.24E-12	-19.37220729	-0.759015217	1
DBP	3	R	0.899081983	6.41891139	256	0.023897059	0.999999298	0.706083182	3.65848481	0
DBP	4	B	6.571225657	14.38710875	288	0.03125	0.998679984	0.744190305	0.127713367	0
DBP	4	E	4.065695508	12.80641621	288	0.337418301	4.22E-15	-21.45712256	-0.840703503	1
DBP	4	R	0.15131629	2.34467702	256	0.004365809	1	-0.041682511	-0.215972904	0
DBP	5	B	7.462866925	13.75983213	207	0.096618357	0.199263869	1.635831573	0.280731362	0
DBP	5	E	4.429285237	17.54434778	207	0.354930378	6.17E-14	-21.09353283	-0.82645783	1
DBP	5	R	1.460951816	7.73327671	184	0.034367008	0.999487711	1.267953015	6.569745552	0
DBP	10	B	2.918507663	8.104751531	99	0.110814023	0.317300528	-2.908527689	-0.499143649	0
DBP	10	E	2.933851787	11.57518749	99	0.379084967	9.21E-10	-22.58896628	-0.885049849	1
DBP	10	R	0	0	88	0.007352941	1	-0.192998801	-1	0
DBP	20	B	NA	NA	NA	NA	NA	NA	NA	NA
DBP	20	E	NA	NA	NA	NA	NA	NA	NA	NA
DBP	20	R	NA	NA	NA	NA	NA	NA	NA	NA
DEET	0	B	8.139881354	12.81099008	315	0	1	0	0	0
DEET	0	E	18.01814643	28.02890321	315	0	1	0	0	0
DEET	0	R	0.375744029	3.576583051	280	0	1	0	0	0
DEET	1	B	8.341701959	12.94772573	306	0.03426704	0.993253786	0.201820604	0.024794047	0
DEET	1	E	23.65905196	32.52809817	306	0.100560224	0.086625421	5.640905529	0.313068026	0
DEET	1	R	7.98E-05	0.001315839	272	0.028466387	0.999879054	-0.375664245	-0.999787663	0
DEET	2.54	B	10.30039007	14.59809915	324	0.091093474	0.141155012	2.160508718	0.26542263	0
DEET	2.54	E	23.96978859	30.513542	324	0.117107584	0.025030303	5.951642157	0.330313786	0
DEET	2.54	R	0.31708126	3.152693065	288	0.018253968	1	-0.058662769	-0.156124287	0
DEET	6.45	B	8.878421521	13.38219787	306	0.044070962	0.923754053	0.738540167	0.090731073	0
DEET	6.45	E	20.15129922	28.48313752	306	0.056022409	0.714624691	2.13315279	0.118389136	0
DEET	6.45	R	1.052995856	7.296871938	272	0.018697479	1	0.677251827	1.802428712	0
DEET	16.4	B	8.304544063	12.50886555	297	0.042520443	0.945076539	0.164662709	0.020229129	0
DEET	16.4	E	20.60199724	29.17831587	297	0.081096681	0.267141566	2.583850814	0.143402698	0
DEET	16.4	R	0.293544151	4.294571743	264	0.017424242	1	-0.082199878	-0.218765627	0

DEET	35	B	7.261525426	11.93374437	306	0.0643324	0.541714667	-0.878355928	-0.107907707	0
DEET	35	E	14.93098116	23.22216932	306	0.066293184	0.502600222	-3.087165265	-0.171336451	0
DEET	35	R	0	0	272	0.032142857	0.998842782	-0.375744029	-1	0
DEET	74.8	B	7.364222625	11.07558318	297	0.050505051	0.830360377	-0.77565873	-0.095291159	0
DEET	74.8	E	11.78502304	18.72327118	297	0.098701299	0.101729158	-6.233123394	-0.345935883	0
DEET	74.8	R	0.29847627	3.031761008	264	0.013203463	1	-0.077267759	-0.205639354	0
DEET	100	B	7.928034355	11.78998791	315	0.044444444	0.914797537	-0.211847	-0.026025809	0
DEET	100	E	8.018628493	13.77926173	315	0.155555556	0.000978906	-9.999517937	-0.55496929	1
DEET	100	R	7.75E-05	0.001296905	280	0.028571429	0.999847967	-0.375666524	-0.999793729	0
DEHP	0	B	4.355020571	10.63226359	315	0	1	0	0	0
DEHP	0	E	34.09984119	41.62974144	315	0	1	0	0	0
DEHP	0	R	0.538039462	4.331106012	280	0	1	0	0	0
DEHP	1	B	5.806946549	13.81006683	315	0.053968254	0.748621806	1.451925978	0.333391302	0
DEHP	1	E	34.60269152	43.38025555	315	0.041269841	0.951303736	0.502850329	0.014746413	0
DEHP	1	R	0.063708996	1.066055398	280	0.021428571	0.999999954	-0.474330466	-0.881590478	0
DEHP	3	B	6.099497245	13.46994801	324	0.075220459	0.326719911	1.744476674	0.400566805	0
DEHP	3	E	27.95956106	38.71738292	324	0.10952381	0.043341107	-6.140280125	-0.180067704	0
DEHP	3	R	0.990050727	9.391834695	288	0.011111111	1	0.452011265	0.840108016	0
DEHP	5	B	4.169412521	10.87354641	324	0.02292769	0.999996406	-0.18560805	-0.042619328	0
DEHP	5	E	28.08816119	40.79249571	324	0.1170194	0.025195926	-6.011679994	-0.176296422	0
DEHP	5	R	0.227487733	2.76122932	288	0.014484127	1	-0.310551729	-0.577191361	0
DEHP	10	B	4.88770174	12.48722001	324	0.027601411	0.99971608	0.532681169	0.122314272	0
DEHP	10	E	22.90822677	33.85965373	324	0.141798942	0.003247903	-11.19161442	-0.328201365	0
DEHP	10	R	0.386405385	4.298444615	288	0.011111111	1	-0.151634077	-0.281827054	0
DEHP	20	B	5.359263692	12.83367449	288	0.038988095	0.976197859	1.004243121	0.230594346	0
DEHP	20	E	31.44538755	40.89741883	288	0.074404762	0.375537701	-2.654453635	-0.077843578	0
DEHP	20	R	0.31984128	3.707630802	256	0.010379464	1	-0.218198182	-0.405543083	0
DEHP	40	B	3.90349397	10.85458712	315	0.034920635	0.990716312	-0.451526601	-0.103679556	0
DEHP	40	E	22.4847743	34.92801845	315	0.161904762	0.000518798	-11.61506689	-0.340619384	0
DEHP	40	R	0.406436058	4.511521631	280	0.007142857	1	-0.131603404	-0.244598051	0
DEHP	80	B	4.34510049	11.3935715	324	0.022839506	0.99999678	-0.009920081	-0.002277849	0
DEHP	80	E	25.44635184	36.93956159	324	0.128130511	0.010554735	-8.653489349	-0.253769198	0
DEHP	80	R	0.385350362	3.168043687	288	0.010714286	1	-0.1526891	-0.28378792	0
DEP	0	B	6.224893924	11.70942577	306	0	1	0	0	0
DEP	0	E	6.52545242	13.19231784	306	0	1	0	0	0
DEP	0	R	1.01733185	5.812123317	272	0	1	0	0	0
DEP	1	B	8.331658313	13.88786414	324	0.075889615	0.325026439	2.106764388	0.338441813	0
DEP	1	E	9.849818643	19.42377252	324	0.074074074	0.35364751	3.324366223	0.50944609	0
DEP	1	R	0.959533173	4.979614878	288	0.017361111	1	-0.057798677	-0.056813986	0
DEP	2.54	B	10.42872372	16.30062656	324	0.125090777	0.014525409	4.203829793	0.675325531	0

DEP	2.54	E	17.33505458	26.17535746	324	0.21151053	1.53E-06	10.80960216	1.656529151	1
DEP	2.54	R	1.336444168	7.417222008	288	0.013684641	1	0.319112318	0.313675737	0
DEP	6.45	B	10.59135042	16.80205421	324	0.120733479	0.020348948	4.366456492	0.701450747	0
DEP	6.45	E	21.26568658	31.35960879	324	0.219135802	5.46E-07	14.74023416	2.258883096	1
DEP	6.45	R	1.134123627	9.868030742	288	0.016135621	1	0.116791777	0.114802046	0
DEP	16.4	B	10.2919502	17.68468294	324	0.110021786	0.044300426	4.067056278	0.653353507	0
DEP	16.4	E	27.31039454	40.87611438	324	0.293028322	3.66E-12	20.78494212	3.185210892	1
DEP	16.4	R	0.662419877	4.627181596	288	0.027777778	0.999917096	-0.354911973	-0.348865489	0
DEP	35	B	10.67329445	16.24575279	315	0.127917834	0.012444347	4.448400527	0.714614672	0
DEP	35	E	25.09913808	35.88724178	315	0.255555556	3.13E-09	18.57368566	2.846344508	1
DEP	35	R	0.727694217	8.006935028	280	0.0375	0.990144618	-0.289637633	-0.284703199	0
DEP	74.8	B	8.7954694	14.83346867	315	0.099439776	0.092864336	2.570575475	0.412950888	0
DEP	74.8	E	26.69477337	39.90361491	315	0.249112979	8.60E-09	20.16932095	3.09086936	1
DEP	74.8	R	0.313584788	2.9507604	280	0.041071429	0.974085465	-0.703747062	-0.691757623	0
DEP	100	B	7.281820651	13.9253103	324	0.06063907	0.609180227	1.056926726	0.169790319	0
DEP	100	E	26.04816546	38.76652632	324	0.268518519	2.79E-10	19.52271304	2.991779234	1
DEP	100	R	0.620297786	5.952656171	288	0.03125	0.9991886	-0.397034064	-0.390269964	0
DIBP	0	B	7.489457405	15.58252074	324	0	1	0	0	0
DIBP	0	E	17.66473102	29.87175984	324	0	1	0	0	0
DIBP	0	R	0.613214852	5.343170033	288	0	1	0	0	0
DIBP	2	B	6.366429713	13.8790918	315	0.042945326	0.929918724	-1.123027692	-0.149947804	0
DIBP	2	E	6.40459622	16.10499269	315	0.216666667	6.14E-07	-11.2601348	-0.637435961	1
DIBP	2	R	0.529203837	4.533406675	280	0.014781746	1	-0.084011015	-0.137000946	0
DIBP	4	B	6.499393651	14.80101248	315	0.046119929	0.886128892	-0.990063754	-0.132194323	0
DIBP	4	E	3.763020757	11.84310781	315	0.276984127	4.55E-11	-13.90171027	-0.786975485	1
DIBP	4	R	0.346137164	4.392596533	280	0.006845238	1	-0.267077688	-0.435536887	0
DIBP	5	B	5.417900412	12.4363934	306	0.06989833	0.425463317	-2.071556994	-0.276596405	0
DIBP	5	E	2.705723453	8.865906707	306	0.289215686	7.37E-12	-14.95900757	-0.84682906	1
DIBP	5	R	1.211448024	6.712639275	272	0.05249183	0.835547834	0.598233172	0.975568629	0
DIBP	6	B	5.633749437	12.56281095	315	0.069400353	0.425143955	-1.855707968	-0.247776023	0
DIBP	6	E	1.860807517	7.859836597	315	0.334126984	6.66E-16	-15.80392351	-0.894659731	1
DIBP	6	R	0.861390271	5.129402437	280	0.039781746	0.978189912	0.248175419	0.404712017	0
DIBP	8	B	5.575372853	12.75371305	288	0.065972222	0.520552213	-1.914084553	-0.255570524	0
DIBP	8	E	1.778609676	7.027123192	288	0.326388889	1.55E-14	-15.88612135	-0.899312949	1
DIBP	8	R	0.3612093	2.839406096	256	0.010416667	1	-0.252005552	-0.410958005	0
DIBP	10	B	6.546499226	14.34976872	252	0.049382716	0.879831288	-0.94295818	-0.125904739	0
DIBP	10	E	2.073946827	9.187858299	252	0.337301587	1.97E-14	-15.5907842	-0.88259392	1
DIBP	10	R	1.157633596	6.329110891	224	0.030753968	0.99976823	0.544418745	0.887810762	0
DIBP	15	B	2.49847303	8.909872439	162	0.145061728	0.021234639	-4.990984375	-0.66640133	0
DIBP	15	E	1.126194842	6.089401696	162	0.342592593	1.95E-11	-16.53853618	-0.936246137	1

DIBP	15	R	0.275637823	2.434819699	144	0.013888889	1	-0.337577028	-0.550503673	0
DNP	0	B	6.954625356	16.65737162	324	0	1	0	0	0
DNP	0	E	31.05575954	46.98938901	324	0	1	0	0	0
DNP	0	R	1.294623449	8.546829333	288	0	1	0	0	0
DNP	2	B	6.784764715	14.76568337	324	0.024691358	0.999970014	-0.169860641	-0.024424125	0
DNP	2	E	34.19482487	49.88465637	324	0.067901235	0.443946011	3.139065325	0.101078363	0
DNP	2	R	0.581718055	5.112207658	288	0.020833333	0.999999973	-0.712905394	-0.550666215	0
DNP	10	B	7.820215968	17.73733267	315	0.041446208	0.946657439	0.865590613	0.12446258	0
DNP	10	E	25.88369477	40.69274991	315	0.060934744	0.593475565	-5.172064769	-0.166541242	0
DNP	10	R	0.225306989	3.081891369	280	0.03452381	0.995845176	-1.06931646	-0.825967165	0
DNP	30	B	8.518799156	18.70310647	324	0.058641975	0.633246263	1.5641738	0.224911296	0
DNP	30	E	34.31485372	49.49436726	324	0.067901235	0.443946011	3.259094179	0.104943309	0
DNP	30	R	0.70175362	5.612251495	288	0.020833333	0.999999973	-0.592869829	-0.457947698	0
DNP	50	B	6.591127448	15.08274537	324	0.027777778	0.999633292	-0.363497907	-0.052267072	0
DNP	50	E	31.00873889	47.85589085	324	0.033950617	0.992163294	-0.047020651	-0.001514072	0
DNP	50	R	0.476526413	3.877282105	288	0.020833333	0.999999973	-0.818097036	-0.631918908	0
DNP	70	B	8.044670629	16.59843992	315	0.054144621	0.7372234	1.090045274	0.156736735	0
DNP	70	E	29.1116895	43.51693211	315	0.033597884	0.993700176	-1.944070047	-0.06259934	0
DNP	70	R	0.010308156	0.169895199	280	0.041369048	0.968293406	-1.284315293	-0.992037719	0
DNP	80	B	6.127921323	14.80314582	315	0.041446208	0.946657439	-0.826704032	-0.118871109	0
DNP	80	E	30.99109926	46.47630107	315	0.035097002	0.989313919	-0.064660278	-0.00208207	0
DNP	80	R	1.125759231	8.296385409	280	0.016865079	1	-0.168864218	-0.130435007	0
DNP	100	B	6.287615451	14.0447598	315	0.030599647	0.998305551	-0.667009904	-0.095908819	0
DNP	100	E	25.26730515	41.91318835	315	0.07037037	0.40762035	-5.78845439	-0.186389078	0
DNP	100	R	0.418526728	4.783644842	280	0.037797619	0.987295749	-0.876096721	-0.676719336	0
G14 Mix	0	B	6.058530694	15.69890937	288	0	1	0	0	0
G14 Mix	0	E	25.00783551	42.93051055	288	0	1	0	0	0
G14 Mix	0	R	1.010131944	7.462326778	256	0	1	0	0	0
G14 Mix	28	B	3.38998145	10.01453143	252	0.060515873	0.708625586	-2.668549244	-0.440461455	0
G14 Mix	28	E	1.630618156	7.745938403	252	0.314484127	5.70E-12	-23.37721735	-0.93479571	1
G14 Mix	28	R	1.306152313	6.328319453	224	0.044642857	0.971133153	0.296020369	0.293051191	0
G14 Mix	56	B	0.170810873	2.029364426	279	0.207997312	9.46E-06	-5.887719821	-0.971806552	1
G14 Mix	56	E	0.127563693	1.685642989	279	0.374775986	0	-24.88027181	-0.994899051	1
G14 Mix	56	R	0.000700044	0.003842055	248	0.03125	0.999684746	-1.0094319	-0.999306977	0
G14 Mix	63	B	0.000629025	0.003649574	207	0.21875	1.97E-05	-6.057901669	-0.999896175	1
G14 Mix	63	E	0.0008387	0.004193146	207	0.381944444	1.11E-15	-25.00699681	-0.999966462	1
G14 Mix	63	R	0.000825596	0.004162828	184	0.03125	0.999941857	-1.009306348	-0.999182685	0
G14 Mix	70	B	0.064522082	0.612272011	261	0.207255747	1.56E-05	-5.994008612	-0.98935021	1
G14 Mix	70	E	0.000415735	0.002980474	261	0.381944444	0	-25.00741977	-0.999983376	1
G14 Mix	70	R	0.001590187	0.008773331	232	0.03125	0.999774283	-1.008541757	-0.998425763	0

G14 Mix	77	B	0.000344466	0.002717728	252	0.21875	5.19E-06	-6.058186228	-0.999943144	1
G14 Mix	77	E	0.032638205	0.507149727	252	0.37797619	0	-24.9751973	-0.998694881	1
G14 Mix	77	R	0.000193762	0.002045984	224	0.045758929	0.963850002	-1.009938182	-0.999808181	0
G14 Mix	84	B	0.000209675	0.002127945	207	0.219504831	1.82E-05	-6.058321019	-0.999965392	1
G14 Mix	84	E	0.00041935	0.002994653	207	0.381944444	1.11E-15	-25.00741616	-0.999983231	1
G14 Mix	84	R	0.000353827	0.002755832	184	0.038383152	0.9974704	-1.009778117	-0.999649722	0
G14 Mix	112	B	NA	NA	NA	NA	NA	NA	NA	NA
G14 Mix	112	E	NA	NA	NA	NA	NA	NA	NA	NA
G14 Mix	112	R	NA	NA	NA	NA	NA	NA	NA	NA
HHCB	0	B	4.387297772	12.29367037	324	0	1	0	0	0
HHCB	0	E	26.93483813	42.39769681	324	0	1	0	0	0
HHCB	0	R	0.273151258	2.814478057	288	0	1	0	0	0
HHCB	10	B	3.024262884	8.896521235	324	0.049382716	0.824393461	-1.363034888	-0.310677542	0
HHCB	10	E	0.883929859	6.522039638	324	0.419753086	0	-26.05090827	-0.967182656	1
HHCB	10	R	0.332830036	2.945740482	288	0.006944444	1	0.059678778	0.218482531	0
HHCB	14	B	0.264100521	2.280949652	324	0.138888889	0.003860908	-4.123197251	-0.939803374	1
HHCB	14	E	0.200000958	1.730769507	324	0.447530864	0	-26.73483717	-0.992574637	1
HHCB	14	R	7.54E-05	0.001278766	288	0.013888889	1	-0.273075906	-0.999724138	0
HHCB	16	B	0.01011392	0.126670071	324	0.163580247	0.000343403	-4.377183852	-0.997694727	1
HHCB	16	E	6.70E-05	0.001205632	324	0.472222222	0	-26.93477115	-0.999997513	1
HHCB	16	R	0	0	288	0.013888889	1	-0.273151258	-1	0
HHCB	20	B	0	0	306	0.172839506	0.00016504	-4.387297772	-1	1
HHCB	20	E	0	0	306	0.475308642	0	-26.93483813	-1	1
HHCB	20	R	0.004866857	0.078956534	272	0.013888889	1	-0.268284401	-0.982182557	0
HHCB	24	B	6.70E-05	0.001205632	324	0.172839506	0.000125167	-4.387230793	-0.999984733	1
HHCB	24	E	0.000133959	0.00170238	324	0.472222222	0	-26.93470417	-0.999995027	1
HHCB	24	R	0	0	288	0.013888889	1	-0.273151258	-1	0
HHCB	28	B	6.70E-05	0.001205632	324	0.172839506	0.000125167	-4.387230793	-0.999984733	1
HHCB	28	E	6.70E-05	0.001205632	324	0.472222222	0	-26.93477115	-0.999997513	1
HHCB	28	R	0	0	288	0.013888889	1	-0.273151258	-1	0
HHCB	32	B	0	0	315	0.172839506	0.000143448	-4.387297772	-1	1
HHCB	32	E	6.89E-05	0.001222734	315	0.472222222	0	-26.93476924	-0.999997442	1
HHCB	32	R	0	0	280	0.013888889	1	-0.273151258	-1	0
Lilial	0	B	2.559523743	10.18210875	315	0	1	0	0	0
Lilial	0	E	27.15112334	44.48747661	315	0	1	0	0	0
Lilial	0	R	0.02968439	0.366707095	280	0	1	0	0	0
Lilial	20	B	4.548542642	11.72792821	315	0.082539683	0.233520535	1.989018899	0.777105079	0
Lilial	20	E	20.70684602	36.63073681	315	0.073015873	0.370570972	-6.444277319	-0.23734846	0
Lilial	20	R	0.982452824	5.389768451	280	0.039285714	0.982130802	0.952768435	32.09661545	0
Lilial	40	B	4.344755571	11.31260253	315	0.111111111	0.0409358	1.785231829	0.697485942	0

Lilial	40	E	12.96936934	28.18122428	315	0.161904762	0.000518798	-14.181754	-0.522326602	1
Lilial	40	R	0.402948397	4.757717118	280	0.010714286	1	0.373264008	12.57442089	0
Lilial	60	B	4.160948609	11.89671587	315	0.095238095	0.114843478	1.601424866	0.625672987	0
Lilial	60	E	5.998057871	15.95591027	315	0.250793651	4.97E-09	-21.15306547	-0.779086199	1
Lilial	60	R	1.274259121	7.531775314	280	0.046428571	0.923467842	1.244574732	41.92691015	0
Lilial	70	B	3.473186583	9.310588353	315	0.104761905	0.063037405	0.91366284	0.356965956	0
Lilial	70	E	3.607321846	9.832932985	315	0.273015873	1.27E-10	-23.54380149	-0.867139131	1
Lilial	70	R	1.004774365	5.445219293	280	0.039285714	0.982130802	0.975089976	32.84857771	0
Lilial	80	B	2.913277413	8.411562491	324	0.099029982	0.087193051	0.353753671	0.13821074	0
Lilial	80	E	4.788840542	14.20304921	324	0.25952381	9.06E-10	-22.3622828	-0.823622747	1
Lilial	80	R	1.152434243	5.605601028	288	0.058829365	0.709631041	1.122749853	37.82290529	0
Lilial	90	B	2.290300273	7.076234316	324	0.071252205	0.392066906	-0.26922347	-0.105184986	0
Lilial	90	E	2.660295357	8.444498455	324	0.324867725	4.55E-15	-24.49082798	-0.902018958	1
Lilial	90	R	1.698284127	6.984016489	288	0.083134921	0.280277991	1.668599738	56.21135437	0
Lilial	100	B	2.717013693	8.074467319	315	0.104761905	0.063037405	0.15748995	0.061530959	0
Lilial	100	E	2.166074055	6.889490159	315	0.333333333	1.22E-15	-24.98504928	-0.920221568	1
Lilial	100	R	0.827055379	4.085338355	280	0.042857143	0.959226548	0.797370989	26.86162667	0
TPP	0	B	5.557163508	14.25877092	324	0	1	0	0	0
TPP	0	E	28.64435944	42.17088029	324	0	1	0	0	0
TPP	0	R	0.221911817	1.801217925	288	0	1	0	0	0
TPP	2	B	6.37324226	14.90097536	324	0.070987654	0.387885061	0.816078752	0.146851672	0
TPP	2	E	11.25813135	23.44326928	324	0.188271605	2.06E-05	-17.38622808	-0.606968647	1
TPP	2	R	1.550971522	7.832007799	288	0.045138889	0.930938569	1.329059706	5.989134443	0
TPP	3	B	5.881410996	14.19891814	324	0.040123457	0.956694152	0.324247488	0.05834766	0
TPP	3	E	7.714106539	19.45113243	324	0.268518519	1.43E-10	-20.9302529	-0.730693697	1
TPP	3	R	2.116488123	8.052692597	288	0.076388889	0.370138738	1.894576306	8.537518791	0
TPP	4	B	5.695676468	15.77091854	324	0.024691358	0.999970014	0.13851296	0.024925119	0
TPP	4	E	6.752882695	17.26979551	324	0.287037037	5.10E-12	-21.89147674	-0.764250874	1
TPP	4	R	1.195761715	6.137194895	288	0.041666667	0.963945244	0.973849898	4.388454441	0
TPP	5	B	5.827157765	14.16196656	324	0.027777778	0.999633292	0.269994257	0.048584904	0
TPP	5	E	5.826287334	14.56634161	324	0.283950617	9.03E-12	-22.8180721	-0.79659914	1
TPP	5	R	2.091320816	8.15236693	288	0.069444444	0.490980372	1.869408999	8.424107492	0
TPP	6	B	4.192552173	10.94426153	306	0.062636166	0.567480787	-1.364611336	-0.245558968	0
TPP	6	E	4.565731119	11.49571351	306	0.288307916	8.69E-12	-24.07862832	-0.840606276	1
TPP	6	R	1.812784037	7.211206103	272	0.085784314	0.254677768	1.59087222	7.168938738	0
TPP	8	B	2.003619516	8.306831067	306	0.099854757	0.086706478	-3.553543993	-0.639452841	0
TPP	8	E	1.922629743	8.900912404	306	0.391067538	0	-26.72172969	-0.93287929	1
TPP	8	R	0.291133808	1.817753011	272	0.030637255	0.999425379	0.069221991	0.311934678	0
TPP	10	B	0.247809214	1.932511738	315	0.150352734	0.001461899	-5.309354294	-0.955407248	1
TPP	10	E	0.330963402	3.00714304	315	0.452733686	0	-28.31339603	-0.988445774	1

TPP	10	R	0.231739994	3.616285063	280	0.013789683	1	0.009828177	0.044288661	0
ZF BMC Mix	0	B	3.851929134	11.90212155	324	0.043992349	0.699104976	0	0	0
ZF BMC Mix	0	E	21.0463946	40.46656245	324	0.041644931	0.761176238	0	0	0
ZF BMC Mix	0	R	0.364251947	3.60993859	288	0.007286776	1	0	0	0
ZF BMC Mix	0.511	B	5.395541499	14.17186479	324	0.01551904	0.999999975	1.543612365	0.400737478	0
ZF BMC Mix	0.511	E	27.50778375	44.34278232	324	0.113980177	0.002423073	6.46138915	0.307006937	0
ZF BMC Mix	0.511	R	0.441562766	4.362423954	288	0.006602113	1	0.077310819	0.212245452	0
ZF BMC Mix	1.02	B	5.637605279	15.74378088	324	0.017170927	0.999999154	1.785676144	0.463579698	0
ZF BMC Mix	1.02	E	17.28388291	35.71031509	324	0.016431925	0.9999998	-3.762511695	-0.178772268	0
ZF BMC Mix	1.02	R	0.733401342	6.457360655	288	0.010758998	1	0.369149395	1.013445222	0
ZF BMC Mix	2.041	B	6.132060127	15.65624875	306	0.034014545	0.937560659	2.280130993	0.591945208	0
ZF BMC Mix	2.041	E	6.524600862	18.45312818	306	0.134539262	0.000262689	-14.52179374	-0.689989617	1
ZF BMC Mix	2.041	R	0.349855131	3.34696133	272	0.012116819	1	-0.014396816	-0.039524334	0
ZF BMC Mix	4.08	B	5.8300391	15.76952618	324	0.022517823	0.999434807	1.978109965	0.513537476	0
ZF BMC Mix	4.08	E	4.139807835	13.70227147	324	0.207529125	4.28E-10	-16.90658677	-0.803300854	1
ZF BMC Mix	4.08	R	1.024034415	8.176518008	288	0.010416667	1	0.659782468	1.811335459	0
ZF BMC Mix	6.13	B	4.061465899	11.91042203	315	0.028839705	0.984558601	0.209536764	0.054397876	0
ZF BMC Mix	6.13	E	3.061549481	10.42933712	315	0.200827185	2.81E-09	-17.98484512	-0.854533304	1
ZF BMC Mix	6.13	R	0.694521736	4.399647832	280	0.027892354	0.994847474	0.330269789	0.906706996	0
ZF BMC Mix	8.16	B	5.750794927	15.09252687	297	0.023924693	0.999118742	1.898865793	0.492964882	0
ZF BMC Mix	8.16	E	3.181628374	12.26402413	297	0.212832551	6.59E-10	-17.86476623	-0.848827867	1
ZF BMC Mix	8.16	R	1.798585003	10.16035708	264	0.037798762	0.919537365	1.434333056	3.937749867	0
ZF BMC Mix	10.2	B	3.770254698	12.67555672	270	0.060198226	0.394442943	-0.081674436	-0.021203515	0
ZF BMC Mix	10.2	E	1.952642724	9.486176482	270	0.262702139	8.69E-14	-19.09375188	-0.907221985	1
ZF BMC Mix	10.2	R	0.75900613	5.830946654	240	0.009389671	1	0.394754183	1.08373939	0
ZF Equi-Mix	0	B	3.756963935	11.99657045	306	0.048688074	0.716072931	0	0	0
ZF Equi-Mix	0	E	27.04234137	49.84618571	306	0.082883395	0.119613097	0	0	0
ZF Equi-Mix	0	R	0.644818384	6.796971819	272	0.034153879	0.983609603	0	0	0
ZF Equi-Mix	1.5	B	7.819801861	18.13592684	315	0.047296986	0.738090926	4.062837926	1.081415205	0
ZF Equi-Mix	1.5	E	9.862626447	24.10238216	315	0.080515298	0.133081017	-17.17971492	-0.635289478	0
ZF Equi-Mix	1.5	R	2.734142259	11.68984201	280	0.033178054	0.98670622	2.089323875	3.240174173	0
ZF Equi-Mix	3	B	6.308004211	16.34828265	324	0.026301664	0.998495687	2.551040276	0.67901644	0
ZF Equi-Mix	3	E	7.464742196	20.57570634	324	0.111379495	0.010158715	-19.57759917	-0.723960951	0
ZF Equi-Mix	3	R	2.290551424	11.16003997	288	0.027626812	0.998711653	1.64573304	2.55224274	0
ZF Equi-Mix	3.6	B	7.509149541	18.7198071	324	0.032742888	0.976414647	3.752185606	0.998728141	0
ZF Equi-Mix	3.6	E	7.778943898	19.56035578	324	0.106011809	0.016696948	-19.26339747	-0.712342072	0
ZF Equi-Mix	3.6	R	2.536651233	12.18459298	288	0.034269324	0.979344898	1.891832849	2.933900297	0
ZF Equi-Mix	4.2	B	6.663398179	17.55174606	324	0.025630703	0.999010047	2.906434244	0.773612495	0
ZF Equi-Mix	4.2	E	6.451542062	17.31071545	324	0.125603865	0.002417877	-20.5907993	-0.761428126	1
ZF Equi-Mix	4.2	R	3.191309868	11.69697069	288	0.053894928	0.641596766	2.546491484	3.949160797	0

ZF Equi-Mix	5.1	B	6.444004154	17.95542182	315	0.035656775	0.953177736	2.687040219	0.715215867	0
ZF Equi-Mix	5.1	E	6.704489512	17.85796532	315	0.115343915	0.007690569	-20.33785185	-0.75207437	1
ZF Equi-Mix	5.1	R	2.865125826	10.43163115	280	0.052898551	0.67606478	2.220307442	3.443306668	0
ZF Equi-Mix	6	B	4.199666737	14.59895158	315	0.069289165	0.268204439	0.442702802	0.117835255	0
ZF Equi-Mix	6	E	3.817998058	13.75469725	315	0.186979526	9.01E-07	-23.22434331	-0.858814072	1
ZF Equi-Mix	6	R	1.094447339	7.085309108	280	0.018426501	0.999999968	0.449628955	0.697295495	0
ZF Equi-Mix	7.5	B	4.095533888	14.27988813	306	0.06299138	0.390146272	0.338569953	0.090117967	0
ZF Equi-Mix	7.5	E	4.38467326	14.02419189	306	0.160935872	4.89E-05	-22.65766811	-0.837858963	1
ZF Equi-Mix	7.5	R	2.248726883	10.7670485	272	0.028239557	0.998648754	1.603908498	2.487380226	0

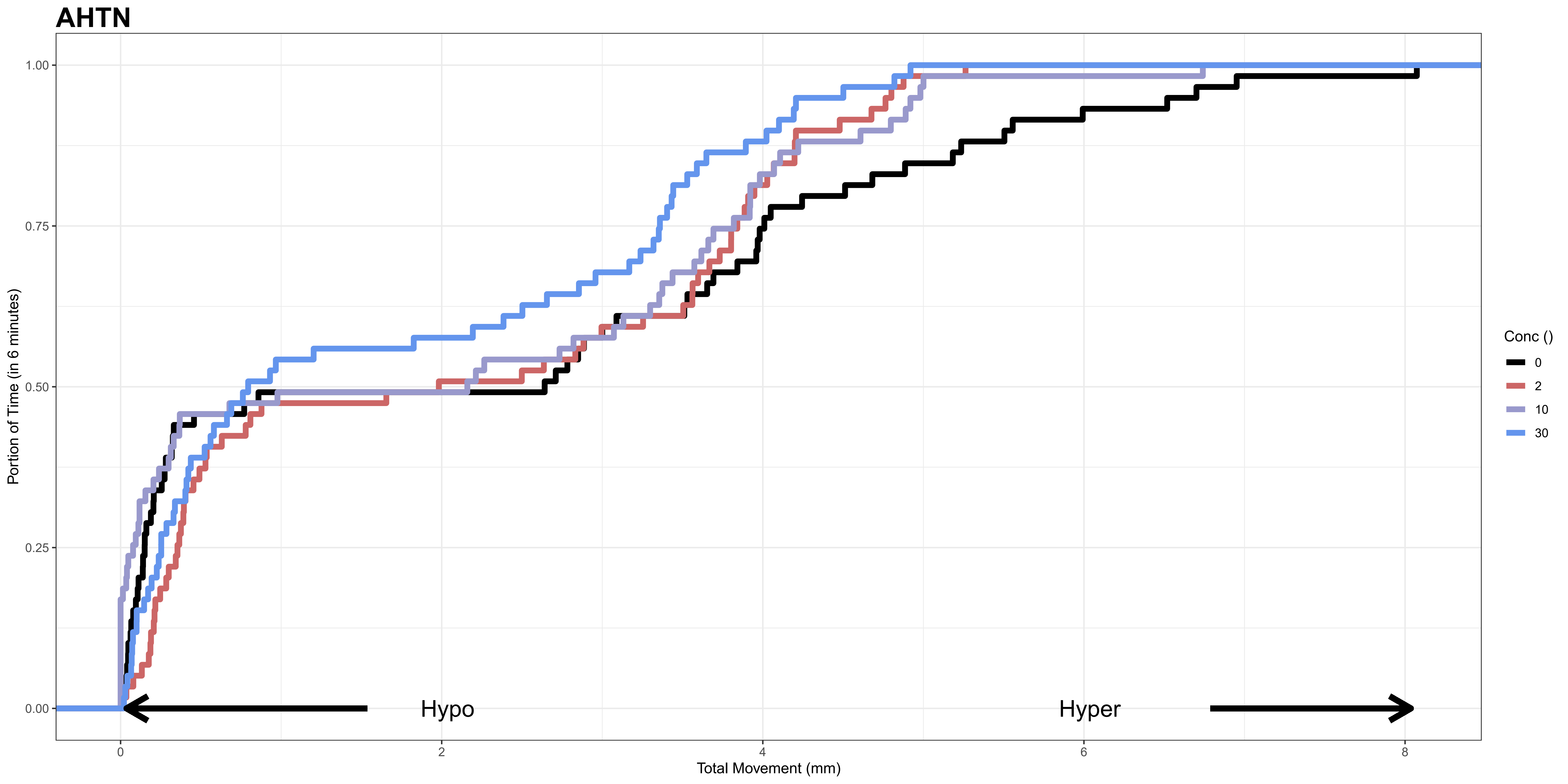

AHTN
Portion of Time (in 6 minutes)
1.00
0.75
0.50
0.25
0.00
Hypo
Hyper
0
2
4
6
8
Total Movement (mm)
Conc ()
0
2
10
30

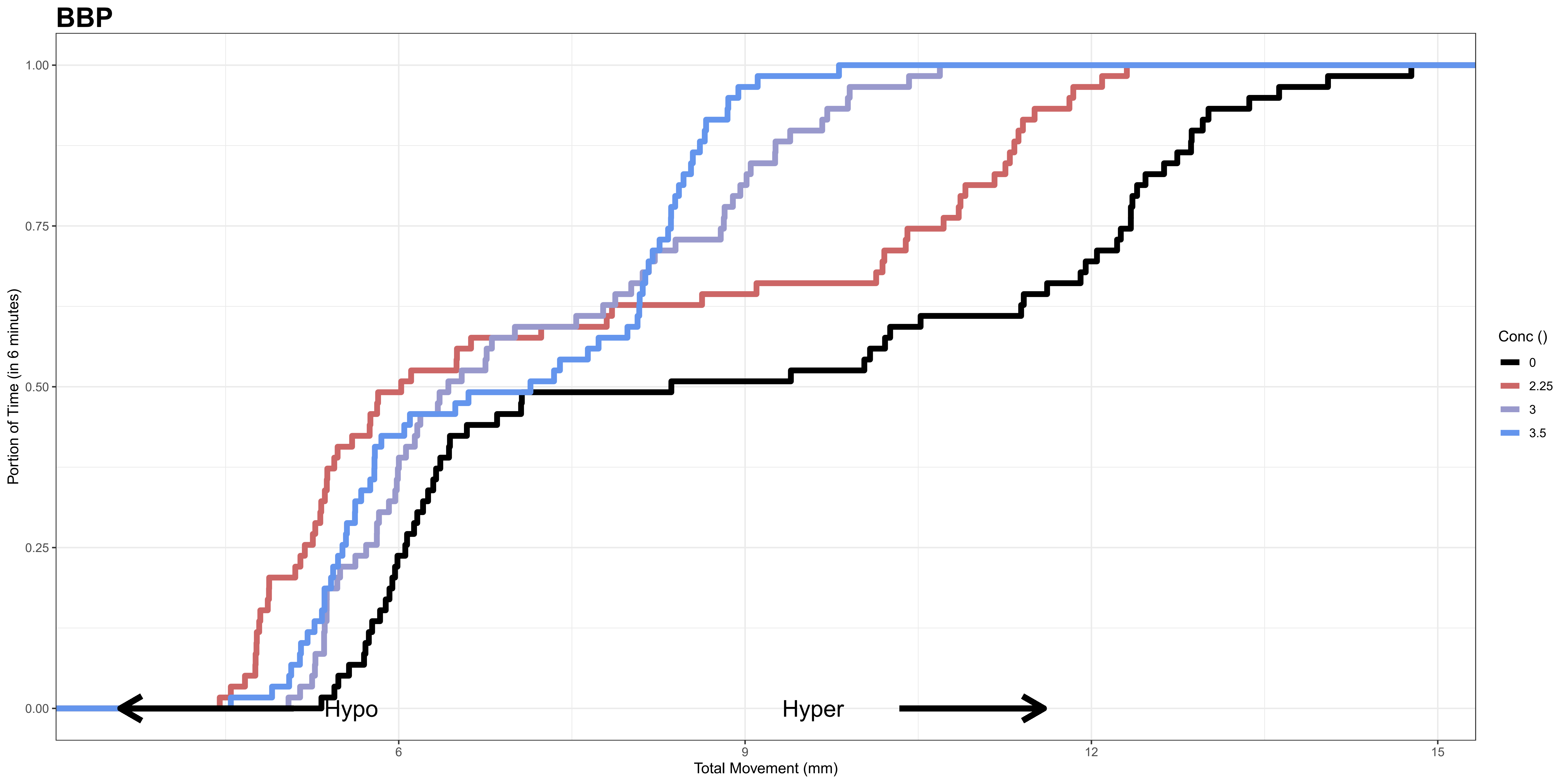
BBP
Portion of Time (in 6 minutes)
1.00
0.75
0.50
0.25
0.00
Total Movement (mm)
6
9
12
15
Hypo
Hyper
Conc ()
0
2.25
3
3.5

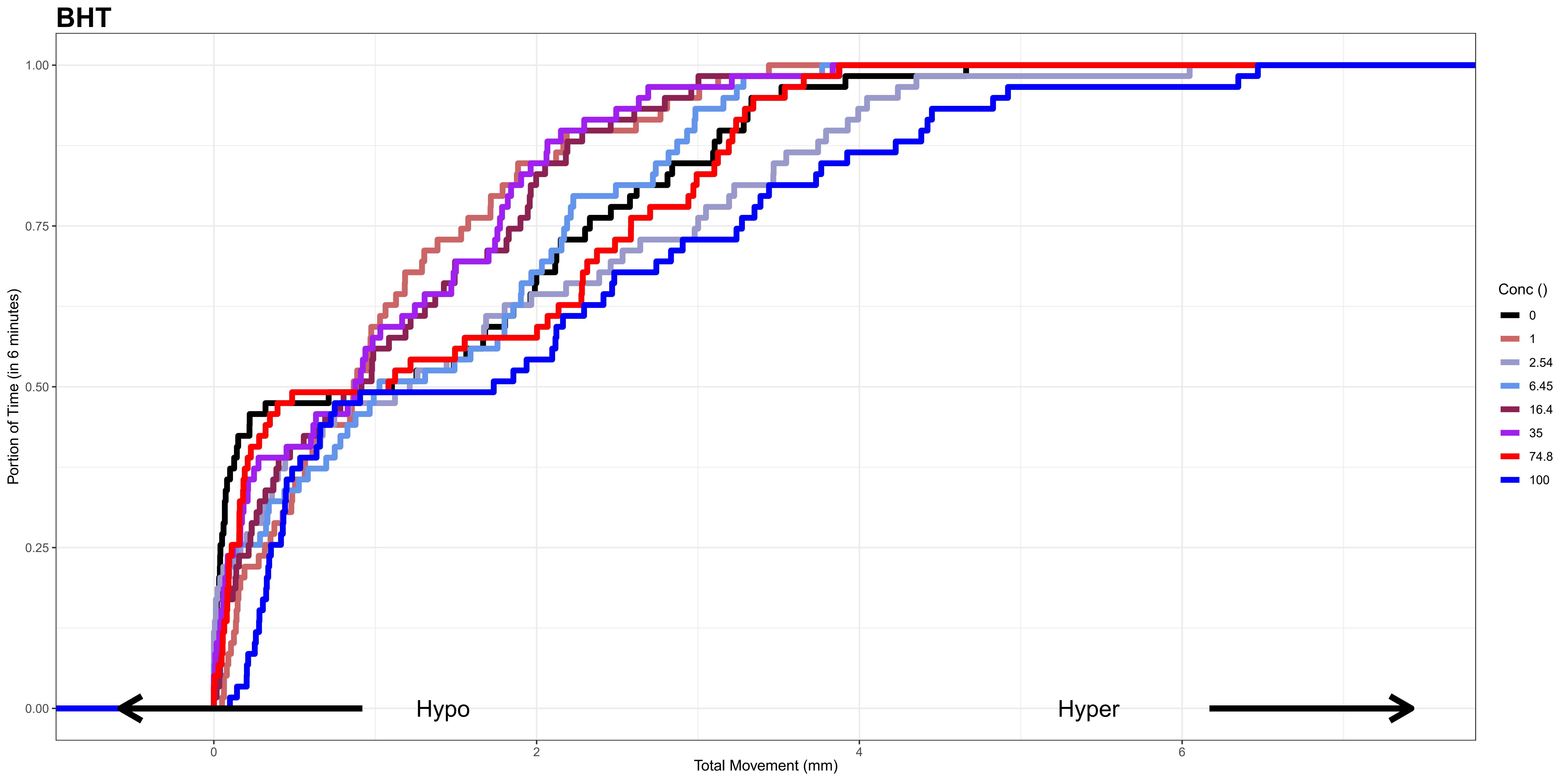
BHT
Portion of Time (in 6 minutes)
1.00
0.75
0.50
0.25
0.00
0
2
4
6
Total Movement (mm)
Hypo
Hyper
Conc ()
0
1
2.54
6.45
16.4
35
74.8
100

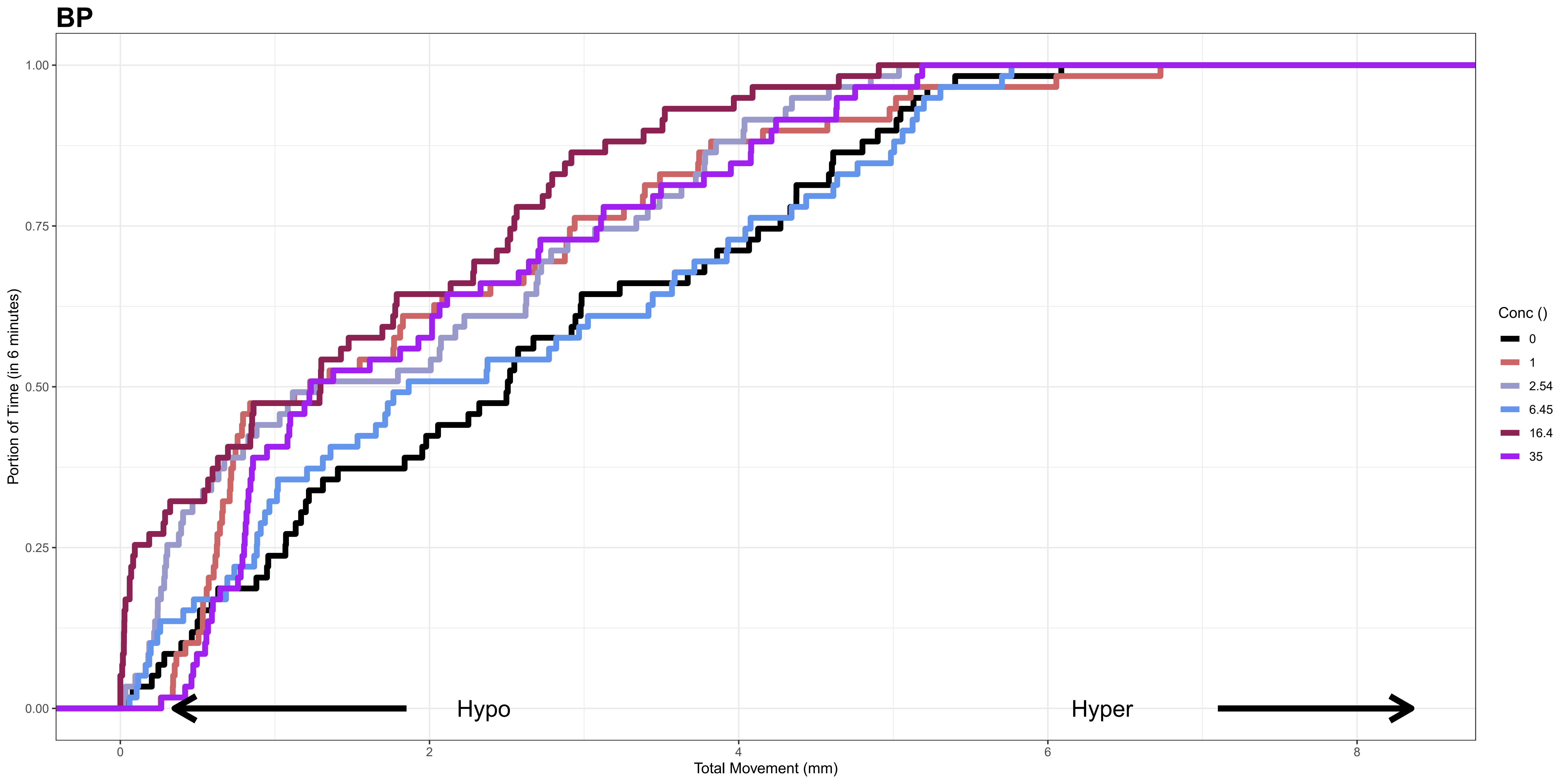
BP
Portion of Time (in 6 minutes)
1.00
0.75
0.50
0.25
0.00
0
2
4
6
8
Total Movement (mm)
Hypo
Hyper
Conc ()
0
1
2.54
6.45
16.4
35

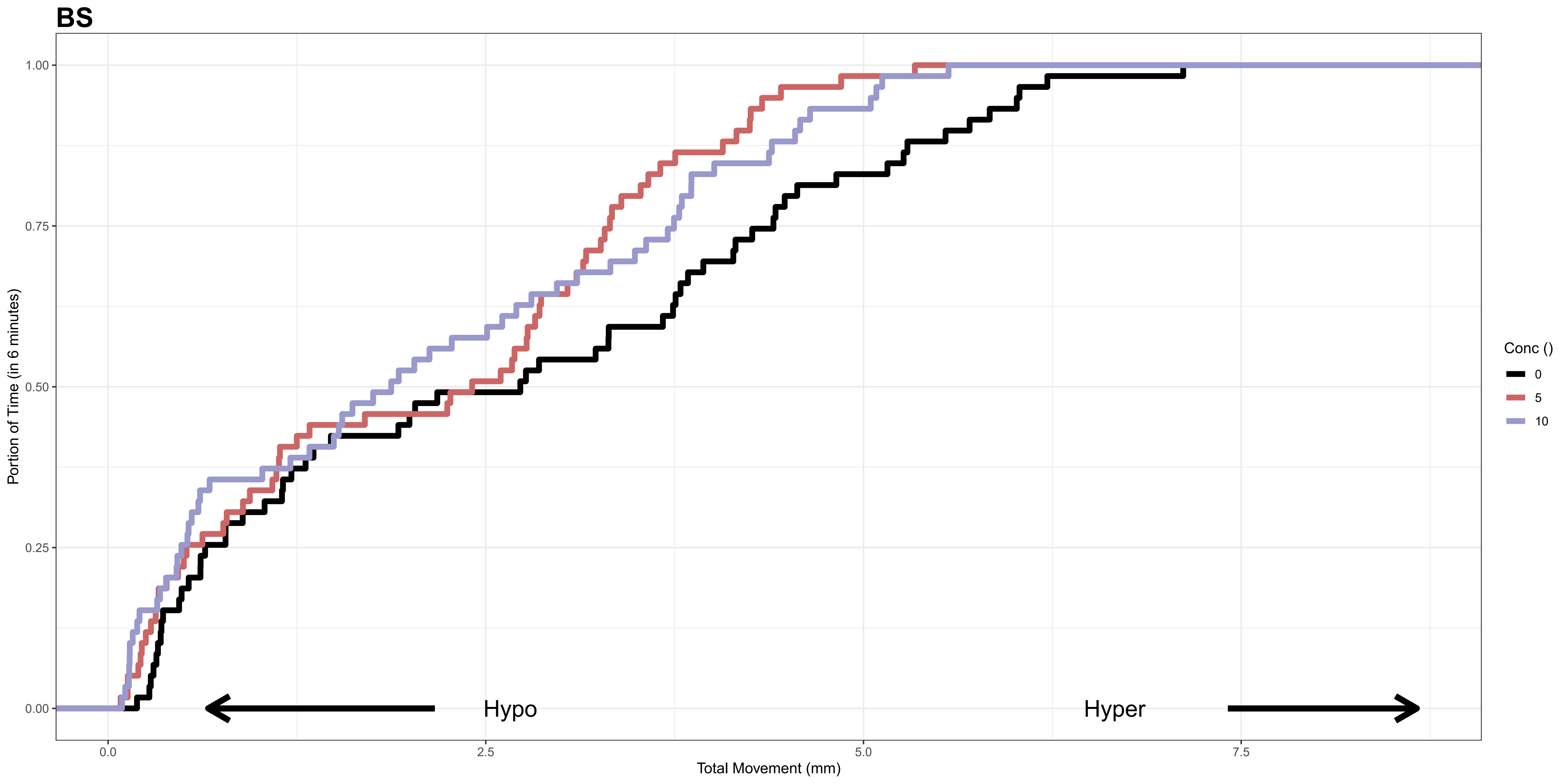
BS
1.00
0.75
0.50
0.25
0.00
Portion of Time (in 6 minutes)
0.0
2.5
5.0
7.5
Total Movement (mm)
Hypo
Hyper
Conc ()
0
5
10

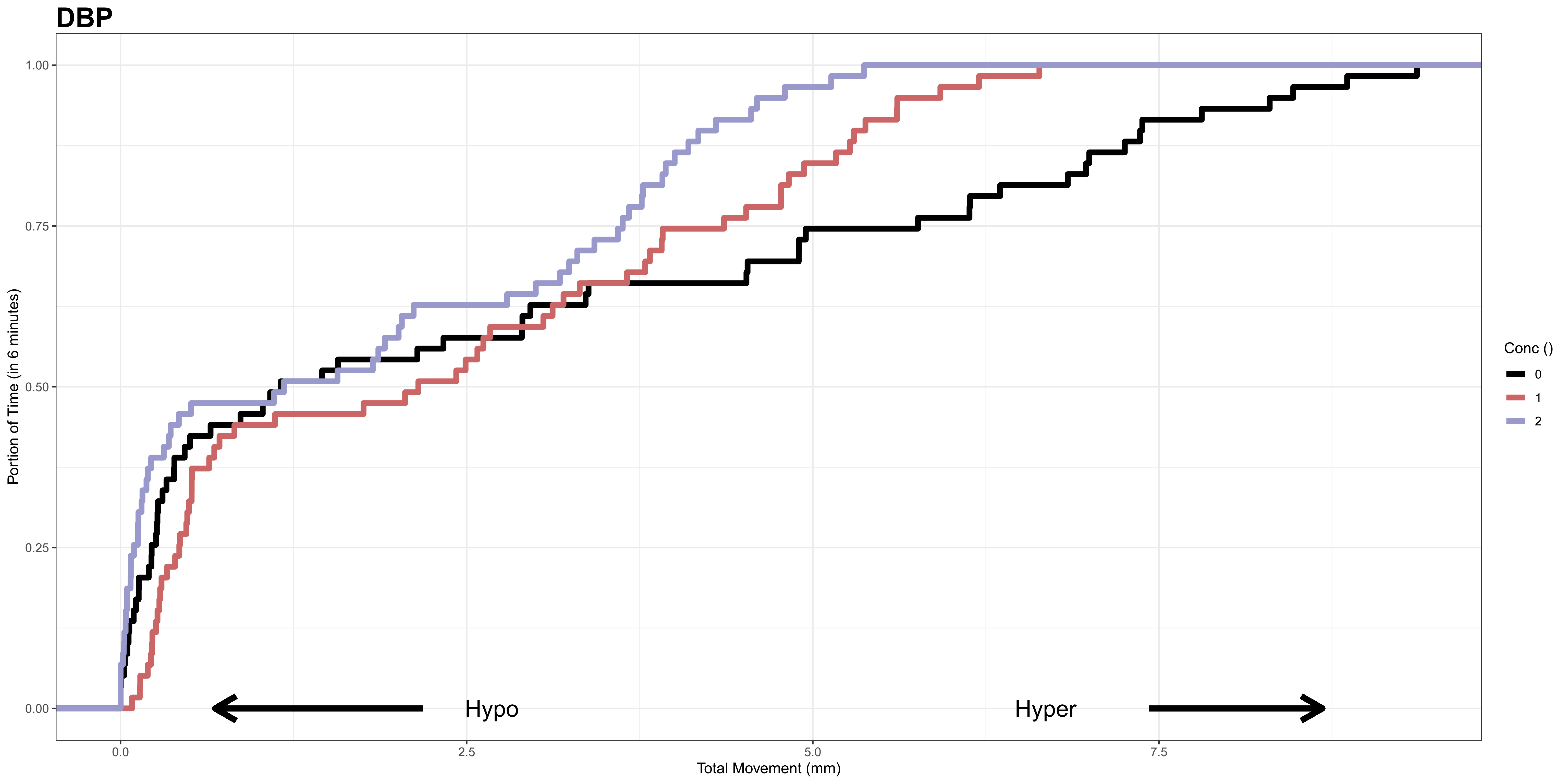

DBP
Portion of Time (in 6 minutes)
1.00
0.75
0.50
0.25
0.00
0.0
2.5
5.0
7.5
Total Movement (mm)
Hypo
Hyper
Conc ()
0
1
2

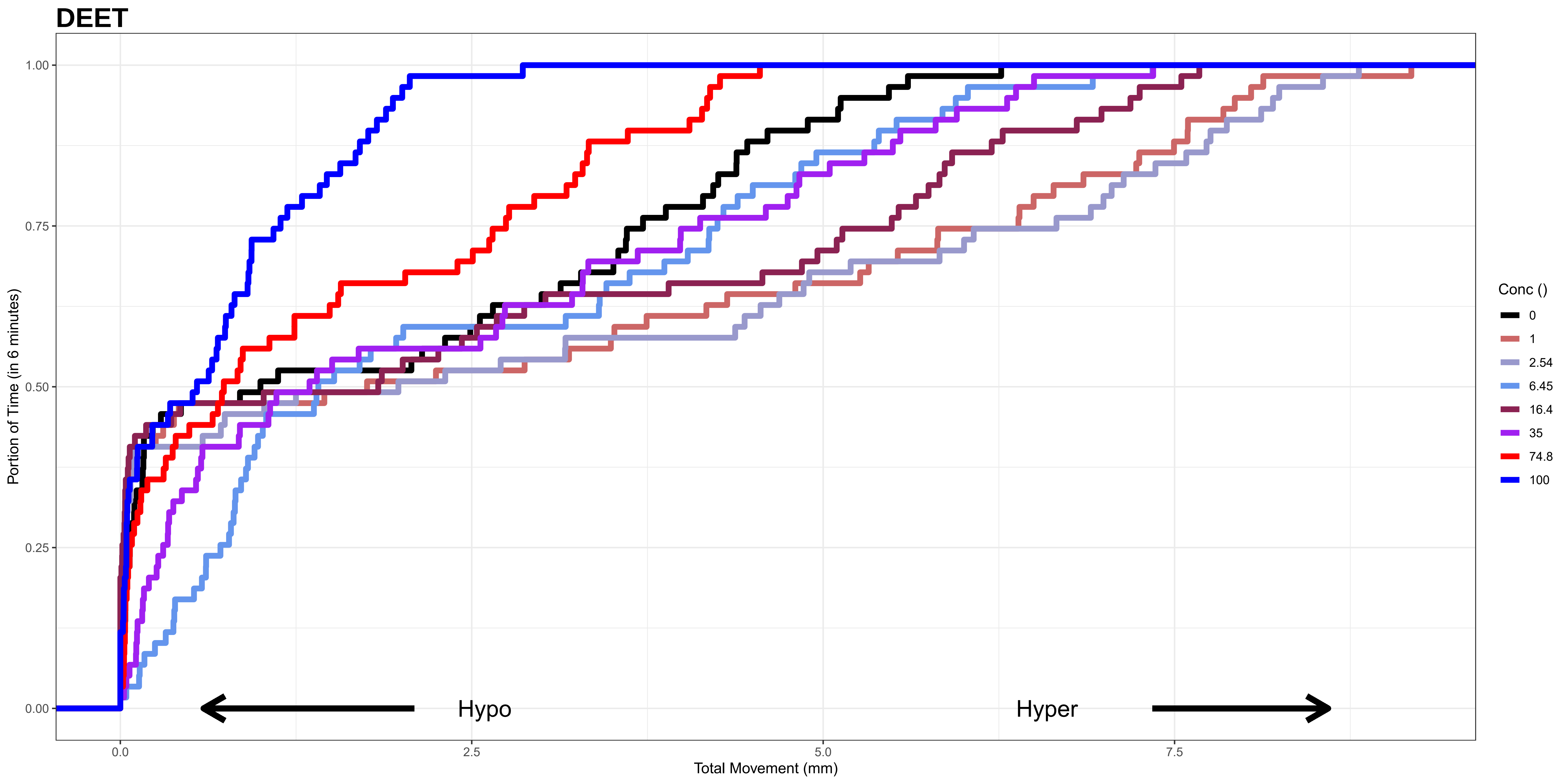
DEET
Portion of Time (in 6 minutes)
1.00
0.75
0.50
0.25
0.00
0.0
2.5
5.0
7.5
Total Movement (mm)
Hypo
Hyper
Conc ()
0
1
2.54
6.45
16.4
35
74.8
100

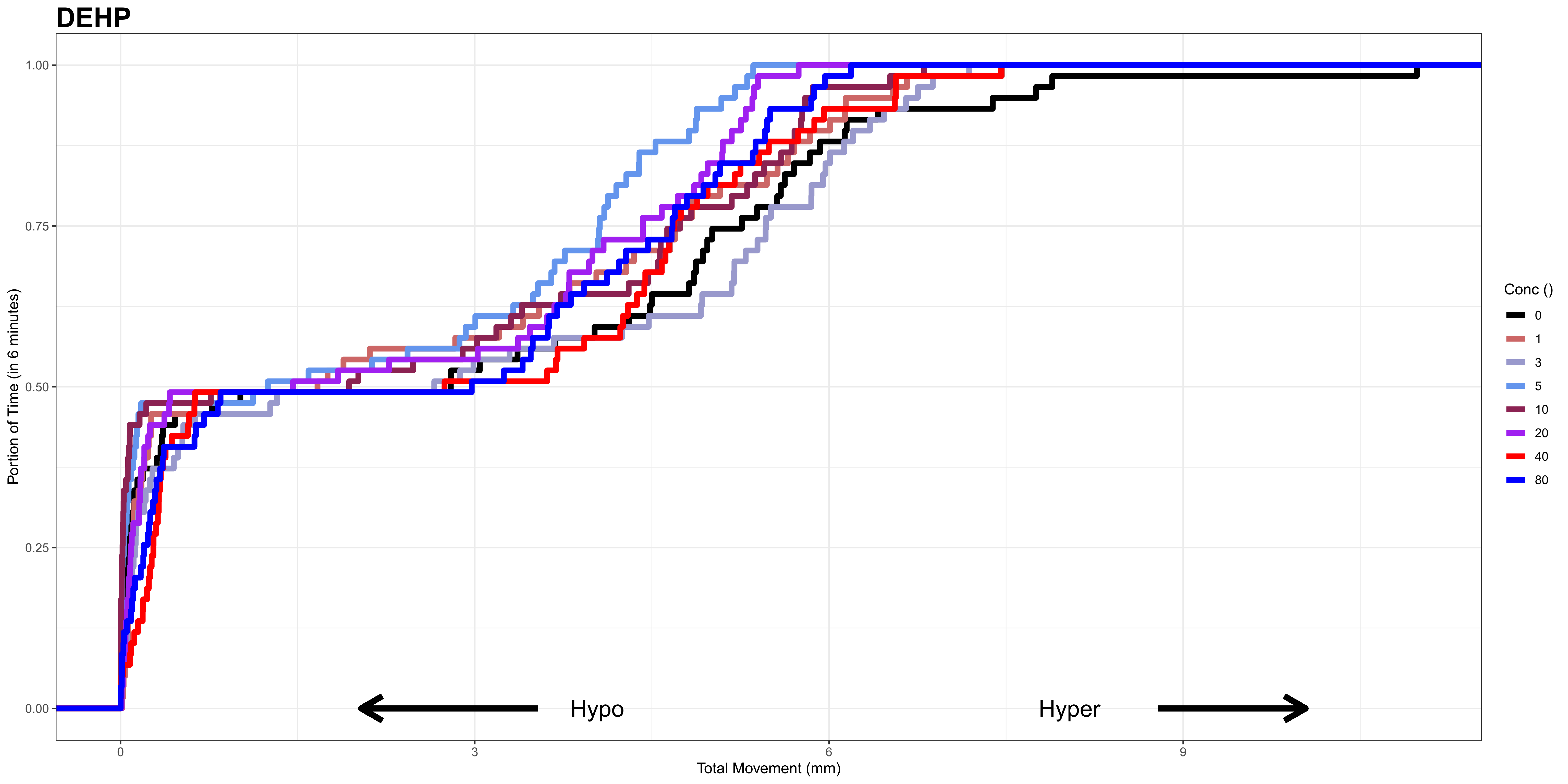
DEHP
Portion of Time (in 6 minutes)
1.00
0.75
0.50
0.25
0.00
0
3
6
9
Total Movement (mm)
Hypo
Hyper
Conc ()
0
1
3
5
10
20
40
80

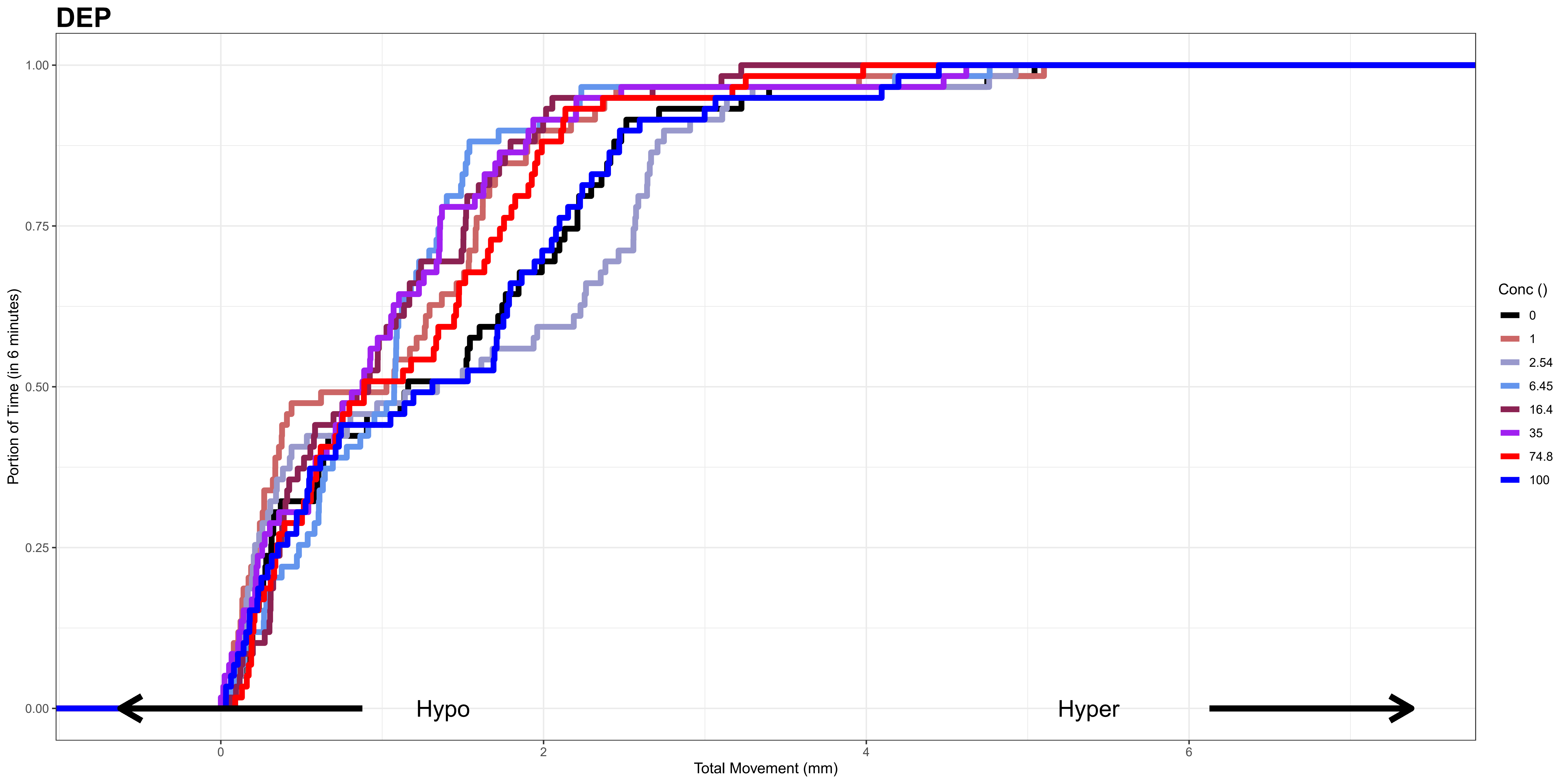
DEP
Portion of Time (in 6 minutes)
1.00
0.75
0.50
0.25
0.00
0
2
4
6
Total Movement (mm)
Hypo
Hyper
Conc ()
0
1
2.54
6.45
16.4
35
74.8
100

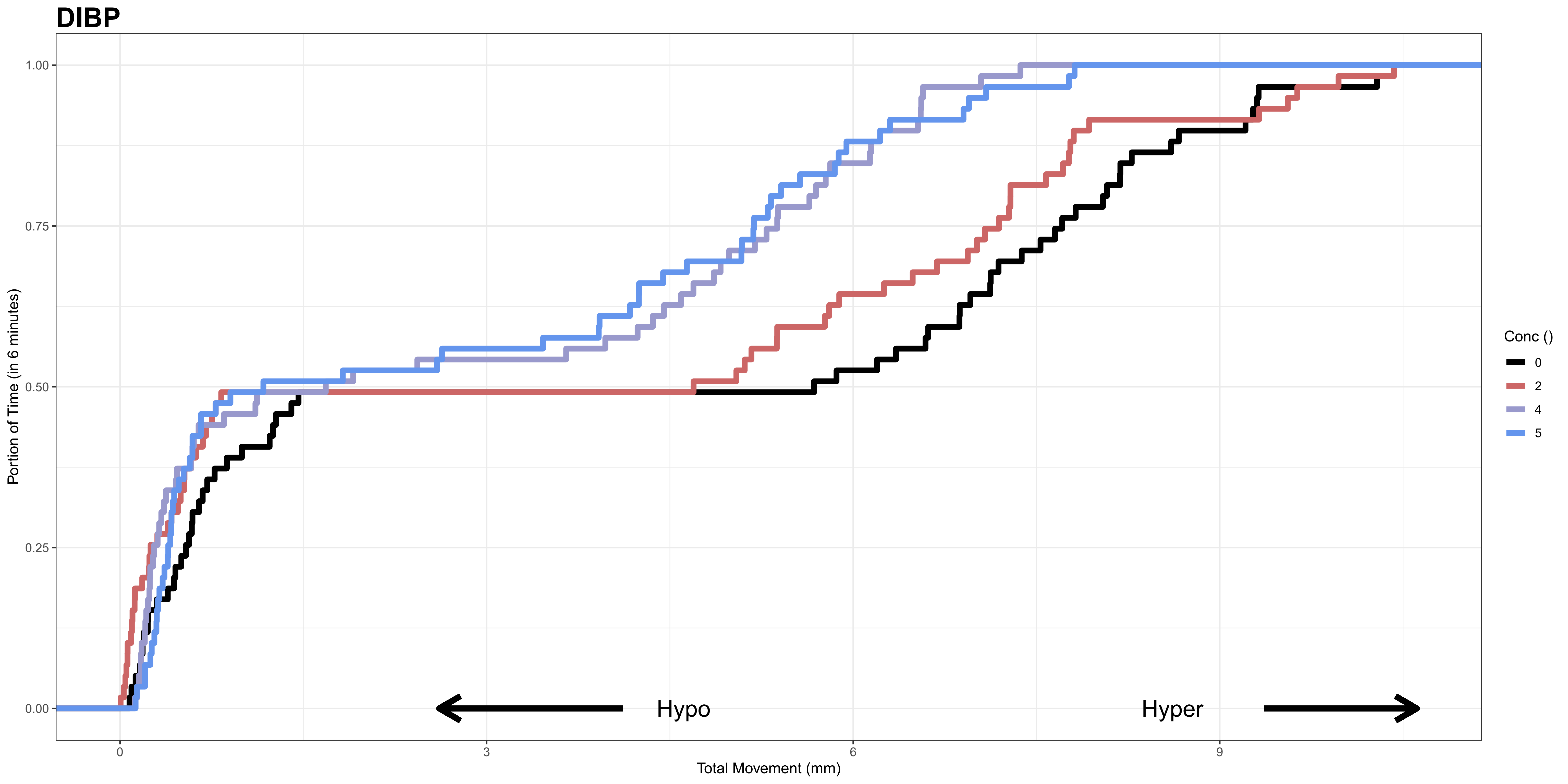

DIBP
Portion of Time (in 6 minutes)
1.00
0.75
0.50
0.25
0.00
0
3
6
9
Total Movement (mm)
Hypo
Hyper
Conc ()
0
2
4
5

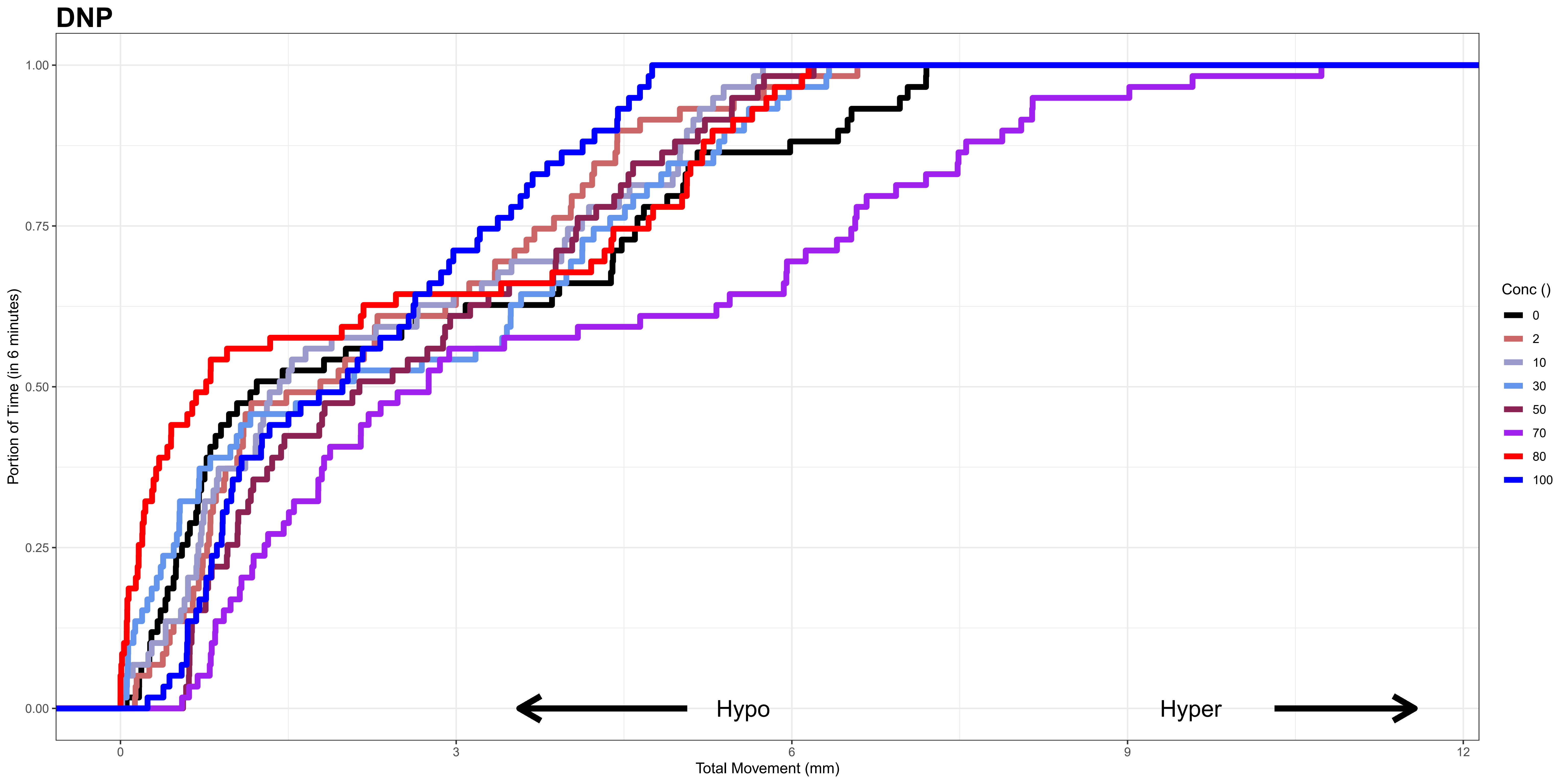

DNP
Portion of Time (in 6 minutes)
1.00
0.75
0.50
0.25
0.00
0
3
6
9
12
Total Movement (mm)
Hypo
Hyper
Conc ()
0
2
10
30
50
70
80
100

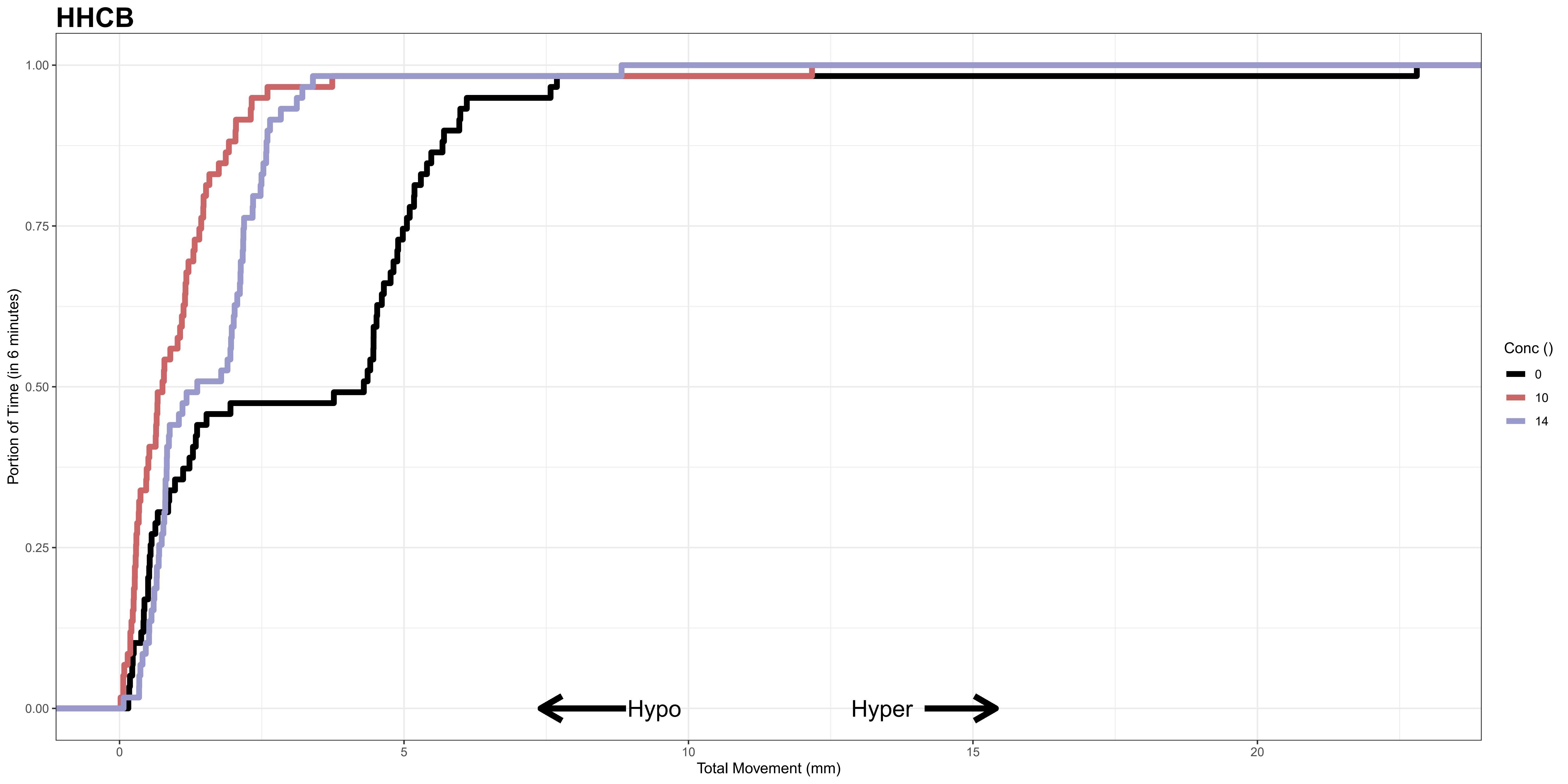
HHCB
Portion of Time (in 6 minutes)
1.00
0.75
0.50
0.25
0.00
0
5
10
15
20
Total Movement (mm)
Hypo
Hyper
Conc ()
0
10
14

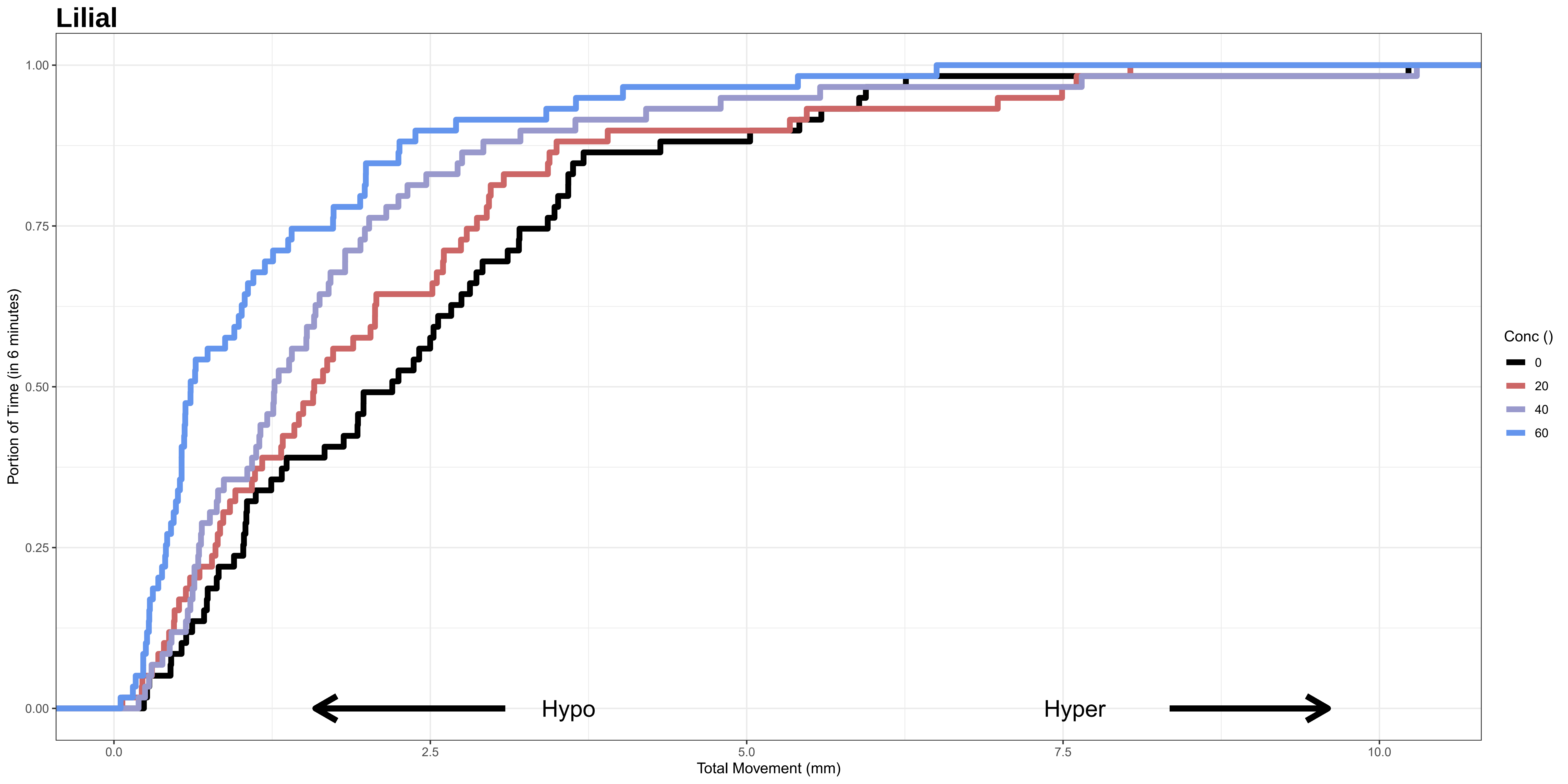

Lilial
Portion of Time (in 6 minutes)
1.00
0.75
0.50
0.25
0.00
0.0
2.5
5.0
7.5
10.0
Total Movement (mm)
Hypo
Hyper
Conc ()
0
20
40
60

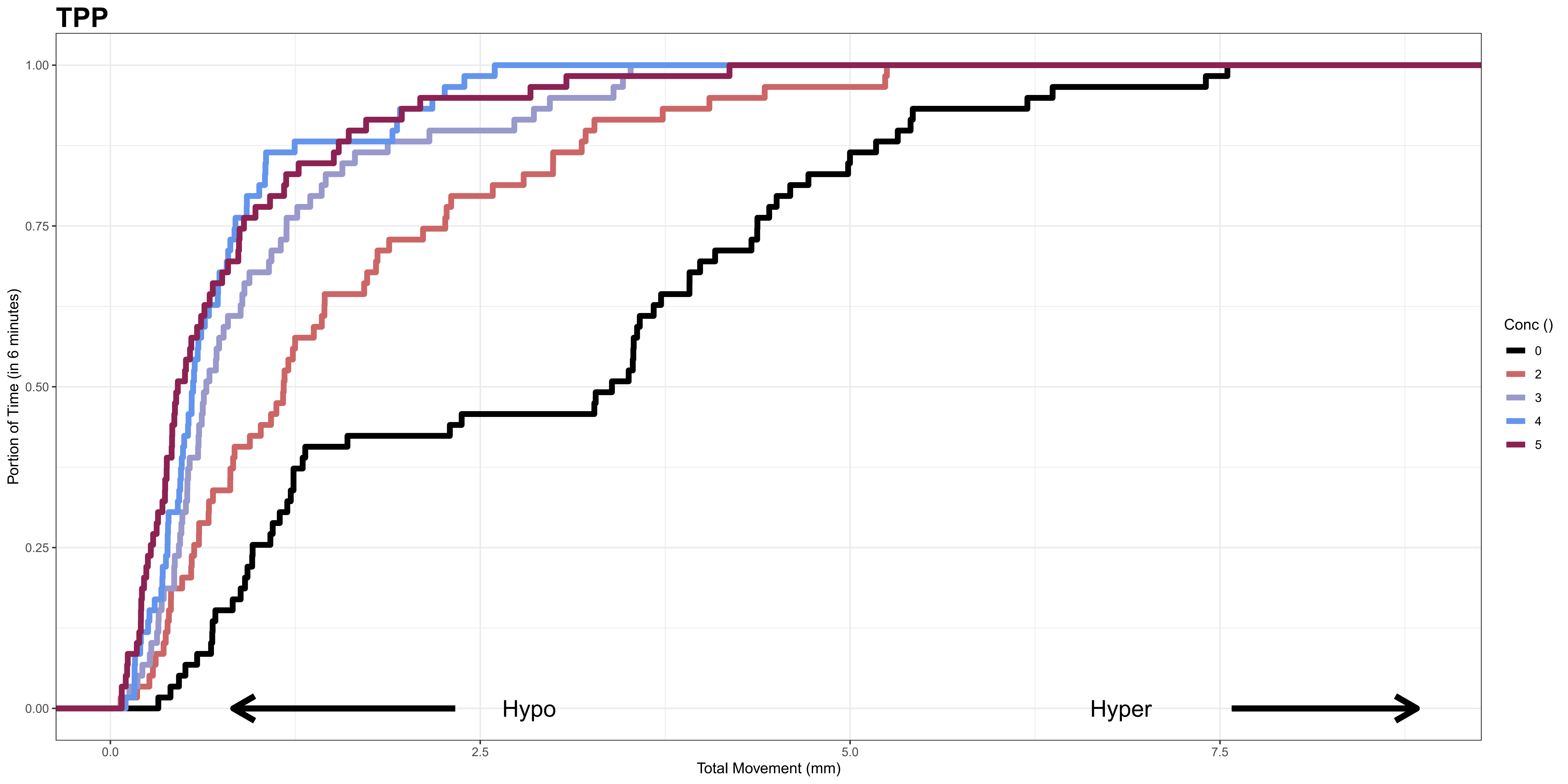

TPP
Portion of Time (in 6 minutes)
1.00
0.75
0.50
0.25
0.00
0.0
2.5
5.0
7.5
Total Movement (mm)
Hypo
Hyper
Conc ()
0
2
3
4
5

G14 mix

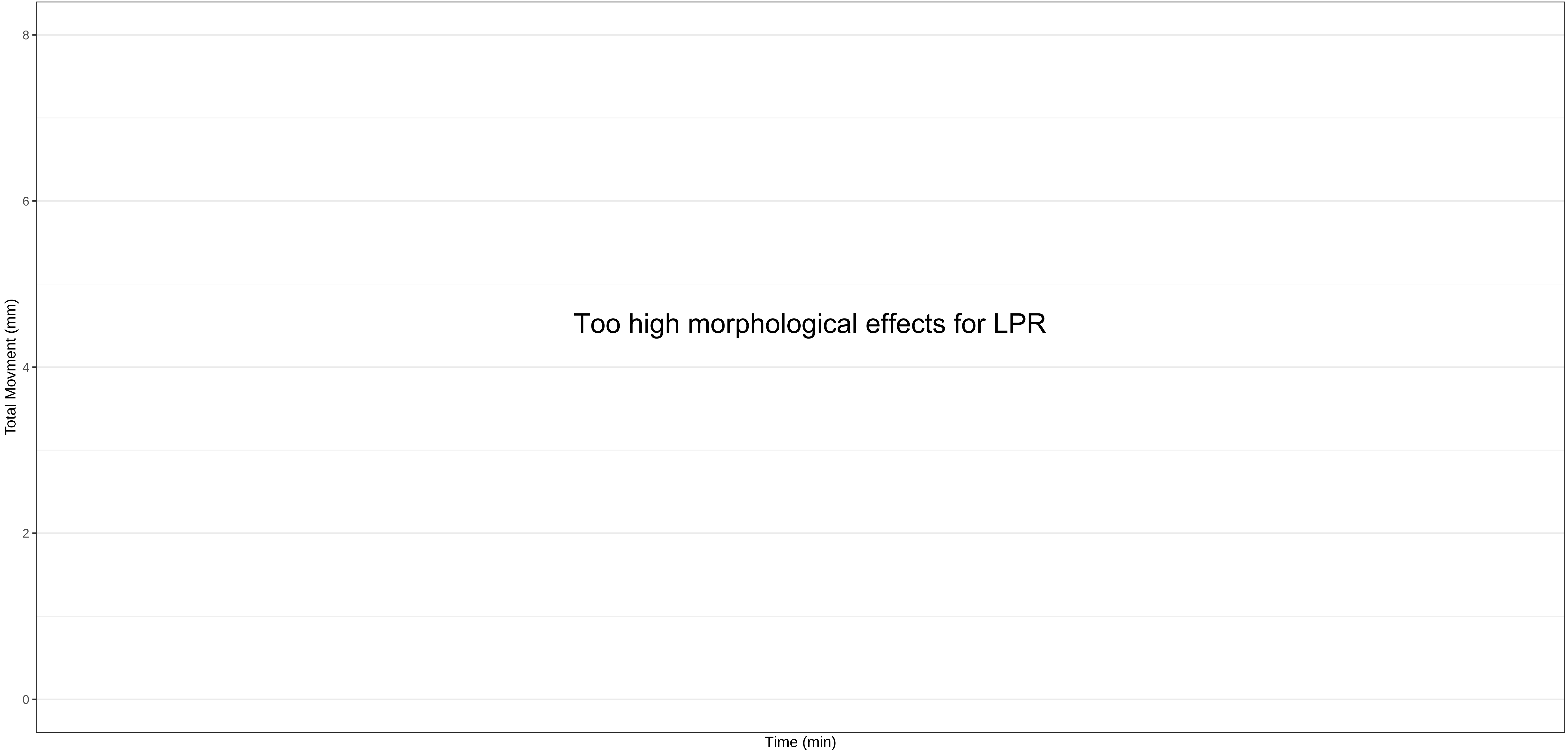

Too high morphological effects for LPR
Total Movment (mm)
Time (min)
0
2
4
6
8

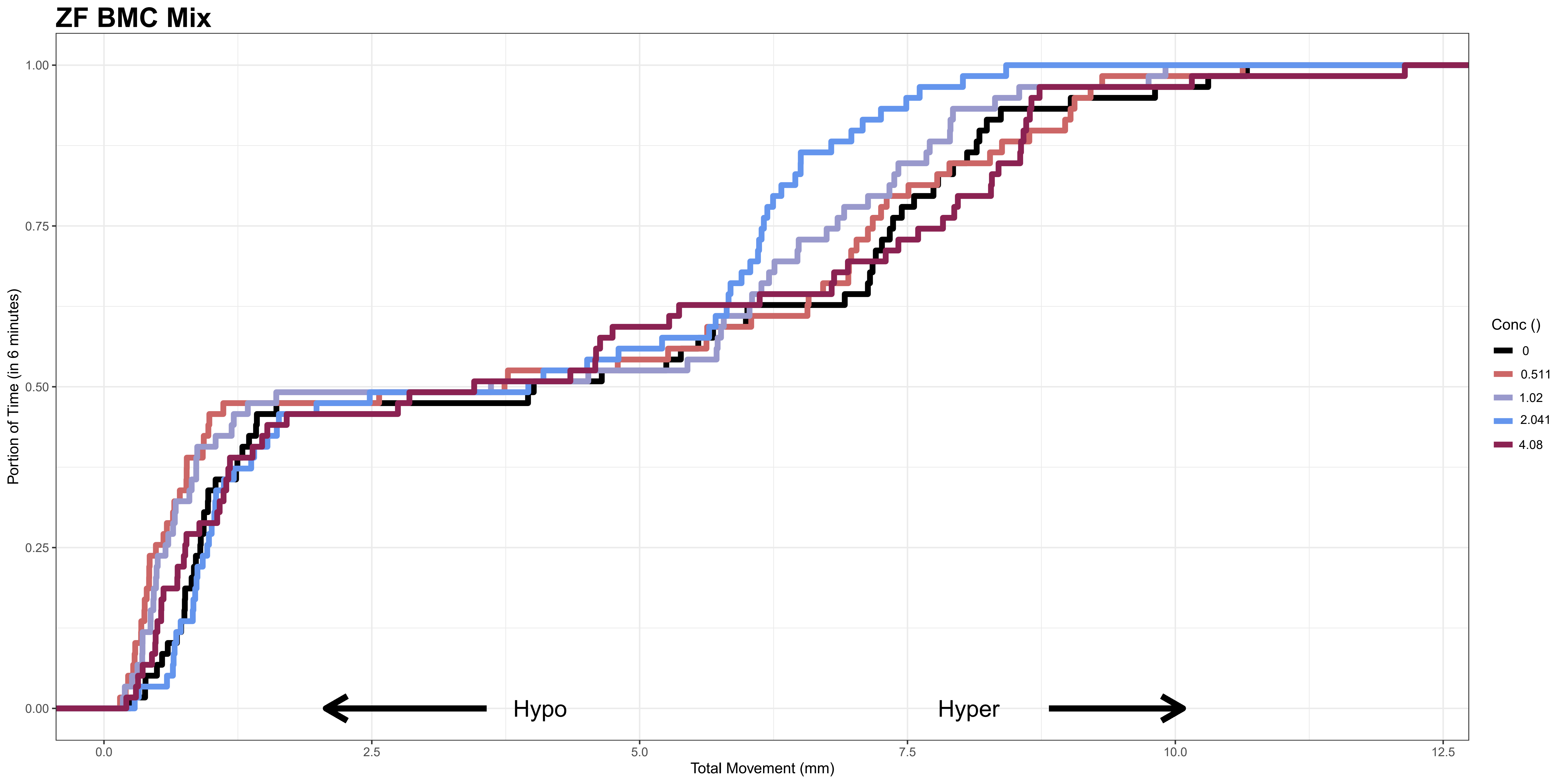
ZF BMC Mix
Portion of Time (in 6 minutes)
1.00
0.75
0.50
0.25
0.00
0.0
2.5
5.0
7.5
10.0
12.5
Total Movement (mm)
Hypo
Hyper
Conc ()
0
0.511
1.02
2.041
4.08

ZF Equi-Mix

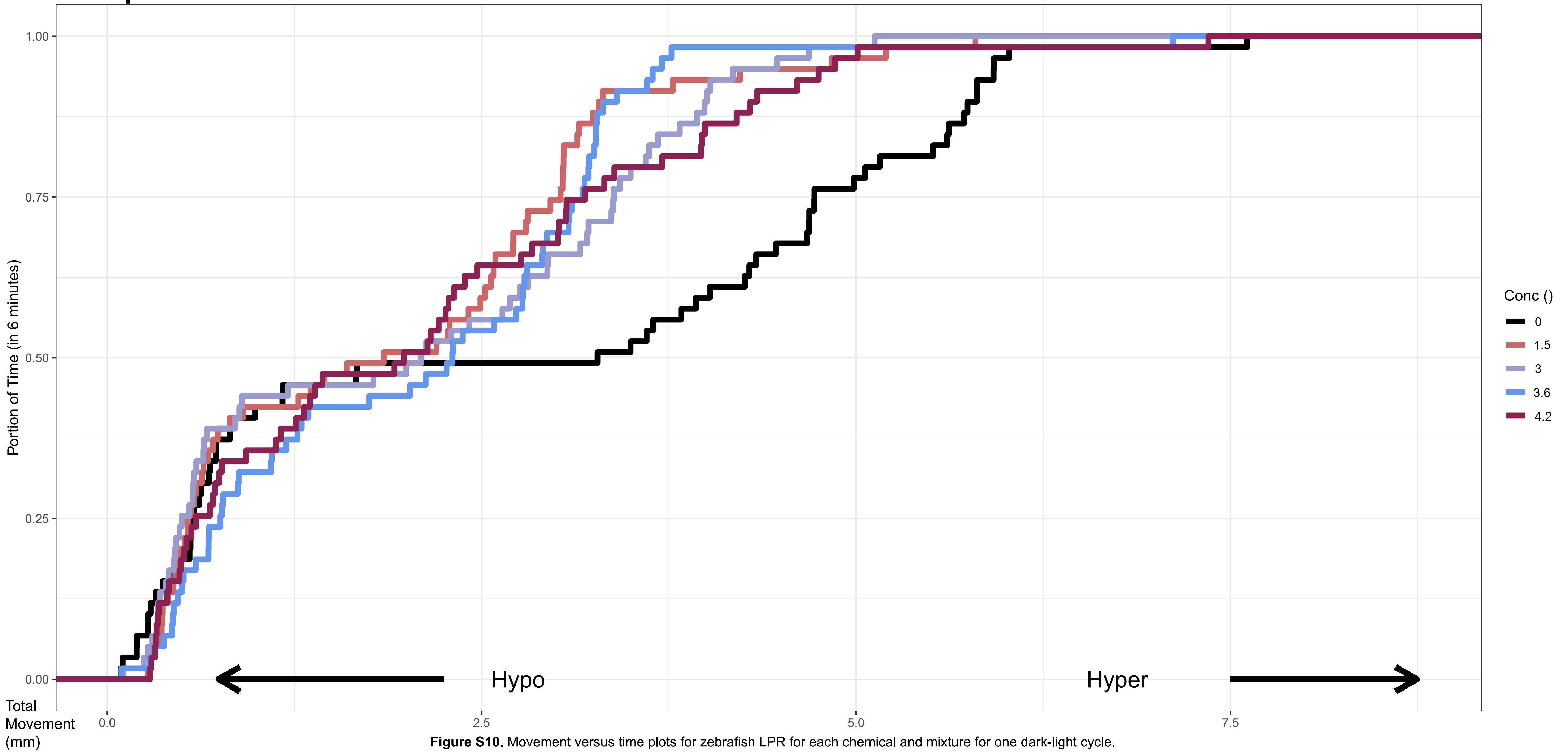

Figure S10. Movement versus time plots for zebrafish LPR for each chemical and mixture for one dark-light cycle.

Table S7. Larval photomotor response (LPR) compiled data for all chemicals and mixtures tested in zebrafish.

Chemical	Concentration (µM)	Phase	# viable animals	# total animals	% viable animals	More than 70% viable	AUC	Pval	RelativeRatio	Significance p<0.01	Activity
AHTN	0	Dark	11	12	0.92	TRUE	79.621	1	0	NA	NA
AHTN	10	Dark	11	12	0.92	TRUE	83.148	0.135	0.044	NA	NA
AHTN	30	Dark	12	12	1	TRUE	77.308	0.808	-0.029	NA	NA
AHTN	50	Dark	10	12	0.83	TRUE	72.328	0.239	-0.092	NA	NA
AHTN	60	Dark	7	12	0.58	FALSE	74.801	0.239	-0.061	NA	NA
AHTN	70	Dark	7	12	0.58	FALSE	NA	NA	NA	NA	NA
AHTN	80	Dark	4	12	0.33	FALSE	NA	NA	NA	NA	NA
AHTN	100	Dark	1	12	0.08	FALSE	NA	NA	NA	NA	NA
AHTN	0	Light	11	12	0.92	TRUE	23.087	1	0	NA	NA
AHTN	10	Light	11	12	0.92	TRUE	28.799	0.564	0.247	NA	NA
AHTN	30	Light	12	12	1	TRUE	26.598	0.945	0.152	NA	NA
AHTN	50	Light	10	12	0.83	TRUE	26.629	0.064	0.153	NA	NA
AHTN	60	Light	7	12	0.58	FALSE	31.841	0.064	0.379	NA	NA
AHTN	70	Light	7	12	0.58	FALSE	NA	NA	NA	NA	NA
AHTN	80	Light	4	12	0.33	FALSE	NA	NA	NA	NA	NA
AHTN	100	Light	1	12	0.08	FALSE	NA	NA	NA	NA	NA
BBP	0	Dark	35	36	0.97	TRUE	52.512	1	0	NA	NA
BBP	2.25	Dark	36	36	1	TRUE	72.301	0.015	0.377	NA	NA
BBP	3	Dark	32	36	0.89	TRUE	68.401	0.015	0.303	NA	NA
BBP	3.5	Dark	31	36	0.86	TRUE	52.056	0.081	-0.009	NA	NA
BBP	4	Dark	14	24	0.58	FALSE	82.728	0.004	0.575	NA	NA
BBP	4.5	Dark	10	24	0.42	FALSE	NA	NA	NA	NA	NA
BBP	5	Dark	5	24	0.21	FALSE	NA	NA	NA	NA	NA
BBP	8	Dark	0	24	0	FALSE	NA	NA	NA	NA	NA
BBP	0	Light	35	36	0.97	TRUE	57.444	1	0	NA	NA
BBP	2.25	Light	36	36	1	TRUE	34.46	0.001	-0.4	YES	HYPO
BBP	3	Light	32	36	0.89	TRUE	57.926	0.012	0.008	NA	NA
BBP	3.5	Light	31	36	0.86	TRUE	40.229	0.02	-0.3	NA	NA
BBP	4	Light	14	24	0.58	FALSE	75.329	0.003	0.311	NA	NA
BBP	4.5	Light	10	24	0.42	FALSE	NA	NA	NA	NA	NA
BBP	5	Light	5	24	0.21	FALSE	NA	NA	NA	NA	NA
BBP	8	Light	0	24	0	FALSE	NA	NA	NA	NA	NA
BHT	0	Dark	21	24	0.88	TRUE	83.127	1	0	NA	NA
BHT	1	Dark	20	24	0.83	TRUE	78.452	0.076	-0.056	NA	NA
BHT	2.54	Dark	23	24	0.96	TRUE	57.497	0.12	-0.308	NA	NA
BHT	6.45	Dark	23	24	0.96	TRUE	75.727	0.665	-0.089	NA	NA
BHT	16.4	Dark	23	24	0.96	TRUE	88.632	0.182	0.066	NA	NA
BHT	35	Dark	23	24	0.96	TRUE	74.97	0.182	-0.098	NA	NA
BHT	74.8	Dark	23	24	0.96	TRUE	74.595	0.378	-0.103	NA	NA
BHT	100	Dark	23	24	0.96	TRUE	70.236	0.016	-0.155	NA	NA
BHT	0	Light	21	24	0.88	TRUE	18.427	1	0	NA	NA
BHT	1	Light	20	24	0.83	TRUE	8.053	0.355	-0.563	NA	NA
BHT	2.54	Light	23	24	0.96	TRUE	28.55	0.916	0.549	NA	NA
BHT	6.45	Light	23	24	0.96	TRUE	24.025	0.355	0.304	NA	NA
BHT	16.4	Light	23	24	0.96	TRUE	7.004	0.068	-0.62	NA	NA
BHT	35	Light	23	24	0.96	TRUE	24.736	0.167	0.342	NA	NA
BHT	74.8	Light	23	24	0.96	TRUE	10.322	0	-0.44	YES	HYPO
BHT	100	Light	23	24	0.96	TRUE	27.472	0	0.491	YES	HYPER
BP	0	Dark	31	36	0.86	TRUE	63.757	1	0	NA	NA
BP	20	Dark	33	36	0.92	TRUE	52.129	0.023	-0.182	NA	NA

BP	40	Dark	25	36	0.69	FALSE	50.563	0	-0.207	NA	NA
BP	50	Dark	30	36	0.83	TRUE	40.462	0	-0.365	YES	HYPO
BP	60	Dark	22	36	0.61	FALSE	41.033	0	-0.356	NA	NA
BP	70	Dark	27	36	0.75	TRUE	6.368	0	-0.9	YES	HYPO
BP	80	Dark	25	36	0.69	FALSE	17.655	0	-0.723	NA	NA
BP	100	Dark	16	36	0.44	FALSE	NA	NA	NA	NA	NA
BP	0	Light	31	36	0.86	TRUE	18.586	1	0	NA	NA
BP	20	Light	33	36	0.92	TRUE	17.547	0.21	-0.056	NA	NA
BP	40	Light	25	36	0.69	FALSE	3.107	0.21	-0.833	NA	NA
BP	50	Light	30	36	0.83	TRUE	12.358	0.379	-0.335	NA	NA
BP	60	Light	22	36	0.61	FALSE	36.827	0.02	0.981	NA	NA
BP	70	Light	27	36	0.75	TRUE	21.409	0.379	0.152	NA	NA
BP	80	Light	25	36	0.69	FALSE	24.142	0.49	0.299	NA	NA
BP	100	Light	16	36	0.44	FALSE	NA	NA	NA	NA	NA
BS	0	Dark	35	36	0.97	TRUE	67.094	1	0	NA	NA
BS	5	Dark	33	36	0.92	TRUE	59.228	0.001	-0.117	YES	HYPO
BS	10	Dark	33	36	0.92	TRUE	63.141	0.762	-0.059	NA	NA
BS	14	Dark	21	36	0.58	FALSE	NA	NA	NA	NA	NA
BS	18	Dark	9	36	0.25	FALSE	NA	NA	NA	NA	NA
BS	22	Dark	1	36	0.03	FALSE	NA	NA	NA	NA	NA
BS	30	Dark	0	36	0	FALSE	NA	NA	NA	NA	NA
BS	50	Dark	0	36	0	FALSE	NA	NA	NA	NA	NA
BS	0	Light	35	36	0.97	TRUE	29.607	1	0	NA	NA
BS	5	Light	33	36	0.92	TRUE	15.708	0.286	-0.469	NA	NA
BS	10	Light	33	36	0.92	TRUE	38.52	0.013	0.301	NA	NA
BS	14	Light	21	36	0.58	FALSE	NA	NA	NA	NA	NA
BS	18	Light	9	36	0.25	FALSE	NA	NA	NA	NA	NA
BS	22	Light	1	36	0.03	FALSE	NA	NA	NA	NA	NA
BS	30	Light	0	36	0	FALSE	NA	NA	NA	NA	NA
BS	50	Light	0	36	0	FALSE	NA	NA	NA	NA	NA
DBP	0	Dark	31	36	0.86	TRUE	114.433	1	0	NA	NA
DBP	1	Dark	29	36	0.81	TRUE	79.579	0.001	-0.305	YES	HYPO
DBP	2	Dark	33	36	0.92	TRUE	55.486	0.036	-0.515	NA	NA
DBP	3	Dark	22	36	0.61	FALSE	55.447	0	-0.515	NA	NA
DBP	4	Dark	21	36	0.58	FALSE	NA	NA	NA	NA	NA
DBP	5	Dark	9	36	0.25	FALSE	NA	NA	NA	NA	NA
DBP	10	Dark	0	36	0	FALSE	NA	NA	NA	NA	NA
DBP	20	Dark	0	36	0	FALSE	NA	NA	NA	NA	NA
DBP	0	Light	31	36	0.86	TRUE	13.132	1	0	NA	NA
DBP	1	Light	29	36	0.81	TRUE	19.811	0.105	0.509	NA	NA
DBP	2	Light	33	36	0.92	TRUE	13.144	0.286	0.001	NA	NA
DBP	3	Light	22	36	0.61	FALSE	13.806	0	0.051	NA	NA
DBP	4	Light	21	36	0.58	FALSE	NA	NA	NA	NA	NA
DBP	5	Light	9	36	0.25	FALSE	NA	NA	NA	NA	NA
DBP	10	Light	0	36	0	FALSE	NA	NA	NA	NA	NA
DBP	20	Light	0	36	0	FALSE	NA	NA	NA	NA	NA
DEET	0	Dark	34	36	0.94	TRUE	73.856	1	0	NA	NA
DEET	1	Dark	33	36	0.92	TRUE	63.092	0.002	-0.146	YES	HYPO
DEET	2.54	Dark	33	36	0.92	TRUE	66.598	0.001	-0.098	YES	NA
DEET	6.45	Dark	30	36	0.83	TRUE	95.121	0.635	0.288	NA	NA
DEET	16.4	Dark	31	36	0.86	TRUE	67.903	0.512	-0.081	NA	NA

DEET	35	Dark	31	36	0.86	TRUE	97.11	0.95	0.315	NA	NA
DEET	74.8	Dark	30	35	0.86	TRUE	56.972	0.001	-0.229	YES	HYPO
DEET	100	Dark	33	36	0.92	TRUE	68.828	0	-0.068	YES	NA
DEET	0	Light	34	36	0.94	TRUE	20.476	1	0	NA	NA
DEET	1	Light	33	36	0.92	TRUE	53.37	0.032	1.607	NA	NA
DEET	2.54	Light	33	36	0.92	TRUE	36.173	0.151	0.767	NA	NA
DEET	6.45	Light	30	36	0.83	TRUE	9.817	0.003	-0.521	YES	HYPO
DEET	16.4	Light	31	36	0.86	TRUE	9.283	0.002	-0.547	YES	HYPO
DEET	35	Light	31	36	0.86	TRUE	44.441	0.005	1.17	YES	HYPER
DEET	74.8	Light	30	35	0.86	TRUE	46.118	0.49	1.252	NA	NA
DEET	100	Light	33	36	0.92	TRUE	39.142	0.49	0.912	NA	NA
DEHP	0	Dark	33	36	0.92	TRUE	64.393	1	0	NA	NA
DEHP	1	Dark	35	36	0.97	TRUE	53.69	0.515	-0.166	NA	NA
DEHP	3	Dark	36	36	1	TRUE	79.732	0.515	0.238	NA	NA
DEHP	5	Dark	35	36	0.97	TRUE	80.529	0.015	0.251	NA	NA
DEHP	10	Dark	35	36	0.97	TRUE	80.761	0.762	0.254	NA	NA
DEHP	20	Dark	32	36	0.89	TRUE	63.7	0.081	-0.011	NA	NA
DEHP	40	Dark	35	36	0.97	TRUE	66.57	0.116	0.034	NA	NA
DEHP	80	Dark	35	36	0.97	TRUE	75.097	0.015	0.166	NA	NA
DEHP	0	Light	33	36	0.92	TRUE	42.629	1	0	NA	NA
DEHP	1	Light	35	36	0.97	TRUE	31.816	0.013	-0.254	NA	NA
DEHP	3	Light	36	36	1	TRUE	29.494	0.614	-0.308	NA	NA
DEHP	5	Light	35	36	0.97	TRUE	55.708	0.151	0.307	NA	NA
DEHP	10	Light	35	36	0.97	TRUE	47.498	0	0.114	YES	HYPER
DEHP	20	Light	32	36	0.89	TRUE	5.095	0.74	-0.88	NA	NA
DEHP	40	Light	35	36	0.97	TRUE	16.551	0.072	-0.612	NA	NA
DEHP	80	Light	35	36	0.97	TRUE	5.699	0.005	-0.866	YES	HYPO
DEP	0	Dark	30	36	0.83	TRUE	79.84	1	0	NA	NA
DEP	1	Dark	35	36	0.97	TRUE	68.737	0.4	-0.139	NA	NA
DEP	2.54	Dark	35	36	0.97	TRUE	64.511	0.001	-0.192	YES	HYPO
DEP	6.45	Dark	35	36	0.97	TRUE	52.704	0.305	-0.34	NA	NA
DEP	16.4	Dark	36	36	1	TRUE	77.055	0.009	-0.035	YES	NA
DEP	35	Dark	35	36	0.97	TRUE	68.978	0.081	-0.136	NA	NA
DEP	74.8	Dark	35	36	0.97	TRUE	68.525	0.055	-0.142	NA	NA
DEP	100	Dark	36	36	1	TRUE	70.126	0.036	-0.122	NA	NA
DEP	0	Light	30	36	0.83	TRUE	29.857	1	0	NA	NA
DEP	1	Light	35	36	0.97	TRUE	5.098	0.105	-0.829	NA	NA
DEP	2.54	Light	35	36	0.97	TRUE	11.005	0.379	-0.631	NA	NA
DEP	6.45	Light	35	36	0.97	TRUE	18.295	0.379	-0.387	NA	NA
DEP	16.4	Light	36	36	1	TRUE	11.781	0.286	-0.605	NA	NA
DEP	35	Light	35	36	0.97	TRUE	6.014	0.856	-0.799	NA	NA
DEP	74.8	Light	35	36	0.97	TRUE	41.49	0.941	0.39	NA	NA
DEP	100	Light	36	36	1	TRUE	5.598	0.286	-0.812	NA	NA
DIBP	0	Dark	36	36	1	TRUE	81.115	1	0	NA	NA
DIBP	2	Dark	33	36	0.92	TRUE	91.552	0.081	0.129	NA	NA
DIBP	4	Dark	29	36	0.81	TRUE	61.76	0.762	-0.239	NA	NA
DIBP	5	Dark	28	36	0.78	TRUE	101.226	0.023	0.248	NA	NA
DIBP	6	Dark	20	36	0.56	FALSE	NA	NA	NA	NA	NA
DIBP	8	Dark	5	36	0.14	FALSE	NA	NA	NA	NA	NA
DIBP	10	Dark	0	36	0	FALSE	NA	NA	NA	NA	NA
DIBP	15	Dark	0	36	0	FALSE	NA	NA	NA	NA	NA

DIBP	0	Light	36	36	1	TRUE	11.958	1	0	NA	NA
DIBP	2	Light	33	36	0.92	TRUE	4.563	0.008	-0.618	YES	HYPO
DIBP	4	Light	29	36	0.81	TRUE	30.116	0.286	1.518	NA	NA
DIBP	5	Light	28	36	0.78	TRUE	19.083	0.048	0.596	NA	NA
DIBP	6	Light	20	36	0.56	FALSE	NA	NA	NA	NA	NA
DIBP	8	Light	5	36	0.14	FALSE	NA	NA	NA	NA	NA
DIBP	10	Light	0	36	0	FALSE	NA	NA	NA	NA	NA
DIBP	15	Light	0	36	0	FALSE	NA	NA	NA	NA	NA
DNP	0	Dark	34	36	0.94	TRUE	84.512	1	0	NA	NA
DNP	2	Dark	35	36	0.97	TRUE	99.096	0.036	0.173	NA	NA
DNP	10	Dark	35	36	0.97	TRUE	104.124	0.009	0.232	YES	HYPER
DNP	30	Dark	35	36	0.97	TRUE	99.621	0.635	0.179	NA	NA
DNP	50	Dark	33	35	0.94	TRUE	109.334	0.036	0.294	NA	NA
DNP	70	Dark	34	36	0.94	TRUE	106.927	0.055	0.265	NA	NA
DNP	80	Dark	35	36	0.97	TRUE	106.448	0.116	0.26	NA	NA
DNP	100	Dark	31	36	0.86	TRUE	101.953	0	0.206	YES	HYPER
DNP	0	Light	34	36	0.94	TRUE	50.083	1	0	NA	NA
DNP	2	Light	35	36	0.97	TRUE	32.508	0.21	-0.351	NA	NA
DNP	10	Light	35	36	0.97	TRUE	15.064	0.105	-0.699	NA	NA
DNP	30	Light	35	36	0.97	TRUE	57.18	0	0.142	YES	HYPER
DNP	50	Light	33	35	0.94	TRUE	40.603	0.001	-0.189	YES	HYPO
DNP	70	Light	34	36	0.94	TRUE	8.08	0	-0.839	YES	HYPO
DNP	80	Light	35	36	0.97	TRUE	45.659	0	-0.088	YES	NA
DNP	100	Light	31	36	0.86	TRUE	13.421	0.001	-0.732	YES	HYPO
G14 Mix	0	Dark	32	36	0.89	TRUE	18.679	1	0	NA	NA
G14 Mix	28	Dark	23	36	0.64	FALSE	13.906	0.002	-0.338	NA	NA
G14 Mix	56	Dark	14	36	0.39	FALSE	NA	NA	NA	NA	NA
G14 Mix	63	Dark	2	36	0.06	FALSE	NA	NA	NA	NA	NA
G14 Mix	70	Dark	4	36	0.11	FALSE	NA	NA	NA	NA	NA
G14 Mix	77	Dark	0	36	0	FALSE	NA	NA	NA	NA	NA
G14 Mix	84	Dark	0	36	0	FALSE	NA	NA	NA	NA	NA
G14 Mix	112	Dark	0	36	0	FALSE	NA	NA	NA	NA	NA
G14 Mix	0	Light	32	36	0.89	TRUE	14.487	1	0	NA	NA
G14 Mix	28	Light	23	36	0.64	FALSE	13.493	0.001	-0.785	NA	NA
G14 Mix	56	Light	14	36	0.39	FALSE	NA	NA	NA	NA	NA
G14 Mix	63	Light	2	36	0.06	FALSE	NA	NA	NA	NA	NA
G14 Mix	70	Light	4	36	0.11	FALSE	NA	NA	NA	NA	NA
G14 Mix	77	Light	0	36	0	FALSE	NA	NA	NA	NA	NA
G14 Mix	84	Light	0	36	0	FALSE	NA	NA	NA	NA	NA
G14 Mix	112	Light	0	36	0	FALSE	NA	NA	NA	NA	NA
HHCB	0	Dark	35	36	0.97	TRUE	83.837	1	0	NA	NA
HHCB	10	Dark	30	36	0.83	TRUE	123.374	0	0.472	YES	HYPER
HHCB	14	Dark	28	36	0.78	TRUE	77.473	0	-0.076	YES	NA
HHCB	16	Dark	25	36	0.69	FALSE	111.206	0	0.326	NA	NA
HHCB	20	Dark	10	36	0.28	FALSE	NA	NA	NA	NA	NA
HHCB	24	Dark	0	36	0	FALSE	NA	NA	NA	NA	NA
HHCB	28	Dark	0	36	0	FALSE	NA	NA	NA	NA	NA
HHCB	32	Dark	0	36	0	FALSE	NA	NA	NA	NA	NA
HHCB	0	Light	35	36	0.97	TRUE	36.368	1	0	NA	NA
HHCB	10	Light	30	36	0.83	TRUE	13.099	0.008	-0.64	YES	HYPO
HHCB	14	Light	28	36	0.78	TRUE	18.47	0.379	-0.492	NA	NA

HHCB	16	Light	25	36	0.69	FALSE	13.351	0	-0.633	NA	NA
HHCB	20	Light	10	36	0.28	FALSE	NA	NA	NA	NA	NA
HHCB	24	Light	0	36	0	FALSE	NA	NA	NA	NA	NA
HHCB	28	Light	0	36	0	FALSE	NA	NA	NA	NA	NA
HHCB	32	Light	0	36	0	FALSE	NA	NA	NA	NA	NA
Lilial	0	Dark	34	36	0.94	TRUE	62.416	1	0	NA	NA
Lilial	20	Dark	35	36	0.97	TRUE	36.487	0.164	-0.415	NA	NA
Lilial	40	Dark	32	36	0.89	TRUE	33.854	0	-0.458	YES	HYPO
Lilial	60	Dark	31	36	0.86	TRUE	28.3	0	-0.547	YES	HYPO
Lilial	70	Dark	21	36	0.58	FALSE	NA	NA	NA	NA	NA
Lilial	80	Dark	17	36	0.47	FALSE	NA	NA	NA	NA	NA
Lilial	90	Dark	13	36	0.36	FALSE	NA	NA	NA	NA	NA
Lilial	100	Dark	10	36	0.28	FALSE	NA	NA	NA	NA	NA
Lilial	0	Light	34	36	0.94	TRUE	39.413	1	0	NA	NA
Lilial	20	Light	35	36	0.97	TRUE	1.694	0.105	-0.957	NA	NA
Lilial	40	Light	32	36	0.89	TRUE	0.986	0.286	-0.975	NA	NA
Lilial	60	Light	31	36	0.86	TRUE	27.062	0.005	-0.313	YES	HYPO
Lilial	70	Light	21	36	0.58	FALSE	NA	NA	NA	NA	NA
Lilial	80	Light	17	36	0.47	FALSE	NA	NA	NA	NA	NA
Lilial	90	Light	13	36	0.36	FALSE	NA	NA	NA	NA	NA
Lilial	100	Light	10	36	0.28	FALSE	NA	NA	NA	NA	NA
TPP	0	Dark	36	36	1	TRUE	82.582	1	0	NA	NA
TPP	2	Dark	35	36	0.97	TRUE	35.264	0	-0.573	YES	HYPO
TPP	3	Dark	36	36	1	TRUE	43.229	0	-0.477	YES	HYPO
TPP	4	Dark	33	36	0.92	TRUE	10.975	0	-0.867	YES	HYPO
TPP	5	Dark	29	36	0.81	TRUE	17.859	0	-0.784	YES	HYPO
TPP	6	Dark	19	36	0.53	FALSE	NA	NA	NA	NA	NA
TPP	8	Dark	12	36	0.33	FALSE	NA	NA	NA	NA	NA
TPP	10	Dark	5	36	0.14	FALSE	NA	NA	NA	NA	NA
TPP	0	Light	36	36	1	TRUE	8.733	1	0	NA	NA
TPP	2	Light	35	36	0.97	TRUE	9.54	0.032	0.092	NA	NA
TPP	3	Light	36	36	1	TRUE	24.324	0.105	1.785	NA	NA
TPP	4	Light	33	36	0.92	TRUE	10.169	0.005	0.164	YES	HYPER
TPP	5	Light	29	36	0.81	TRUE	1.125	0.002	-0.871	YES	HYPO
TPP	6	Light	19	36	0.53	FALSE	NA	NA	NA	NA	NA
TPP	8	Light	12	36	0.33	FALSE	NA	NA	NA	NA	NA
TPP	10	Light	5	36	0.14	FALSE	NA	NA	NA	NA	NA
ZF BMC Mix	0	Dark	34	36	0.94	TRUE	76.369	1	0	NA	NA
ZF BMC Mix	0.511	Dark	29	36	0.81	TRUE	84.237	0.515	0.103	NA	NA
ZF BMC Mix	1.02	Dark	33	36	0.92	TRUE	75.275	0.515	-0.014	NA	NA
ZF BMC Mix	2.041	Dark	30	36	0.83	TRUE	76.86	0.054	0.006	NA	NA
ZF BMC Mix	4.08	Dark	27	36	0.75	TRUE	96.208	0.306	0.26	NA	NA
ZF BMC Mix	6.13	Dark	17	36	0.47	FALSE	NA	NA	NA	NA	NA
ZF BMC Mix	8.16	Dark	8	36	0.22	FALSE	NA	NA	NA	NA	NA
ZF BMC Mix	10.2	Dark	1	36	0.03	FALSE	NA	NA	NA	NA	NA
ZF BMC Mix	0	Light	34	36	0.94	TRUE	29.774	1	0	NA	NA
ZF BMC Mix	0.511	Light	29	36	0.81	TRUE	32.257	0.02	0.083	NA	NA
ZF BMC Mix	1.02	Light	33	36	0.92	TRUE	5.942	0.048	-0.8	NA	NA
ZF BMC Mix	2.041	Light	30	36	0.83	TRUE	32.472	0.943	0.091	NA	NA
ZF BMC Mix	4.08	Light	27	36	0.75	TRUE	21.587	0.151	-0.275	NA	NA
ZF BMC Mix	6.13	Light	17	36	0.47	FALSE	NA	NA	NA	NA	NA

ZF BMC Mix	8.16	Light	8	36	0.22	FALSE	NA	NA	NA	NA	NA
ZF BMC Mix	10.2	Light	1	36	0.03	FALSE	NA	NA	NA	NA	NA
ZF Equi-Mix	0	Dark	34	36	0.94	TRUE	75.27	1	0	NA	NA
ZF Equi-Mix	1.5	Dark	33	36	0.92	TRUE	66.803	0	-0.112	YES	HYPO
ZF Equi-Mix	3	Dark	34	36	0.94	TRUE	61.908	0	-0.178	YES	HYPO
ZF Equi-Mix	3.6	Dark	29	36	0.81	TRUE	69.203	0	-0.081	YES	NA
ZF Equi-Mix	4.2	Dark	26	36	0.72	TRUE	78.387	0	0.041	YES	NA
ZF Equi-Mix	5.1	Dark	19	36	0.53	FALSE	NA	NA	NA	NA	NA
ZF Equi-Mix	6	Dark	13	36	0.36	FALSE	NA	NA	NA	NA	NA
ZF Equi-Mix	7.5	Dark	3	33	0.09	FALSE	NA	NA	NA	NA	NA
ZF Equi-Mix	0	Light	34	36	0.94	TRUE	3.802	1	0	NA	NA
ZF Equi-Mix	1.5	Light	33	36	0.92	TRUE	8.432	0.105	1.218	NA	NA
ZF Equi-Mix	3	Light	34	36	0.94	TRUE	27.435	0.74	6.217	NA	NA
ZF Equi-Mix	3.6	Light	29	36	0.81	TRUE	24.892	0.032	5.548	NA	NA
ZF Equi-Mix	4.2	Light	26	36	0.72	TRUE	9.415	0.941	1.477	NA	NA
ZF Equi-Mix	5.1	Light	19	36	0.53	FALSE	NA	NA	NA	NA	NA
ZF Equi-Mix	6	Light	13	36	0.36	FALSE	NA	NA	NA	NA	NA
ZF Equi-Mix	7.5	Light	3	33	0.09	FALSE	NA	NA	NA	NA	NA
